# Evaluation of 6 MALDI-Matrices for 10 μm Lipid Imaging and On-Tissue MSn with AP-MALDI-Orbitrap

**DOI:** 10.1021/jasms.1c00327

**Published:** 2022-03-31

**Authors:** Tina B. Angerer, Jerome Bour, Jean-Luc Biagi, Eugene Moskovets, Gilles Frache

**Affiliations:** † Luxembourg Institute of Science and Technology (LIST), Advanced Characterization platform, Materials Research and Technology, 41, rue du Brill, L-4422 Belvaux, Luxembourg; ‡ MassTech, Inc., Columbia, Maryland 21046, United States

**Keywords:** AP-MALDI, mass spectrometry imaging, lipids, tandem-MS, sample preparation

## Abstract

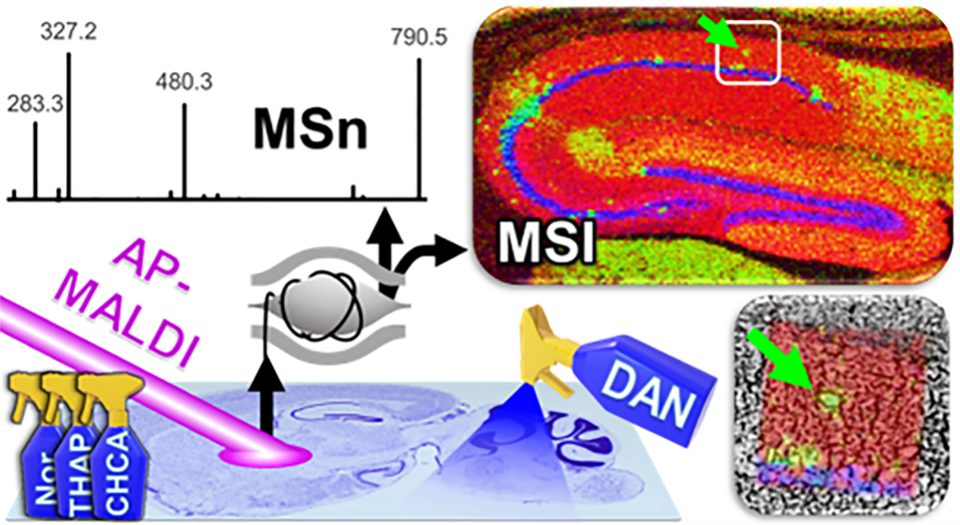

Mass spectrometry imaging is a technique uniquely suited to localize and identify lipids in a tissue sample. Using an atmospheric pressure (AP-) matrix-assisted laser desorption ionization (MALDI) source coupled to an Orbitrap Elite, numerous lipid locations and structures can be determined in high mass resolution spectra and at cellular spatial resolution, but careful sample preparation is necessary. We tested 11 protocols on serial brain sections for the commonly used MALDI matrices CHCA, norharmane, DHB, DHAP, THAP, and DAN in combination with tissue washing and matrix additives to determine the lipid coverage, signal intensity, and spatial resolution achievable with AP-MALDI. In positive-ion mode, the most lipids could be detected with CHCA and THAP, while THAP and DAN without additional treatment offered the best signal intensities. In negative-ion mode, DAN showed the best lipid coverage and DHAP performed superiorly for gangliosides. DHB produced intense cholesterol signals in the white matter. One hundred fifty-five lipids were assigned in positive-ion mode (THAP) and 137 in negative-ion mode (DAN), and 76 peaks were identified using on-tissue tandem-MS. The spatial resolution achievable with DAN was 10 μm, confirmed with on tissue line-scans. This enabled the association of lipid species to single neurons in AP-MALDI images. The results show that the performance of AP-MALDI is comparable to vacuum MALDI techniques for lipid imaging.

## Introduction

Mass spectrometry imaging (MSI) is a technique capable of locating and identifying atoms and molecules in a sample. By scanning across a surface and recording individual mass spectra at each location, MSI generates “chemical maps” which display the distribution of all detected species on a sample surface. MSI was first performed in 1949,^1^ but it has only gained traction in the last 20 years. With an ever-growing number of techniques emerging, MSI is now regularly applied in a variety of fields, ranging from inorganic materials science to biomedical research. Especially for biologists, MSI is of great interest because it is now possible to analyze intact macromolecules (e.g., proteins, lipids, and neurotransmitter)^2^ at cellular resolutions^3^ while small molecules and atoms can be detected in cell organelles.^4^ Multimodal experiments connect proteomic and lipidomic data and deepen our understanding of biological processes.^5−7^ The capabilities, applications, and drawbacks of MSI techniques are described in several reviews.^8−12^

Lipidomics is of ever-growing importance in the medical field.^13^ The changes in lipid compositions due to the onset of disease have been recognized, but traditional techniques, such as liquid chromatography mass spectrometry (LC–MS), fail to capture the complexity of heterogeneous samples (e.g., tumors). In contrast to proteins, single lipids cannot be labeled easily. MSI is uniquely suited to localize individual lipid species in a sample, as it distinguishes lipids based on their accurate mass. This improves upon unspecific dyes and techniques requiring sample homogenization (e.g., LC) but is insufficient to determine the exact species, since a number of different lipids can be present within a narrow mass range and lipids can have isomers, even from different classes. For this reason, an increasing number of MSI devices offer high-resolution mass analyzers and tandem-MS capability for structural identification to enhance specificity.^14^ With these capabilities, MSI has significantly contributed to the field of lipidomics by revealing lipid alterations in various diseases.^15−18^

Matrix-assisted laser desorption ionization (MALDI)-MSI is a technique where a matrix is applied to a sample surface and molecules of interest are extracted, embedded in matrix crystals, desorbed with a laser beam, ionized, and finally detected with a mass analyzer. The detectable species largely depend on the applied matrix, of which there are numerous options. In general, a MALDI matrix must be able to absorb the laser light and transfer charges to/from the target molecules, usually be vacuum stable, and in the case of MSI, form (sub)micron-sized crystals to enable highly localized detection. The achievable spatial resolution depends on the target molecules, the matrix properties, and the laser beam focus. For lipids, 5–10 μm has been demonstrated, and proteins are usually imaged at 50–100 μm (better spatial resolutions for lipids and proteins have been demonstrated with, e.g., t-MALDI-2).^15,19−22^

MALDI performed under ambient conditions (atmospheric pressure, AP-MALDI) removes the requirement of a vacuum stable matrix and enables the use of more volatile substances, such as 2′,6′-dihydroxyacetophenone (DHAP) and 2,4,6-trihydroxyacetophenone (THAP). Woods et al. state that the addition of heptafluorobutyric acid (HFBA) stabilizes DHAP in vacuo, but recent reports show its sublimation, even with added HFBA.^23,24^ An added benefit of AP-MALDI is that the tissue sample is not subjected to the harsh vacuum conditions leading to drying and cracking, which makes subsequent procedures such as histological tissue staining more likely to succeed.^25−29^ The downside is the shortened mean free path for the generated ions, possibly leading to their neutralization and diminishing sensitivity. This can reduce the useful spatial resolution and the ability to perform tandem-MS. Previous reports investigated AP-MALDI capabilities^30^ and have compared the performance of several matrices for AP-MALDI imaging,^24,31−35^ but to our knowledge, there is currently no comprehensive report comparing the numerous matrices available and their lipid coverage on the same sample type and device in positive- and negative-ion mode.

Therefore, in this study we tested 11 sample preparation protocols with six different matrices, washing steps, and additives that have previously been reported to yield good results for high spatial resolution lipid imaging with vacuum and AP-MALDI techniques. Protocols were adapted for the matrices: α-cyano-4-hydroxycinnamic acid (CHCA),^36,37^ norharmane (Nor),^36,38^ 1,5-diaminonapthalene (DAN),^24,39^ DHAP,^23,24^ THAP,^40^ and 2′,5′-dihydroxybenzoic acid (DHB),^38,41^ and their performance was evaluated, in terms of signal intensity/ability to perform tandem-MS, lipid coverage and achievable, useful spatial resolution, in positive and negative ion mode. Additionally, 76 peaks were identified using on tissue tandem-MS.

## Materials and Methods

### Chemicals

Chemicals and solvents (analytical grade) were purchased from the following sources: α-cyano-4-hydroxycinnamic acid 98% (CHCA) (Sigma-Aldrich), 2,5-dihydroxybenzoic acid 98% (DHB) (Sigma-Aldrich), norharmane 98% (Nor) (Acros Organics), 1,5-diaminonaphthalene 97% (DAN) (Sigma-Aldrich), 2′,4′,6′-trihydroxyacetophenone 99.5% (THAP) (Sigma-Aldrich), 2,6-dihydroxyacetophenone 99.5% (DHAP) (Sigma-Aldrich), acetonitrile (ACN) (Honeywell), chloroform (Acros Organics), methanol (Carl Roth), ammonium acetate (AmAc) (VWR). ammonium sulfate (AmS) (Sigma-Aldrich), heptafluorobutyric acid (HFBA) (Sigma-Aldrich), and trifluoroacetic acid (TFA) (Sigma-Aldrich). All chemicals used in this study were stored, handled, and disposed of according to good laboratory practices (GLP).

### Sample Preparation

Ten micrometer sagittal mouse brain sections on indium tin oxide (ITO)-coated glass slides (Diamond Coatings, UK) were prepared at Swansea University, as stated in a recent publication.^43^ Sections were kept at −80 °C until analysis and dried in a vacuum desiccator for 30 min prior to matrix application. Optical images of the tissue sections were taken using an Olympus BX51 microscope (Olympus, Belgium). On some sections, tissue washing was performed with AmAc at 50 mM concentration, chilled to 4 °C, for 3 × 5 s, as described previously.^42^ Serial sections on separate ITO glass slides were coated with the various matrices using an HTX TM sprayer (HTX Technologies LLC, USA), flow rate 0.12 mL/min, velocity 1200 mm/min, drying time 2 s, line spacing 2.5 mm. Matrix composition and additives, sprayer temperature, number of matrix layers/passes, and laser settings are listed in Table 1. The analyses showing the best performance (most lipids detected, best signal) were repeated on different days in positive-ion mode for CHCA, THAP, and DAN70 and in negative-ion mode for DAN70.

**Table 1:** Overview of Matrix Recipes, Sprayer/Laser Settings, and Experiments Performed^a^

matrix (mg/mL)	solvents	add/ sample prep	temp (°C)	*Z*	laser (40/20 μm)	laser (10 μm)	Hip.	Crb.	Str.	adapted from
CHCA (5)	CHCl_3_:MeOH 1:1	0.2%TFA	40	16	3000 Hz 10%	3000 Hz 2.5%	× (+)	× (±)	× (+)	Barré et al.^36^
										Hochart et al.^37^
Nor (7)	CHCl_3_:MeOH 2:1		30	12	500 Hz 10%			× (±)	× (+)	Barré et al.^36^
DHB (10)	MeOH:H_2_O 7:3	0.1%TFA	50	8	3000 Hz 10%			× (+)	× (+)	McMillen et al.^38^
										Leopold et al.^41^
DHAP (10)	ACN:AmS^b^ 7:3	0.05% HFBA	50	8	3000 Hz 7.5%		× (−)^c^	× (−)	× (+)	Jackson et al.^24^
										Colsch et al.^23^
THAP (10)	ACN:AmS^b^ 7:3	0.05% HFBA	50	8	3000 Hz 15%	3000 Hz 10%	× (+) × (−)^c^	× (±)	× (+)	Pieles et al.^40^
THAPnS (10)	ACN:H_2_O 7:3	0.05%HFBA	50	8	3000 Hz 15%			× (+)		Pieles et al.^40^
DAN70 (10)	ACN:H_2_O 7:3		30	8	3000 Hz 5%	2000 Hz 1.5%	× (±)	× (±)	× (+)	Sun et al.^39^
DAN90 (10)	ACN:H_2_O 9:1		30	8	3000 Hz 5%			× (+)		Jackson et al.^24^
DANtfa (10)	ACN:H_2_O 7:3	0.1%TFA	30	8	3000 Hz 5%			× (+)		Sun et al.^39^
DANhfba (10)	ACN:H_2_O 7:3	0.05%HFBA	30	8	3000 Hz 5%			× (+)		Sun et al.^39^
										Colsch et al.^23^
DANw (10)	ACN:H_2_O 9:1	AmAc wash	30	8	3000 Hz 5%	2000 Hz 1.5%	× (+)	× (±)		Sun et al.^39^
										Angel et al.^42^

a Listed are matrices + matrix concentration (mg/mL), matrix solvents, matrix additives or sample preparation, HTX-TM sprayer temperature (°C), matrix layers/passes (*Z*), laser frequency (Hz), and intensity (%) for 40/20 and adjusted settings for 10 μm imaging, ion-mode (±) included for each analyzed brain area (Hip. = hippocampus, spatial resolution 10/20^c^ μm; Crb. = cerebellum, 40 μm; Str. = striatum, 40 μm), and matrix recipe references (adapted from).

b 125 mM (NH_4_)_2_SO_4_ in H_2_O.

c Hippocampus imaging with THAP/DHAP in negative ion mode performed at 20 μm spatial resolution.

#### AP-MALDI-MSI

MALDI analysis on brain sections was performed using an AP-MALDI UHR ion source (Masstech, Inc., USA), which has been described in detail elsewhere,^25,26^ coupled to an LTQ/Orbitrap Elite high-resolution mass spectrometer (Thermo-Fisher Scientific, USA) in positive- and negative-ion mode. For imaging, the AP-MALDI source was operated in “Constant Speed Raster” motion mode. Whole, sagittal brain sections were analyzed in positive and negative ion mode at 40 μm stepping size (matrix: Norhamane), as well as the cerebellum (Crb., 40 μm), the hippocampus (Hip., 10/20 μm), and a small area in the striatum (Str., 40 μm). Matrices, ion modes, and laser settings used for each analysis are summarized in Table 1. The laser spot size was <10 μm for the hippocampus analysis and <40 μm for all other analyses (20 μm max.). A laser focus of 8.38 μm was determined with scanning electron microscopy (SEM, Figure S3), and using the camera in the source and comparing line to line signal intensities, settings were adjusted for each measurement to ablate as much matrix as possible without oversampling. Spectrum acquisition: 800 ms maximum injection time; mass range: 500–2000 Da (250–1000 Da for striatum (cholesterol) imaging); mass resolution: 120k at *m*/*z* 400. Tandem-MS was performed on 76 peaks (Table 2): 1 Da isolation window, and collision-induced dissociation/higher-energy collision dissociation (CID/HCD) was performed with collision energies of 20–55%, adjusted for each lipid species individually. Tandem-MS scans were summed up over 30–120 s. Details on matrix used, collision mode, and energy can be found in the scan header of each analysis in Supporting Information 2. Data analysis and visualization were performed with Thermo Xcalibur 2.2 and Thermo ImageQuest (Thermo-Fisher Scientific, USA), METASPACE,^44^ MSiReader 1.2 (NC State University, USA),^45^ LipostarMSI (Molecular Horizons Srl, Italy),^46^ and OriginPro 2019b (OriginLab Corp., USA). All images are normalized to total ion count (TIC). Lipid identification was performed with METASPACE for hippocampus images (database: LIPIDMAPS, FDR: 20%) and LipostarMSI for cerebellum images (database: LIPIDMAPS, mass accuracy: 2 ppm; mass and isotopic pattern score: 80%+).

**Table 2:** Lipids Identified with Tandem MS in Positive- and Negative-Ion Mode^a^,^b^

Positive-Ion Mode					
assignment	ion species	formula	*m*/*z* (detected)	*m*/*z* (exact)	Δppm
PC(16:0/9:0(OH))	[M + H]^+^	C_33_H_65_O_9_NP	650.4372	650.4392	3.00
PC(16:0/9:0(COOH))	[M + H]^+^	C_33_H_65_O_10_NP	666.4321	666.4341	2.94
PC(18:0/9:0(OH))	[M + H]^+^	C_35_H_69_O_9_NP	678.4698	678.4705	0.96
PC(16:0/16:0)-TMA	[M + K]^+^	C_37_H_71_O_8_PK	713.4497	713.4518	2.95
PDME(16:0/16:0)	[M + H]^+^	C_39_H_79_O_8_NP	720.5543	720.5538	–0.72
SM(d36:1)	[M + H]^+^	C_41_H_84_O_6_N_2_P	731.606	731.6062	0.21
PC(16:0/16:0)	[M + H]^+^	C_40_H_81_O_8_NP	734.5689	734.5694	0.72
PC(16:0/16:0)	[M + Na]^+^	C_40_H_80_O_8_NPNa	756.553	756.5514	–2.14
PC(16:0/18:1)	[M + H]^+^	C_42_H_83_O_8_NP	760.5826	760.5851	3.26
PC(16:0/16:0)	[M + K]^+^	C_40_H_80_O_8_NPK	772.5254	772.5253	–0.12
PC(16:0/18:1)	[M + Na]^+^	C_42_H_82_O_8_NPNa	782.5685	782.5670	–1.88
HexCer(d18:1/22:1)	[M + H]^+^	C_46_H_88_O_8_N	782.6489	782.6505	1.98
SM(d40:2)	[M + H]^+^	C_45_H_90_O_6_N_2_P	785.6534	785.6531	–0.38
PC(18:1/18:1)	[M + H]^+^	C_44_H_85_O_8_NP	786.6004	786.6007	0.42
PC(18:1/18:0)	[M + H]^+^	C_44_H_87_O_8_NP	788.6161	788.6164	0.36
PE(40:7)*	[M + H]^+^	C_45_H_77_O_8_NP	790.5357	790.5381	3.07
PE(18:0/22:6)	[M + H]^+^	C_45_H_79_O_8_NP	792.5555	792.5538	–2.17
PC(16:0/18:1)	[M + K]^+^	C_42_H_82_O_8_NPK	798.5403	798.5410	0.83
PC(16:0/22:6)	[M + H]^+^	C_46_H_81_O_8_NP	806.5691	806.5694	0.41
PE(38:4)	[M + K]^+^	C_43_H_78_NO_8_PK	806.5067	806.5097	3.67
PC(18:1/20:4)	[M + H]^+^	C_46_H_83_O_8_NP	808.5839	808.5851	1.45
HexCer(d18:1/24:2)	[M + H]^+^	C_48_H_90_O_8_N	808.6642	808.6661	2.34
SM(d42:2)	[M + H]^+^	C_47_H_94_O_6_N_2_P	813.6846	813.6844	–0.25
PC(18:1/18:0)*	[M + K]^+^	C_44_H_86_O_8_NPK	826.5739	826.5723	–1.98
PC(18:0/22:6)	[M + H]^+^	C_48_H_85_O_8_NP	834.6012	834.6007	–0.56
PC(38:6)	[M + K]^+^	C_46_H_80_NO_8_PK	844.5228	844.5253	2.97
PC(38:4)	[M + K]^+^	C_46_H_84_O_8_NPK	848.5556	848.5566	1.19
HexCer(d18:1/24:1(2OH))	[M + Na]^+^	C_48_H_91_O_9_NNa	848.6564	848.6586	2.59
PC(40:6)	[M + K]^+^	C_48_H_85_O_8_NPK	872.5565	872.5566	0.13
PC(16:0/18:1)+PC(16:0/16:0)	[M+M+H]^+^	C_82_H_163_O_16_N_2_P_2_	1494.1488	1494.1470	–1.04
PC(16:0/18:1)	[2M+H]^+^	C_84_H_165_O_16_N_2_P_2_	1520.1604	1520.1630	1.64
PC(18:1/18:0)+PC(16:0/18:1)	[M+M+H]^+^	C_86_H_169_O_16_N_2_P_2_	1548.1868	1548.1940	4.64

Negative-Ion Mode					
assignment	ion species	formula	*m*/*z*(measured)	*m*/*z*(exact)	ppm
Cer(d18:0/18:1)	[M – H]^−^	C_36_H_70_O_3_N	564.535	564.5350	0.04
CerP(d18:1/18:0)	[M – H]^−^	C_36_H_71_NO_6_P	644.5019	644.5025	0.85
PA(16:0/16:1)	[M – H]^−^	C_35_H_66_O_8_P	645.4503	645.4501	–0.34
PA(16:0/16:0)	[M – H]^−^	C_35_H_68_O_8_P	647.4645	647.4657	1.9
CerP(d38:2)	[M – H]^−^	C_38_H_73_O_6_NP	670.5173	670.5181	1.19
PA(16:0/18:1)	[M – H]^−^	C_37_H_70_O_8_P	673.4795	673.4814	2.79
SM(d34:1)	[M – CH_3_]^−^	C_38_H_76_O_6_N_2_P	687.5426	687.5447	2.98
SM(d18:1/18:0)	[M – CH_3_]^−^	C_40_H_80_O_6_N_2_P	715.575	715.5760	1.33
PE(16:0/18:0)	[M – H]^−^	C_39_H_77_O_8_NP	718.5377	718.5392	2.13
PE(P-18:1/18:1)	[M – H]^−^	C_41_H_77_O_7_NP	726.5427	726.5443	2.22
PE(P-18:0/18:1)	[M – H]^−^	C_41_H_79_O_7_NP	728.5584	728.5600	2.14
PE(18:1/18:1)	[M – H]^−^	C_41_H_77_O_8_NP	742.5391	742.5392	0.17
PC(16:0/18:1)	[M – CH_3_]^−^	C_41_H_79_O_8_NP	744.5535	744.5549	1.85
PA(18:0/22:6)	[M – H]^−^	C_43_H_72_O_8_P	747.4959	747.4970	1.51
PE(P-18:1/20:1)	[M – H]^−^	C_43_H_81_O_7_NP	754.5749	754.5756	0.94
PE(18:0/20:4)	[M – H]^−^	C_43_H_77_O_8_NP	766.5373	766.5392	2.52
SM(d18:1/22:0)	[M – CH_3_]^−^	C_44_H_88_O_6_N_2_P	771.637	771.6386	2.01
PE(P-18:0/22:6)	[M – H]^−^	C_45_H_77_O_7_NP	774.5427	774.5443	2.08
PE(18:0/20:4(OH)	[M – H]^−^	C_43_H_77_O_9_NP	782.5347	782.5341	–0.72
PC(16:1/22:6)	[M – CH_3_]^−^	C_45_H_75_O_8_NP	788.5244	788.5236	–1.04
PS(18:1/18:0)	[M – H]^−^	C_42_H_79_O_10_NP	788.5447	788.5447	0.01
PE(18:0/22:6)	[M – H]^−^	C_45_H_77_O_8_NP	790.5374	790.5392	2.31
HexCer(d18:1/22:0(2OH))	[M – H]^−^	C_46_H_88_O_9_N-	798.6443	798.6465	2.70
C18:1-Sulf	[M – H]^−^	C_42_H_80_O_11_NS	806.5436	806.5458	2.68
C18(OH)-Sulf	[M – H]^−^	C_42_H_80_O_12_NS	822.5411	822.5407	–0.52
HexCer(d18:1/24:0(2OH))	[M – H]^−^	C_48_H_92_O_9_N	826.6757	826.6778	2.49
PS(18:0/22:6)	[M – H]^−^	C_46_H_77_O_10_NP	834.5274	834.5291	1.99
PS(18:1/22:0)	[M – H]^−^	C_46_H_87_O_10_NP	844.6081	844.6073	–0.93
PI(16:0/20:4)	[M – H]^−^	C_45_H_78_O_13_P	857.518	857.5186	0.64
C22-Sulf.	[M – H]^−^	C_46_H_88_O_11_NS	862.6062	862.6084	2.50
PI(18:0/18:1)	[M – H]^−^	C_45_H_84_O_13_P	863.5636	863.5655	2.20
PS(18:1/24:0)	[M – H]^−^	C_48_H_91_O_10_NP	872.6341	872.6386	5.12
C22(OH)-Sulf	[M – H]^−^	C_46_H_88_O_12_NS	878.6008	878.6033	2.81
PI(18:1/20:4)	[M – H]^−^	C_47_H_80_O_13_P	883.5321	883.5342	2.38
PI(18:0/20:4)	[M – H]^−^	C_47_H_82_O_13_P	885.5493	885.5499	0.62
C24:1-Sulf.	[M – H]^−^	C_48_H_90_O_11_NS	888.6228	888.6240	1.36
C24-Sulf.	[M – H]^−^	C_48_H_92_O_11_NS	890.6376	890.6397	2.31
PI(18:0/20:4(OH))	[M – H]^−^	C_47_H_82_O_14_P	901.5425	901.5448	2.52
C24(OH)-Sulf.	[M − H]^−^	C_48_H_92_O_12_NS	906.6333	906.6346	1.40
GA1(d36:1)	[M – H]^−^	C_61_^13^CH_113_O_23_N_2_	1254.777	1254.7770	0.26
GM1(d36:1)	[M – H]^−^	C_73_H_130_O_31_N_3_	1544.8632	1544.8690	4.00
GM1(d38:1)	[M – H]^−^	C_74_^13^C H_134_O_31_N_3_	1573.899	1573.9040	3.19
GD1(d36:1)	[M – H]^−^	C_84_H_147_O_39_N_4_	1835.957	1835.9648	4.24
GD1(d36:1)	[M – 2H + K]^−^	C_84_H_146_O_39_N_4_K	1873.916	1873.9210	2.71

a Listed are assigned lipid, ion species, chemical formular, *m*/*z* value from tandem-MS spectra (detected) and calculated (exact) from the formula, and mass deviation (Δppm). Abbreviations for all lipid species listed at the bottom of the table. Tandem-MS spectra for each lipid shown in Supporting Information 2. Spatial distribution in MS-images can be viewed in METASPACE.

b Phosphatidylcholine (PC), phosphatidyl-dimethylethanolamine (PDME), sphingomyelin (SM), hexosylceramide (HexCer), ceramide (Cer), ceramide 1-phosphate (CerP), phosphatidylethanolamine (PE), phosphatidylserine (PS); phosphatidic acid (PA), sulfatide (Sulf), phosphatidylinositol (PI), asialo-/monosialo-/disialo-ganglioside (GA/GM/GD); “P-“ plasmanyl-ether lipid; “d” 1,3-dihydroxy, long-chain base in sphingolipids.

#### ToF-SIMS-MSI

ToF-SIMS analysis was performed using an TOF.SIMS 5 (IONTOF GmbH, Germany) with a 25 kV Bi_3_^+^ primary analysis beam. Dried brain sections with DAN70 matrix were analyzed in burst alignment, delayed extraction, positive-ion mode with a total primary ion dose of 7 × 10^11^ ions/cm^2^, cycle time 105 us, random raster mode, 1 frame/patch, 1 shot/frame/pixel, 25 scans, mass range: 1–1000 Da, mass resolution: 5000 at *m*/*z* 300, image size: 256 × 256 μm and 512 × 512 pixels, spatial resolution: 0.5 μm/pixel. Data analysis and visualization was performed using SurfaceLab 7 (IONTOF GmbH, Germany).

#### SEM

SEM analysis was performed using a Quanta 200 field emission gun scanning electron microscope (FEG SEM, Philips-FEI, USA). DAN70 matrix analysis on brain was performed in a low-vacuum environment (60 Pa) with a “Large Field Detector” (LFD) for a topographical image. CHCA matrix analysis was performed with a Genesis XM 4i energy dispersive spectrometer (from EDAX) system for elemental mapping and line-scans, with a back-scattered, composition mode detector called BSED (for high vacuum), generating a grayscale, chemical composition SEM image, with “heavier” elements areas corresponding to brighter areas.

## Results

**Figure 1:**
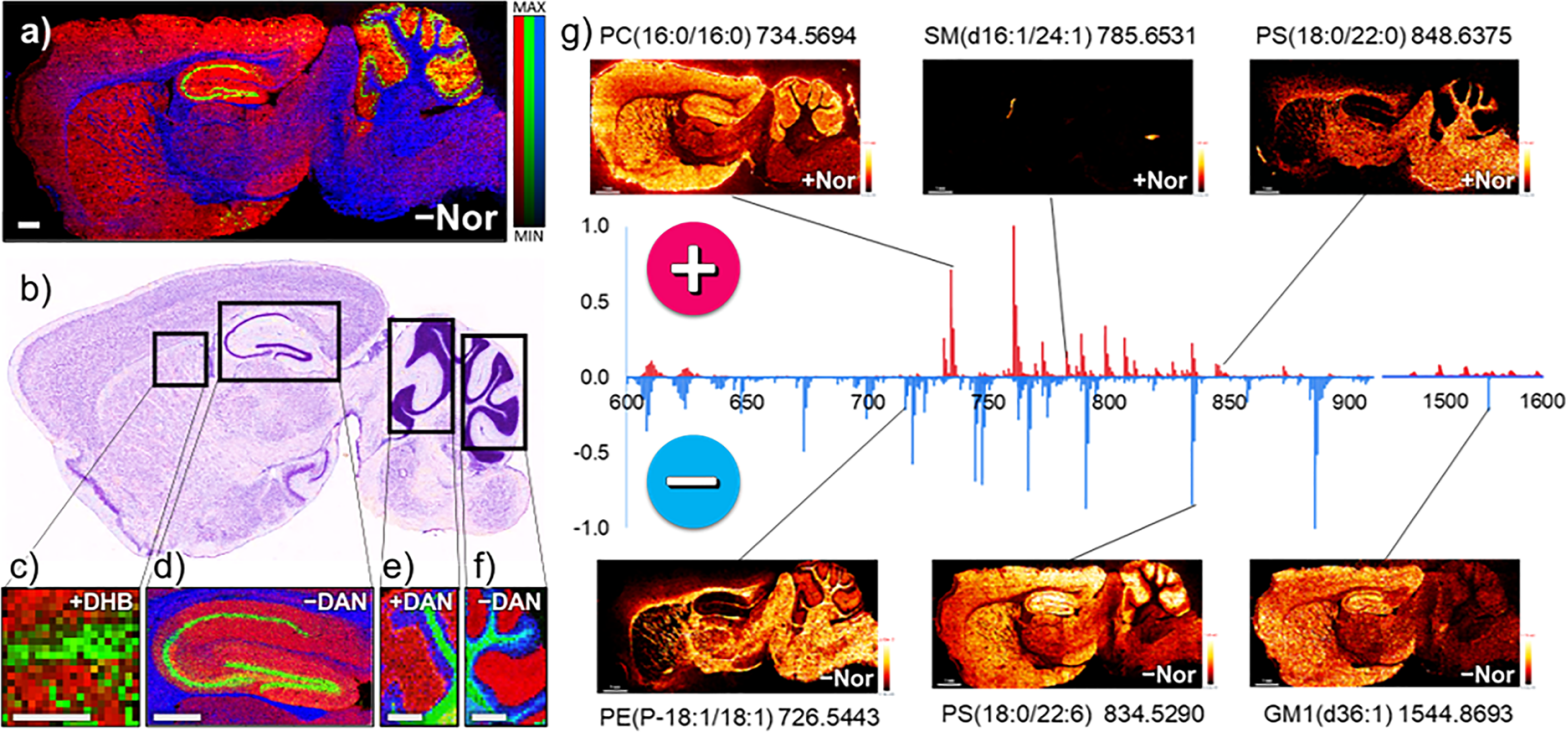
AP-MALDI imaging on sagittal brain section. (a) Full brain AP-MALDI-image, 40 μm spatial resolution, (red: PS(40:6) green: PI(38:5), blue: C24:1-Sulf), (b) H&E stained sagittal mouse brain section (Allen Developing Mouse Brain Atlas, data set P56, sagittal), (c–f) small area AP-MALDI-images of (c) fiber tracts/striatum images for cholesterol analysis (40 μm, red: PC(32:0) green: cholesterol), (d) hippocampus imaged at 10 μm, (red: PS(40:6) green: PI(38:5), blue: C24:1-Sulf), (e) cerebellum in positive ion mode (red: SM(d36:1), green: HexCer(d40:2), blue: PC(34:1)), and (f) negative-ion mode (40 μm, red: SM(d36:1), green: C24:1-Sulf, blue: PI(38:4)). Matrix and ion mode stated in each image, scalebar: 500 μm. (g) Six representative lipid images and spectra in positive-ion and (mirroring) in negative-ion mode.

**Figure 2:**
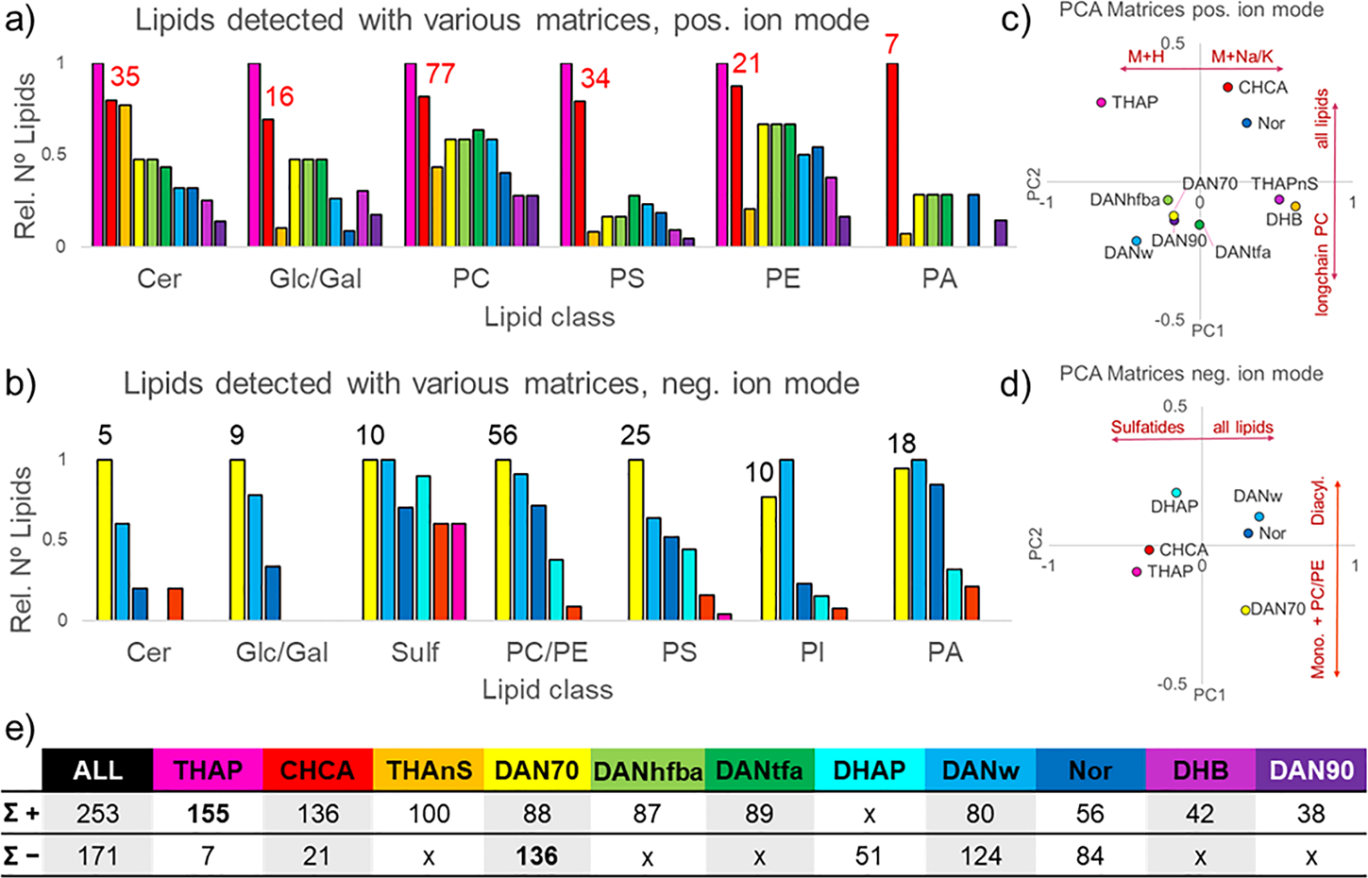
Lipid coverage for various matrices with AP-MALDI. (a) Number of identified lipids by class, detected in the cerebellum using five matrices (10 sample preparation protocols) in positive ion mode and (b) five matrices (six sample preparation protocols) in negative-ion mode listed in e). Displayed in the graph is the relative number of lipids normalized to the highest number of detected lipids for each class; the actual number is stated above CHCA (pos) and DAN70 (neg). Scores plots (PC1 vs PC2) of PCA analysis for cerebellum mass spectra in (c) positive- and (d) negative-ion mode (loadings plots: Figure S7). (e) Sum of assigned lipids for each matrix in positive- and negative-ion mode. Σ+ (x) DHAP was not analyzed in positive ion mode, Σ– (x) all matrices were analyzed in negative-ion mode but excluded if insufficient signal was detected (below 1 × 10^3^ counts). Lipids were assigned using LipostarMSI.

**Figure 3:**
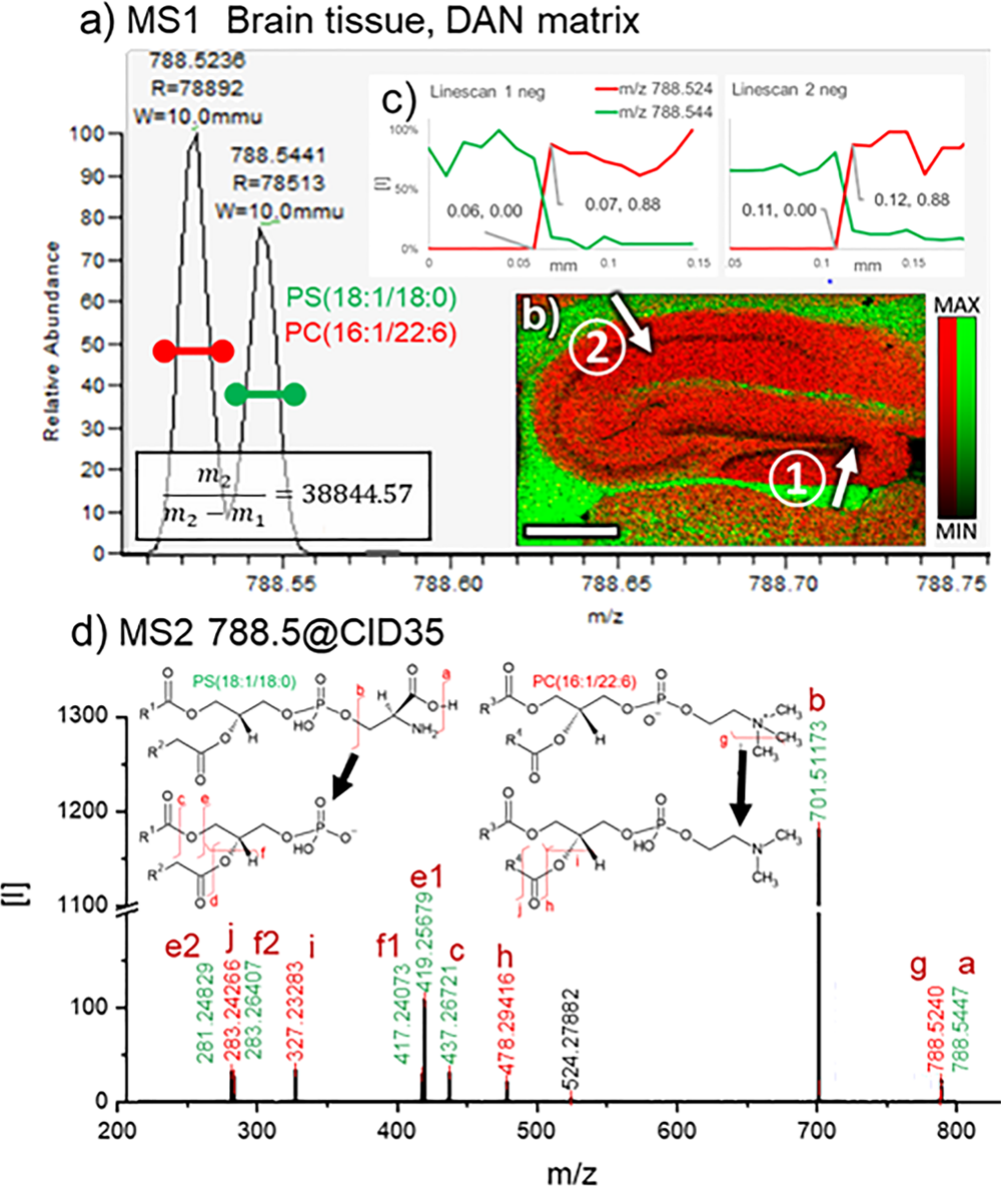
Capabilities of the AP-MALDI-Orbitrap system demonstrated on the hippocampus, (a) MS1 scan on brain tissue (matrix: DAN70, negative ion mode, showing *m*/*z* 788.5447 and *m*/*z* 788.5236. (b) Distribution of PS(18:1/18:0) (green) and PC(16:1/22:6)–CH_3_ (red) in a 10 μm AP-MALDI image, white arrows show the positions of linescans in (c) scale bar: 500 μm. (c) Two linescans, normalized to their individual maximum intensity (100%). (d) On-tissue tandem-MS analysis of *m*/*z* 788.5 ± 0.5 Da, containing PS (green) and PC (red) fragments. Fragmentation mechanism shown for both lipids, referring to the letters above each mass peak.

**Figure 4:**
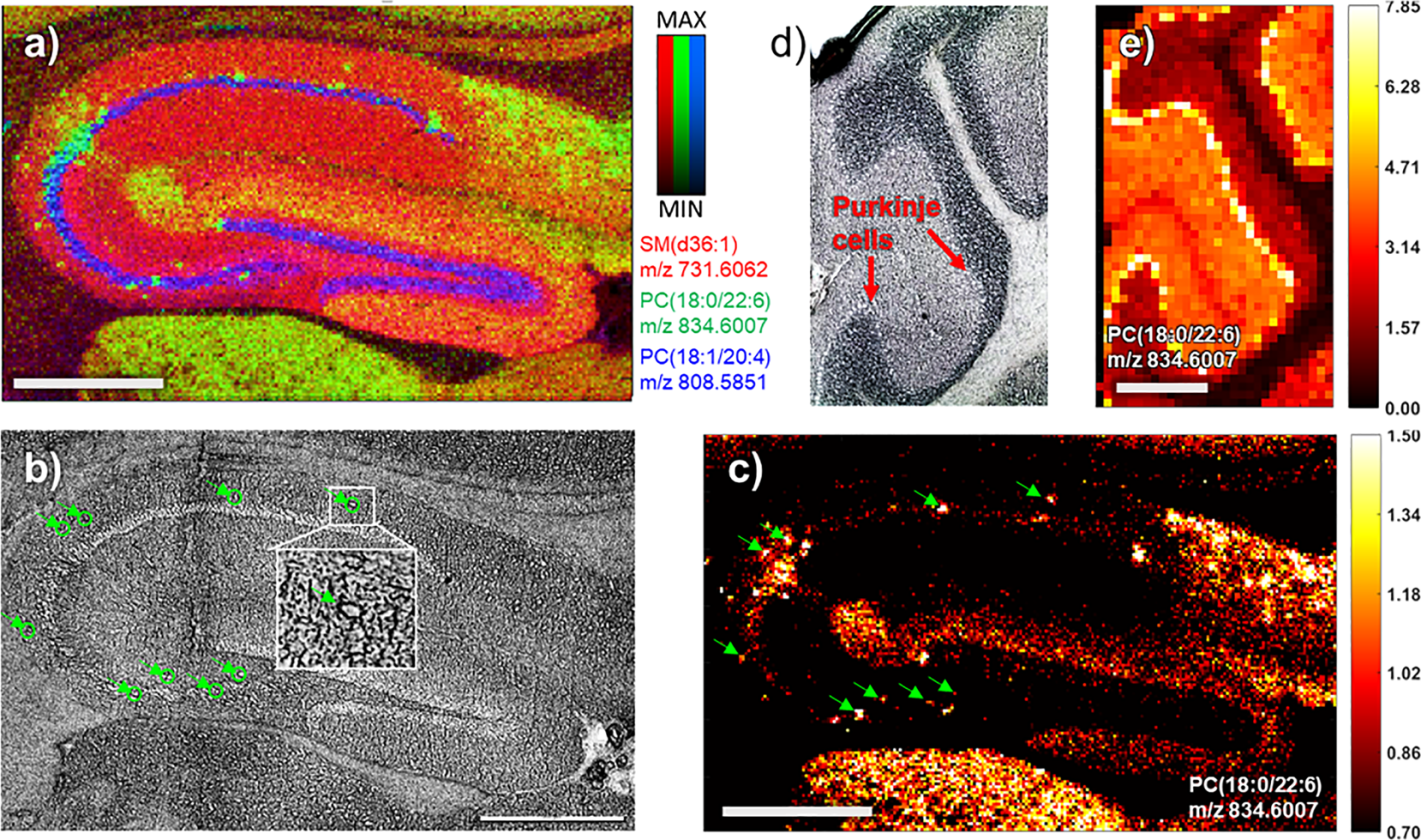
Single-cell imaging in the hippocampus with AP-MALDI. (a) 10 μm Hippocampus analysis (matrix: DAN70, positive-ion mode), RGB overlay of: SM(d36:1) red, gray matter; PC(18:0/22:6) green, single cells; PC(18:1/20:4) blue, pyramidal layer. (b) Microscope image of the hippocampus pre matrix application, features corresponding to the distribution of PC(18:0/22:6) highlighted in green. (c) Single-ion image of PC(18:0/22:6) with the same features highlighted as in b), color scale: hot. (d) Microscope image of the cerebellum pre matrix application. (e) Single-ion image of PC(18:0/22:6) in the cerebellum, spatial resolution: 40 μm, color scale: hot. All scale bars: 500 μm.

The aim of this study was to evaluate the performance of various matrices/sample preparation protocols for AP-MALDI in terms of lipid coverage, signal intensity, and high-resolution imaging capability. For this purpose, several images were taken on sagittal mouse brain sections. Brain sections are often used in comparison studies, as they are rich in a great variety of lipids and the results can be compared to previously published material and entries in databases like, e.g., METASPACE. Figure 1 shows an overview of the data sets included in this study (summarized in Table 1): Full brain sections (Figure 1a, spatial resolution: 40 μm) were imaged to obtain a detailed overview of the lipid distributions in the brain and to determine the ideal location to perform tandem-MS on specific lipids. The location of six lipid species and representative spectra in positive- and negative-ion mode are shown in Figure 1g. Evidently, for species like SM(d16:1/24:1) is it important to know the location prior to on-tissue tandem-MS analysis as it is only present in the ventricles (Figure 1g). The full brain data sets are available in METASPACE: AP_MALDI_Full_Brain. The ability to detect cholesterol was tested on a small area in the striatum, around the fiber tracts (Figure 1c), with a resolution of 40 μm, in positive-ion mode, and a mass range of 250–1000 Da. To test instrument and matrix performance for higher spatial resolution imaging, the hippocampus with its intricate structures was imaged at 10 μm (Figure 1d, Figure S2) in positive and negative ion mode (for ganglioside detection, DHAP and THAP analysis was performed at 20 μm to shorten analysis time). Hippocampus data sets in METASPACE: AP_MALDI_Hippocampus. To determine lipid coverage (Figure 2), the cerebellum was imaged in positive ion mode (Figure 1e, Figure S1) and negative ion mode (Figure 1f, Figure S1) at 40 μm spatial resolution. DAN matrix performed well in positive and negative ion mode and attempts were made to improve its performance further, including tissue washing (DANw) and acidic additives (DANtfa, DANhfba). Initially HFBA was reported to stabilize DHAP in vacuum which would make it unnecessary in atmospheric conditions.^23^ Since HFBA is also a strong acid, that similar to TFA is used as ion-pairing agent in liquid chromatography, we investigated its protonating and potentially ion enhancing effects.^47^ The biggest improvement to DAN performance for lipid detection was achieved by changing the solvent ratios of acetonitrile and water (ACN:H_2_O) from 9:1 (DAN90, 90% ACN) to 7:3 (DAN70, 70% ACN) without decreasing the achievable spatial resolution (demonstrated in Figure 3 and Figure 4). Cerebellum data sets in METASPACE: positive ion mode AP_MALDI_MATRIX_pos; negative ion mode AP_MALDI_MATRIX_neg. Images for all cerebellum and hippocampus analyses are displayed in the Supporting Information 1 (Figure S1 and S2 respectively). Brain tissue not imaged was used for on-tissue tandem-MS.

### Lipid coverage with various matrices

Figure 2 shows the number of lipid species detected in the cerebellum (Figure 1d/e) using all 11 sample preparation protocols for positive (Figure 2a) and negative (Figure 2b) ion mode, grouped into lipid classes (data was excluded if the most intense signals were below 1 × 10^3^ counts). Spectra for all included data sets in positive (Figure S4) and negative ion mode (Figure S5) and signal-to-noise ratios for several signals (Figure S6) can be found in the Supporting Information 1. Using LipostarMSI and the LIPIDMAPS database, lipids were putatively assigned (Figure 2a/e), with strict selection criteria (mass accuracy: 2 ppm; mass and isotopic pattern score: 80%+). The most species detected using one matrix were 155 in positive (THAP), 136 in negative ion mode (DAN70), and 224 for positive and negative ion mode combined (DAN70). Repeat measurements on different days for THAP (155 lipids 2020–12–01, 144 lipids 2020–10–19) and CHCA (136 lipids 2021–01–19, 124 lipids 2020–10–15) in positive ion mode, and DAN70 in positive (88 lipids 2020–10–07, 83 lipids 2021–01–25) and negative ion mode (136 lipids 2020–09–24, 120 lipids 2021–01–26) showed similar results (data sets included in the cerebellum METASPACE projects). Other parameters and/or databases (e.g., HMBD, SwissLipids) could lead to different results in terms of peak identity and the number of species, but this consistent approach was suitable to determine performance of each matrix/protocol.

In negative ion mode, DAN70 was the only matrix that enabled the detection of a broad range of lipid species while DHAP and THAP mainly produced sulfatide and ganglioside signals (Figure 2b/d). Tissue washing (DANw) did increase signal intensities about 1.5-fold and double the S/N ratio for certain peaks (Figure S6) compared to DAN70. Jackson et al. noted that DHAP is superior to DAN for detecting gangliosides, especially for intact GD1(d36:1) at *m*/*z* 1835.965. Similarly, in this study DHAP was the only matrix producing sufficient GD1 signal to perform on tissue tandem-MS (Supporting Information 2).

PCA analysis of the spectra (average sum spectra for the whole cerebellum image, as shown in Figure S1) shows that, in positive ion mode (Figure 2c, loadings plots in Figure S7), matrices CHCA, Nor and DHB produce higher intensity, sodiated/potassiated [M+Na/K]^+^ species while THAP and DAN (especially with AmAc wash, DANw) favor protonated [M + H]^+^ species. Also, DAN70 shows higher intensities for long chain fatty acid PC species. It has been reported that the addition of ammonium salts to a matrix, aids in suppressing sodium and potassium, especially for oligonucleotide analysis with THAP.^40,50^ Similarly, we observed that THAPnS (no salt added) produces almost exclusively [M+Na/K]^+^ species. We did not observe a drastic signal increase after tissue washing (DANw), as was previously reported,^42,51^ and no improvement in the number of detected species (Figure 2e). The slight decrease in the number of detected species can be explained by the lack of salt adducts which often cause a single lipid species to be detected 3 times in positive ion mode [M+H/Na/K]^+^. The addition of acids, used in the CHCA and THAP protocols, to DAN (DANtfa, DANhfba) did not improve performance either. Based on those results, we chose the matrices providing the best lipid coverage and the highest signal (CHCA, THAP and DAN70) to test the ultimate capabilities of our instrumental setup.

### Instrumentation capabilities: Spatial resolution, mass resolution and tandem-MS

The MassTech AP-MALDI source in combination with an Orbitrap mass analyzer and an HTX TM sprayer for sample preparation, allows to attain MS-imaging with 10 μm spatial resolution while collecting high resolution mass spectra (up to 240k FWHM at *m*/*z* = 400), and on-tissue tandem-MS spectra. Figure 3 shows the analysis of the hippocampus from a sagittal mouse brain section (also shown in Figure 1d) and lipid species PS(18:1/18:0)–H at *m*/*z* 788.5447 and PC(16:1/22:6)–CH_3_ at *m*/*z* 788.5236. To distinguish those lipids, a resolving power of about 40k is necessary, which is well within the capabilities of the Orbitrap (Figure 3a). PC(16:1/22:6) is exclusively located in the hippocampus while PS(18:1/18:0) is mainly in the surrounding fiber tracts (Figure 3b). Linescans across the hippocampus/fiber tract interface demonstrate that we can monitor chemical changes with a spatial resolution of 10 μm (Figure 3c, position of the linescans is shown in 2b, indicated with white arrows).

SEM images show that DAN70 matrix applied with an HTX-TM sprayer produces ∼1 μm crystals and that the AP-MALDI laser can be focused below 10 μm (Figure S3). At 10 μm spatial resolution sufficient signal is produced to assign 52 species with DAN70 in the hippocampus (72 with CHCA, 134 with THAP) in positive ion mode, and 121 species in negative ion mode (METASPACE, database: LIPIDMAPS, FDR:20%). Significantly more species are assigned in the THAP data set due to the slightly different analysis area which included the lateral ventricle with unique lipids. (The cerebellum data sets used to compare lipid coverage do not have this issue). In terms of spatial resolution, CHCA and DAN70 produce sharp images and perform better than THAP although all data sets were acquired with the same analysis settings (line spacing and scanning speed) and laser focus was <10 μm (Figure S2). The reason could be that laser settings were adjusted for maximum signal and were higher for THAP than for other matrices. Less laser energy would have provided less signal but could have resulted in a sharper image.

Figure 3d shows a tandem-MS spectrum of *m*/*z* 788.5, and it demonstrates that AP-MALDI produces sufficient lipid signal on tissue to perform tandem-MS analysis. PS(18:1/18:0) and PC(16:1/22:6) are fairly well spatially separated in the hippocampus but can overlap in other brain areas and are therefore both present in the tandem-MS spectrum (Figure 3d). Due to the complexity of biological samples, it is to be expected that a tandem-MS spectrum will contain more than one lipid species, albeit with different intensities. Due to the high mass resolution and mass accuracy provided by the Orbitrap this is not an issue, as one still can identify both species and assign their fragments accordingly.

The identity of 76 peaks (listed in Table 2) was confirmed with on tissue tandem-MS (MS2 and MS3 for GD1(d36:1)) of which 60 were unique lipid species and 16 were repeat species detected as [M+H/Na/K]^+^, were detected in both ion modes (e.g., PE(18:0/22:6) as [M ± H]^±^ at *m*/*z* 790.539 and 792.554), or peaks consisting of lipid dimers (e.g., *m*/*z* 1548.194 = PC(18:1/18:0)+PC(16:0/18:1), ≠ CL(78:1)). The mass accuracy (ppm) in full scan (MS1) and tandem-MS data are within ±1 ppm (with lock mass) and ±3 ppm (without lock mass). The *m*/*z* values in Table 2 stem from the tandem-MS spectra, where the lock-mass often was not included. All tandem-MS spectra, with analysis conditions in the scan header plus: identified fragments, mass accuracy and molecular formulas of precursor and fragment ions, are listed in the Supporting Information 2. For simplicity, phospholipid fragments resulting from a headgroup loss (e.g., PS -serine) are referred to as PA(x/x) and fatty acid fragments as FA(x/x). Lipid species and fragments were identified using a combination of the LipostarMSI lipid catalogue with rule-based fragmentation entries, LIPIDMAPS and published literature.^52−60^

### Imaging Single Cells in Tissues with AP-MALDI

Figure 4 shows that AP-MALDI imaging with DAN70 matrix has the potential of imaging individual neuron cells in tissues. PC(18:0/22:6) (Figure 4a,c) shows a unique distribution, localized to small areas in the hippocampus. The associated microscopic image (Figure 4b), taken before matrix application, shows small features corresponding to this distribution, that appear to be single cells. Their location, mainly in the *stratum oriens*, surrounding pyramidal cells (PC(18:1:20:4) blue, Figure 4a), suggests that those are inhibitory neurons/basket cells. This is supported by the resemblance of the PC(18:0/22:6) hippocampus images (Figure 4a,c) to immunohistochemistry images of basket cells^48^ and the PC(18:0/22:6) distribution in the cerebellum, most intense in the region of the Purkinje cells (Figure 4d,e) that are surrounded by basket cells.^49^ This association will have to be confirmed in future experiments.

### Cholesterol Imaging with AP-MALDI

**Figure 5:**
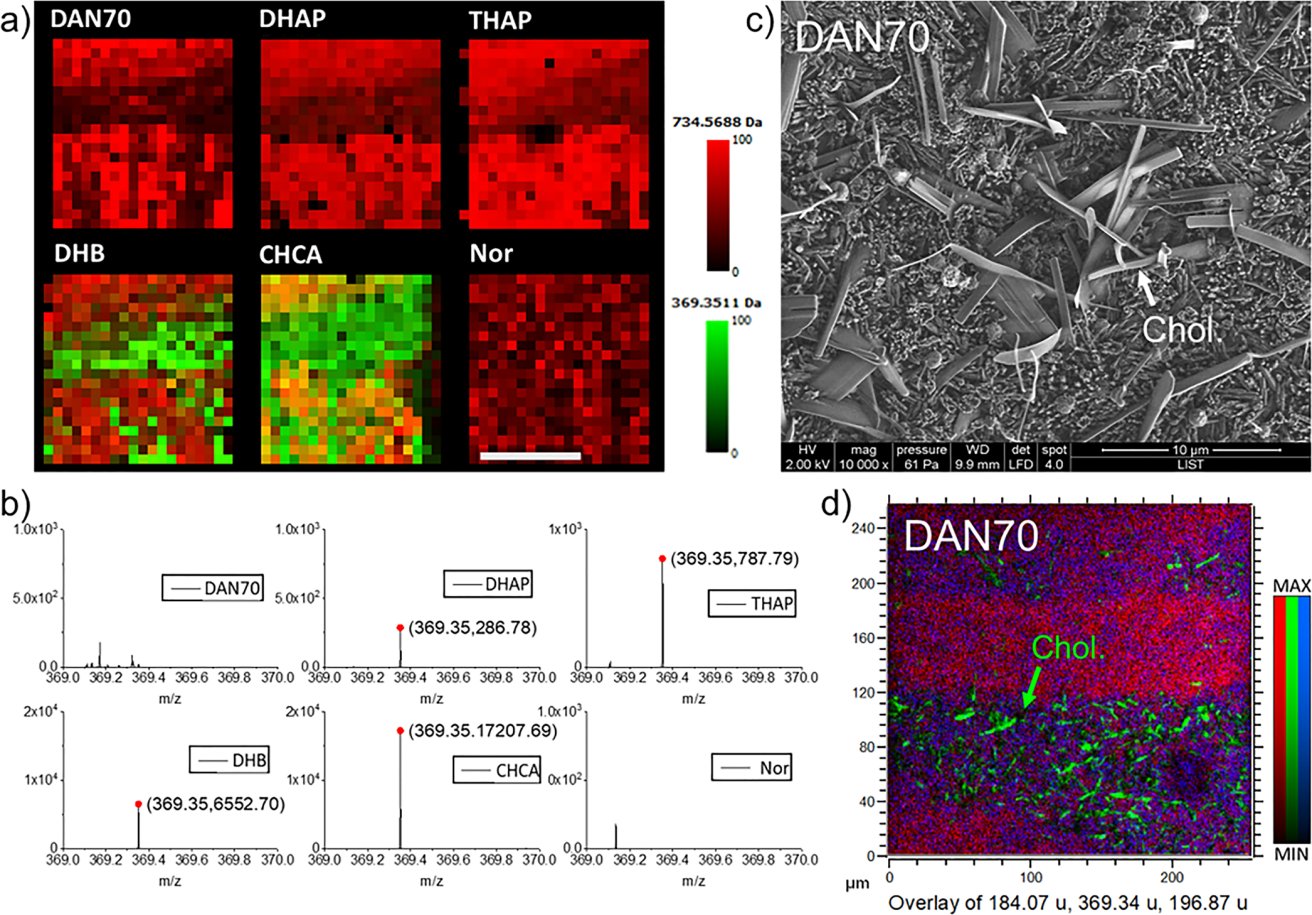
Cholesterol imaging with AP-MALDI, SEM, and ToF-SIMS. (a) AP-MALDI fiber tract/striatum images with various matrices showing cholesterol at *m*/*z* 369.3516 (green) and PC(16:0/16:0) at *m*/*z* 734.5688 (red). Scalebar: 500 μm. (b) Associated mass spectra showing the cholesterol peak and detection levels for each matrix. (c) SEM and (d) ToF-SIMS image of a brain section covered in DAN matrix, in the fiber tract region, cholesterol, *m*/*z* 369.35 (green); PC-headgroup, *m*/*z* 184.07 (red); *m*/*z* 196.87 (blue).

Overall, DAN matrix performed best or well in all experiments we conducted, apart from cholesterol imaging. Figure 5 shows images (Figure 5a) and spectra (Figure 5b) of the striatum/fiber tracts with cholesterol as [M – H_2_O + H]^+^ species (*m*/*z* 369.3516). Cholesterol species [M – H]^+^ at *m*/*z* 385.3464, reported in the literature,^61−63^ was detected at ∼10% of the intensity of *m*/*z* 369.3516. Cholesterol is not detectable with DAN70 and Nor, which produces low intensity signals in general. DHAP and THAP produced relatively low (below 1 × 10^3^ counts) and CHCA and DHB high cholesterol signals (1 × 10^4^ counts and above). While cholesterol detection was vastly different, other lipids were detected at similar levels in the range of 1–4 × 10^4^ (Figure S8, lower for Nor). For DHB, cholesterol-related peaks were the most intense signals in the white matter.

The tendency of cholesterol to migrate to the surface and crystallize during tissue drying has been reported previously.^64,65^ During matrix application, DAN70 does not seem to alter the cholesterol crystals. SEM images of DAN70 on brain sections show regular DAN crystals in the gray matter (Figure S3). In the fiber tract region, additional larger spikelike crystals are visible (Figure 5c). Those crystals resemble cholesterol crystals on dried tissue sections reported in the literature.^65^ Indeed, ToF-SIMS imaging on brain tissue covered with DAN70 matrix confirmed them to be cholesterol crystals (Figure 5d). The addition of 0.1% TFA to DAN70 matrix did not alleviate the issue. No such crystals were found on the surfaces of tissue slices covered with CHCA or DHB, suggesting that these matrices can dissolve and incorporate cholesterol, therefore enabling its detection with MALDI.

## Discussion

We analyzed 11 sample preparation protocols with six different, vacuum stable, and unstable matrices to evaluate their performance for lipid imaging on brain tissue with AP-MALDI-Orbitrap-MSI and on-tissue tandem-MS.

Using strict selection criteria, we were able to assign 155/136 lipid species in AP-MALDI images in positive-/negative-ion mode. These results are comparable to vacuum MALDI-MSI approaches.^66^ AP-MALDI detected lipids with sufficient S/N to perform on-tissue tandem-MS and determine the structure of 76 peaks (60 unique lipids, Table 2) and with the added benefit of less pronounced tissue drying and cracking due to the harsh vacuum environment.^29,32^ Of the studied matrices, DAN was the most versatile matrix (high spatial resolution, high signal intensity and numerous lipids detected in positive+negative ion mode, similar results found for vacuum MALDI),^67^ but other matrices were superior for studying specific molecules (e.g., DHAP for gangliosides, DHB for cholesterol, more species detected in positive ion with THAP/CHCA). DAN protocol alterations such as tissue washing (DANw) and the addition of acids (DANhbfa, DANtfa) did increase performance but not as drastically as changing the solvent proportions, increasing water and decreasing acetonitrile. Using DAN dissolved in 70% acetonitrile and 30% water (DAN70) increased the number of detected lipids more than 2-fold (compared to 90% acetonitrile in DAN90) without decreasing spatial resolution. Further increasing the water content might yield even better results but could make it difficult to dissolve DAN completely. Norharmane could be used in both ion modes as well, but due to low signal intensity (even taking the low noise levels in the spectrum into account), many isotopical peaks fell below the signal-to-noise threshold and therefore fewer peaks were assigned with our selection criteria. Nevertheless, many lipids were present and the full brain analysis with norharmane could be used to guide tandem-MS analysis.

The majority of MALDI-MSI publications state their imaging resolution/pixel size in terms of laser crater size or stage stepping size.^32^ This can be misleading, as it does not accurately reflect the spatial resolution for detecting chemical changes in the sample. In this study, we demonstrated our spatial resolution of 10 μm with on-tissue linescans of lipid species that combine laser focus, stage stepping size (or in our case, raster speed), and crystal size into one metric. A better laser focus is possible, but this would significantly reduce the signal. A value of 10 μm corresponds to the size of one cell and was sufficient to correlate lipid species with single cells/neurons in the hippocampus (Figure 4). It should be noted that even though it is possible to image an entire brain section at 10 μm spatial resolution and at high mass resolution, the resulting data files would be too large to process on most computers. Therefore, reducing the spatial resolution for the analysis of larger areas is advised.

DHB used to be the gold-standard for MALDI analysis, and it is still used in many matrix comparison studies.^38,42^ Here, DHB was outperformed in all aspects by other matrices, other than for cholesterol detection. Cholesterol is of interest to many scientists because of its abundance and its involvement in metabolism and disease.^68,69^ It can be easily detected with ToF-SIMS,^65^ but MALDI usually requires additional steps to enhance cholesterol ionization.^43,70^ The main issue seems to be that cholesterol forms large, solid crystals on the sample surface. Even though they are destroyed by the laser, cholesterol is not ionized sufficiently without being integrated into (and cocrystallized with) the matrix. Therefore, only matrix protocols that can dissolve cholesterol crystals enable its detection with (AP-)MALDI-MSI. The DAN-matrix protocols used here left the cholesterol crystals intact; however, other lipids were still detected in the fiber tracts.

For tandem-MS analysis, THAP and DAN70 worked comparatively well due to their high signal intensities and lipid coverage. For most lipids assigned with LipostarMSI and METASPACE, tandem-MS confirmed their identity in accordance with their possible assignments. Only the assigned cardiolipins detected in positive-ion mode were discovered to be lipid dimers instead. This highlights the importance of tandem-MS analysis, not only for the structural elucidation of the detected species but also for confident assignments.

All data sets included in this study contained hundreds of assigned species with different distributions. This data would have been too vast to include in this manuscript. Therefore, for transparency all data sets were uploaded to METASPACE, where their lipid distributions can be viewed. Additionally, the METASPCE projects, created for this publication, can be expanded upon, as further analyses with novel matrix compounds are performed. Links to all data set: full brain positive/negative: AP_MALDI_Full_Brain; hippocampus positive/negative: AP_MALDI_Hippocampus; cerebellum positive: AP_MALDI_MATRIX_pos; cerebellum negative: AP_MALDI_MATRIX_neg.

## Conclusion

For a long time, lipids have taken a backseat to proteins concerning their importance in disease mechanisms. This was partially due to the inability to track the changes in lipid distributions in heterogeneous samples, changes which can be very subtle in homogenized sample extracts. MSI techniques like AP-MALDI-imaging can capture those changes, but data quality can depend strongly on sample preparation. We tested 11 sample preparation protocols for six matrices and the instrument capabilities of the AP-MALDI-Orbitrap system for lipidomics studies. We defined their characteristics in terms of lipid coverage, signal intensities, high spatial resolution imaging capability and usability for positive and negative ion mode. Every matrix had its advantages and disadvantages and knowing their characteristics is crucial for deciding which one is best suited for the scientific needs of a study. For now, we recommend THAP for on tissue tandem-MS, CHCA or DAN for high spatial resolution imaging in positive-ion mode and DAN for high spatial resolution imaging and tandem-MS in negative-ion mode. For multiple experiments on one sample (positive- and negative-ion mode imaging plus tandem-MS) DAN matrix in a solution with higher water content (as demonstrated here with DAN70 containing 30% H_2_O) is the best suited matrix. The matrix recipes were adapted from previous MALDI/AP-MALDI publications and had all been optimized by their respective users, but they ultimately represented only a small number of options. Therefore, we intend to keep testing matrices and expanding the publicly available data sets in METASPACE. In conclusion, AP-MALDI has shown to be comparable to vacuum MALDI for lipid detection, with the added benefit of less pronounced tissue drying and no requirement for vacuum stable matrices. In addition, the AP-MALDI source in combination with Orbitrap-MS allows for a direct transposition of conventional LC–MS fragmentation parameters for lipids. Therefore, AP-MALDI can be considered cost-effective addition to widely available LC/HRMS instruments and a valuable asset in applied, biomedical research.

## Supporting Information Available

Additional AP-MALDI images for all data sets included in this study, SEM images of DAN70 and CHCA matrices, mass spectra for cerebellum analysis with all matrices in positive-/negative-ion mode and signal-to-noise levels for selected species, PCA loadings plots, and images and spectra of the small area-striatum analyses (PDF)
Tandem-MS data for 76 peaks with assigned fragments (PDF)
