# Supplementary material for: Evaluation of 6 MALDI-Matrices for 10 μm Lipid Imaging and On-Tissue MSn with AP-MALDI-Orbitrap
Source: J Am Soc Mass Spectrom. 2022 Mar 31;33(5):760–71. doi: 10.1021/jasms.1c00327 (PMC9074099; doi:10.1021/jasms.1c00327)
Supplement: Supplementary file 1 — js1c00327_si_001.pdf [file js1c00327_si_001.pdf]

Evaluation of 6 MALDI-Matrices for 10 µm lipid imaging and on-tissue MSn with AP-MALDI-Orbitrap

Tina B. Angerer[1*], Jerome Bour[1], Jean-Luc Biagi[1], Eugene Moskovets[2], Gilles Frache[1*]

1 Luxembourg Institute of Science and Technology (LIST), Advanced Characterization platform, Materials Research and Technology, 41, rue du Brill, L-4422 Belvaux, Luxembourg
2 MassTech, Inc., Columbia, Maryland 21046, United States

*Corresponding Authors: tina.angerer@list.lu, gilles.frache@list.lu

Table of contents

Figure S 1 All AP-MALDI cerebellum datasets in positive (top) and negative (bottom) ion mode........2

Figure S 2 All AP-MALDI hippocampus datasets in positive (top) and negative (bottom) ion mode. ...3

Figure S 3 SEM images...4

Figure S 4 Cerebellum spectra of the lipid region for all matrices studied in pos. ion mode. 4

Figure S 5 Cerebellum spectra of the lipid region for all matrices studied in neg. ion mode. 5

Figure S 6 Signal to noise ratios (S/N) for various signals in positive and negative ion mode.............5

Figure S 7 PCA loadings plots for cerebellum spectra analysis in positive and negative ion mode......6

Figure S 8 Small area fibre/striatum tract images with various matrices. 6

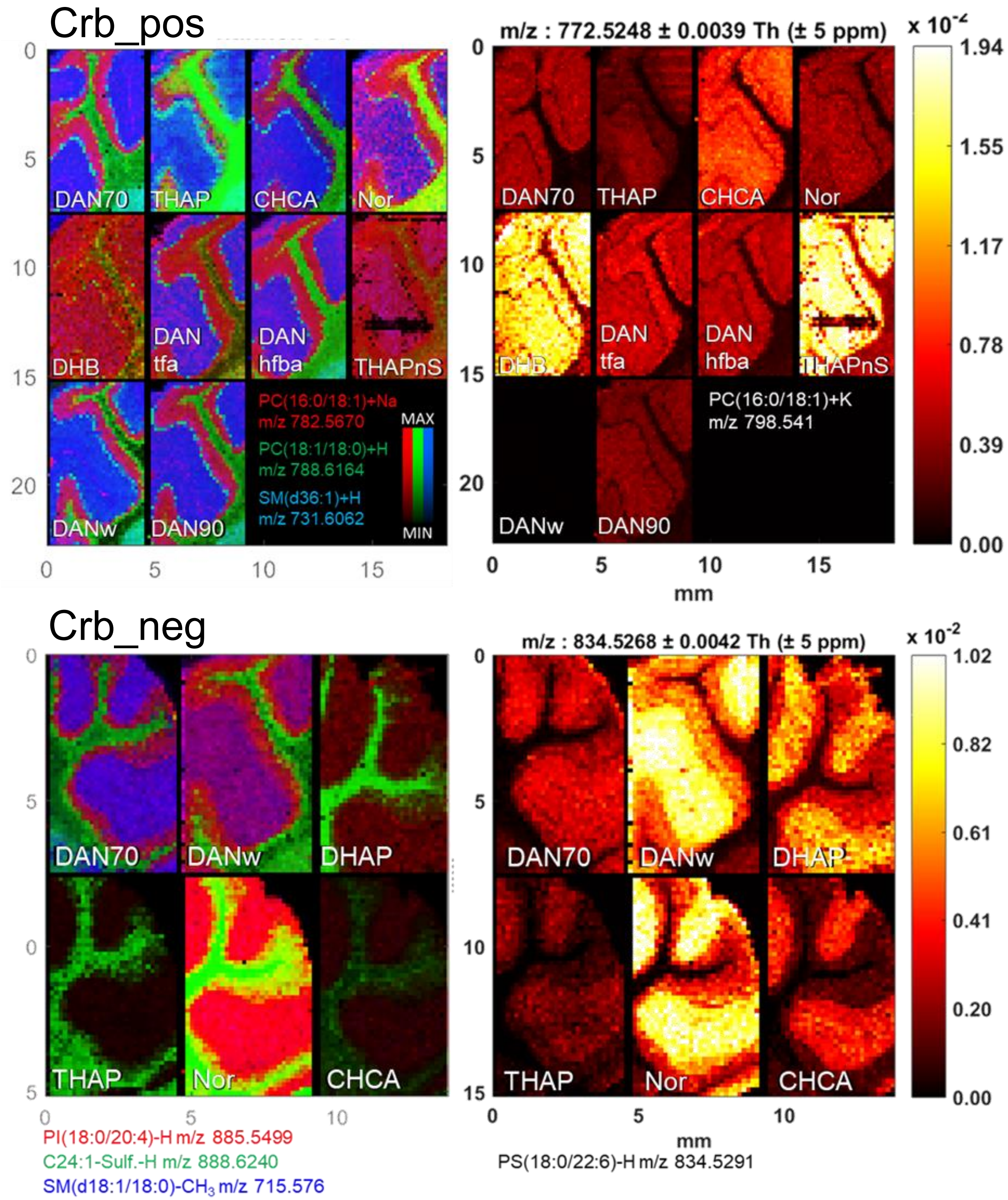

Figure S 1 All AP-MALDI cerebellum datasets in positive (top) and negative (bottom) ion mode. Matrix stated in left, bottom corner of each image. Displayed lipid species stated underneath each image group for RGB and single ion images.

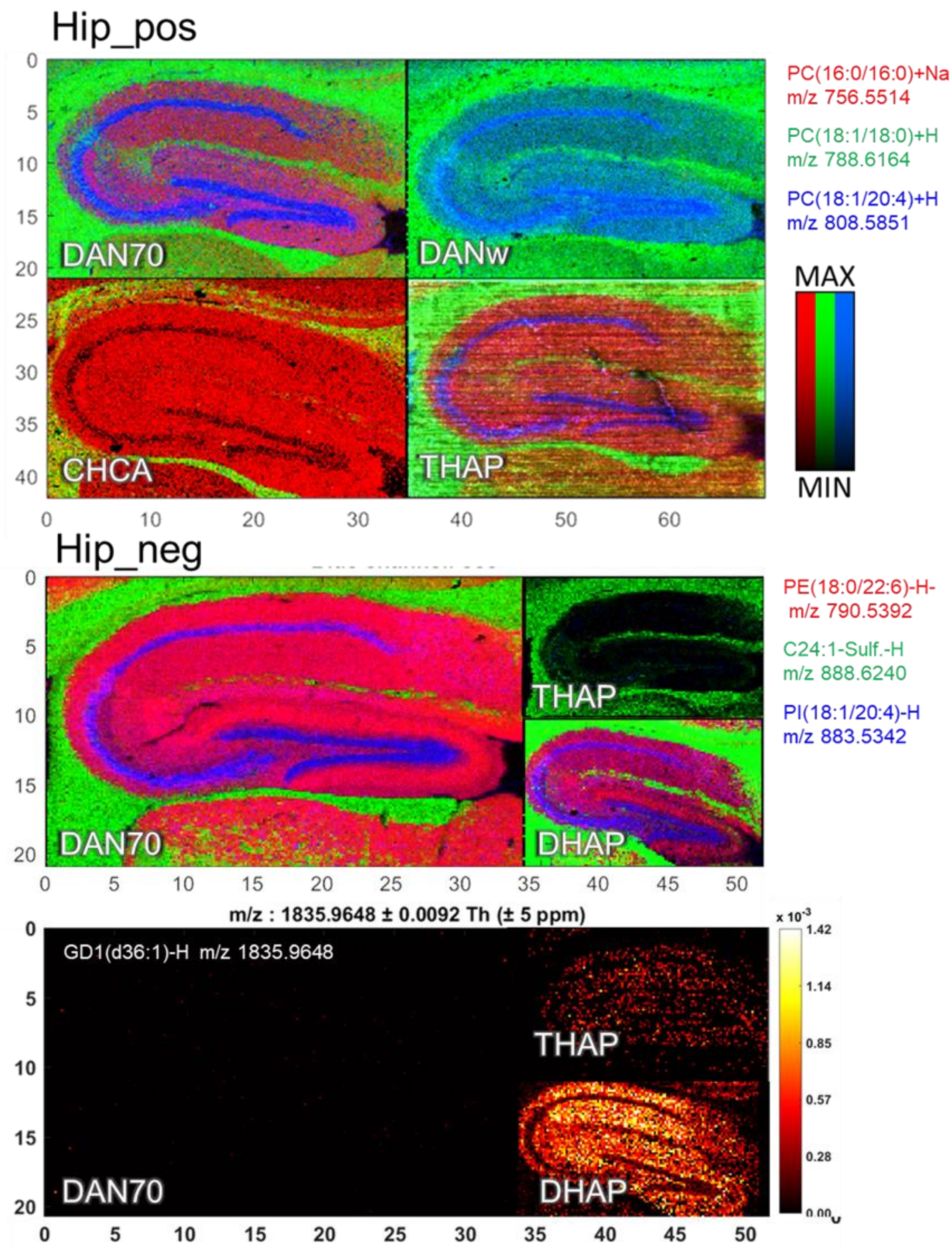

Figure S 2 All AP-MALDI hippocampus datasets in positive (top) and negative (bottom) ion mode. Matrix stated in left, bottom corner of each image. Displayed lipid species stated next to each image group for RGB and single ion images.

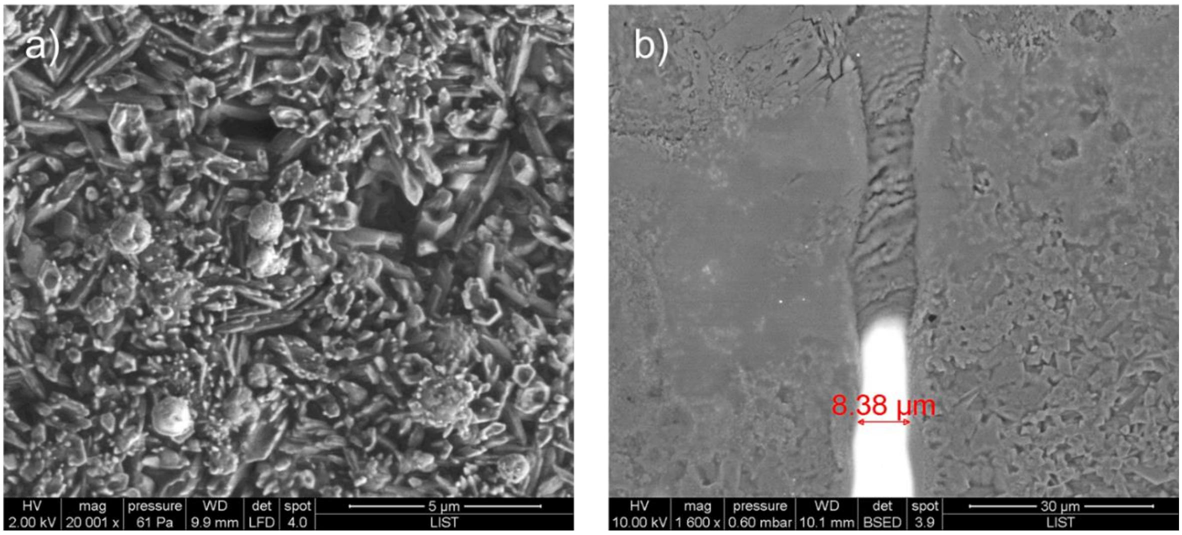

Figure S 3 SEM images of a) DAN matrix crystals on tissue and b) a laser track through CHCA matrix on ITO glass.

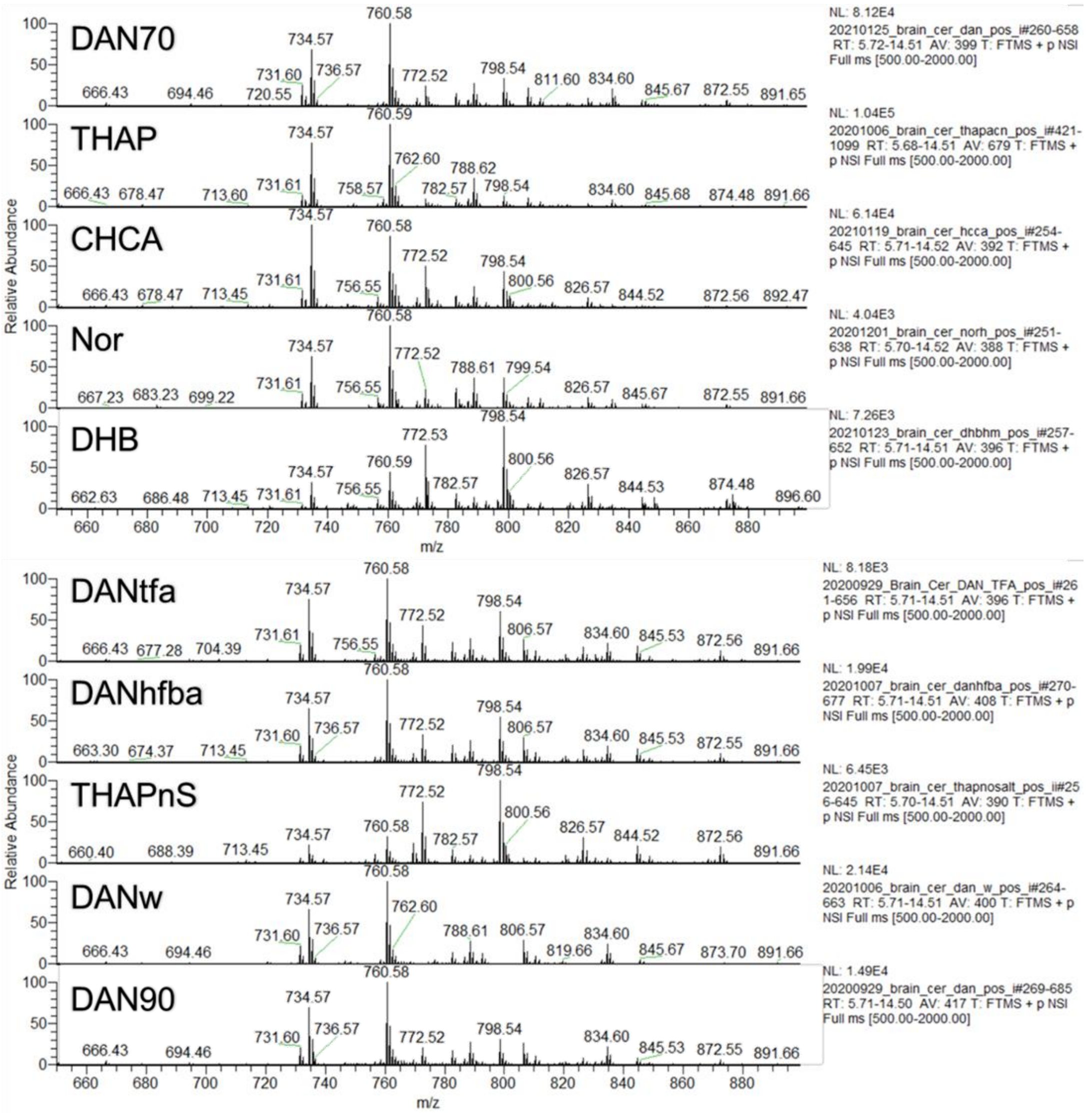

Figure S 4 Cerebellum spectra of the lipid region for all matrices studied in pos. ion mode.

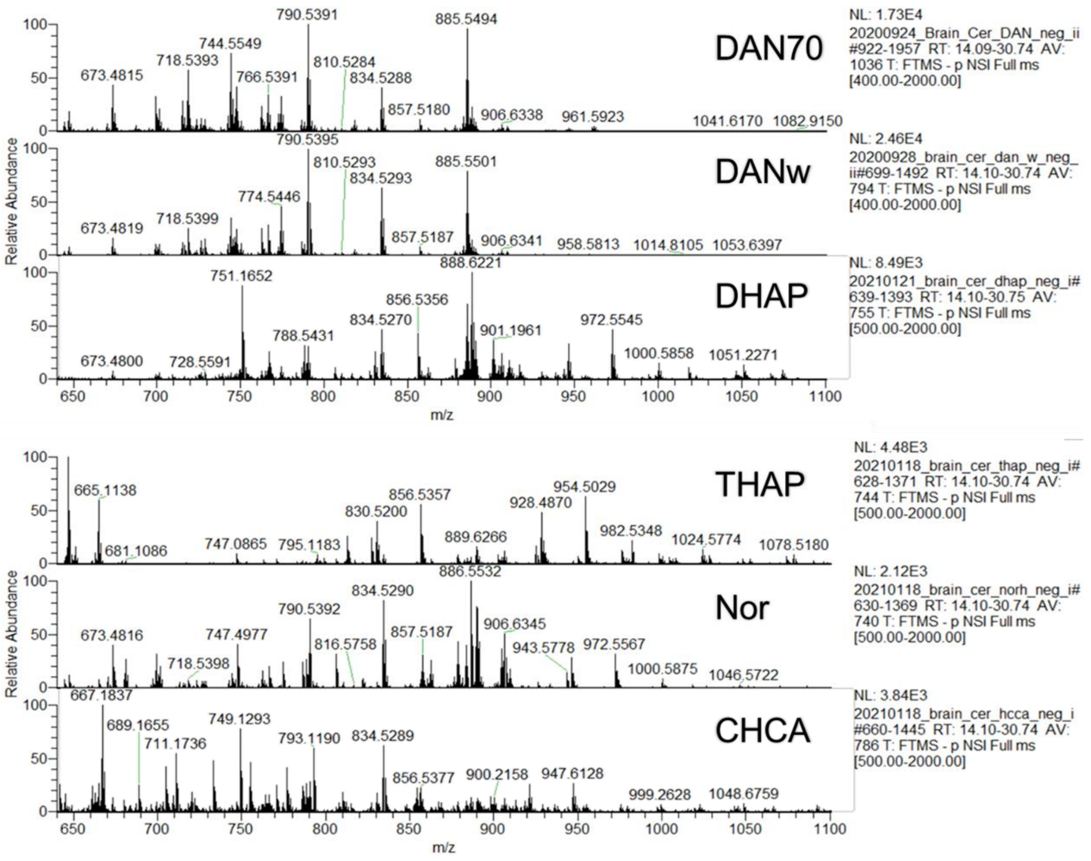

Figure S 5 Cerebellum spectra of the lipid region for all matrices studied in neg. ion mode.

POS	SM(d36:1)+H m/z 731.60		
Matrix	S	N	S/N
DAN70	2.05E+04	11.33	1809
THAP	1.43E+04	12.56	1139
CHCA	1.32E+04	14.89	887
Nor	7.20E+02	2.05	351
DHB	3.91E+02	8.39	47
DANtfa	1.61E+03	2.02	797
DANhfba	3.91E+03	2.43	1609
THAPnS	4.32E+02	1.56	277
DANw	4.67E+03	2.21	2113
DAN90	3.18E+03	2.46	1293

POS	PC(16:0/18:1)+H m/z 760.58		
Matrix	S	N	S/N
DAN70	8.12E+04	11.75	6911
THAP	1.04E+05	13.01	7994
CHCA	5.35E+04	16.34	3274
Nor	4.04E+03	2.1	1924
DHB	3.28E+03	8.98	365
DANtfa	8.18E+03	2.1	3895
DANhfba	1.99E+04	2.53	7866
THAPnS	2.06E+03	1.6	1288
DANw	2.14E+04	2.27	9427
DAN90	1.49E+04	2.56	5820

POS	PC(16:0/18:1)+K m/z 798.54		
Matrix	S	N	S/N
DAN70	2.75E+04	12.29	2238
THAP	1.31E+04	13.61	963
CHCA	2.66E+04	17.77	1497
Nor	1.49E+03	2.16	690
DHB	7.28E+03	9.66	754
DANtfa	4.99E+03	2.19	2279
DANhfba	1.10E+04	2.66	4135
THAPnS	6.45E+03	1.65	3909
DANw	8.17E+01	2.34	35
DAN90	4.67E+02	2.68	174

NEG	PE(18:0/22:6)-H m/z 790.54		
Matrix	S	N	S/N
DAN70	1.81E+04	8.96	2020
DANw	1.33E+04	2.83	4700
DHAP	2.49E+03	7.81	319
THAP	7.45E+01	5.97	12
Nor	1.33E+03	5.76	231
CHCA	1.01E+03	6.94	146

NEG	PI(18:0/20:4)-H m/z 885.55		
Matrix	S	N	S/N
DAN70	2.21E+04	14.18	1559
DANw	1.25E+04	4.39	2847
DHAP	5.94E+03	11.41	521
THAP	n.d.	n.d.	
Nor	2.28E+01	9.19	2
CHCA	6.26E+01	11.34	6

NEG	C24:1-Sulf-H m/z 888.62		
Matrix	S	N	S/N
DAN70	4.26E+04	9.95	4281
DANw	3.21E+03	3.47	925
DHAP	4.94E+03	10.72	461
THAP	1.92E+01	8.44	2
Nor	1.07E+01	8.22	1
CHCA	1.03E+01	10.59	1

Figure S 6 Signal to noise ratios (S/N) for various signals in positive and negative ion mode.

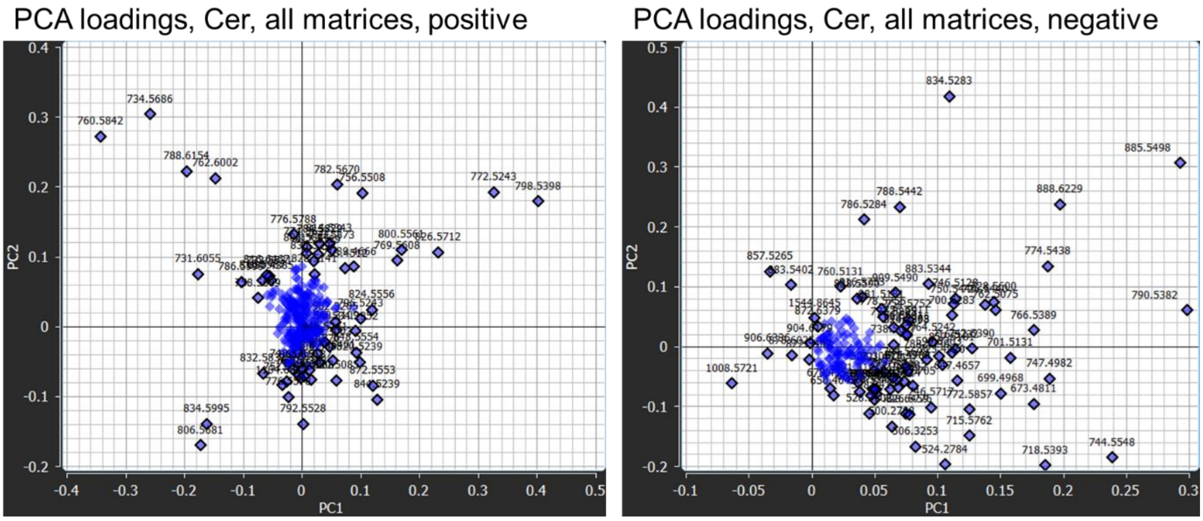

Figure S 7 PCA loadings plots for cerebellum spectra analysis in positive and negative ion mode.

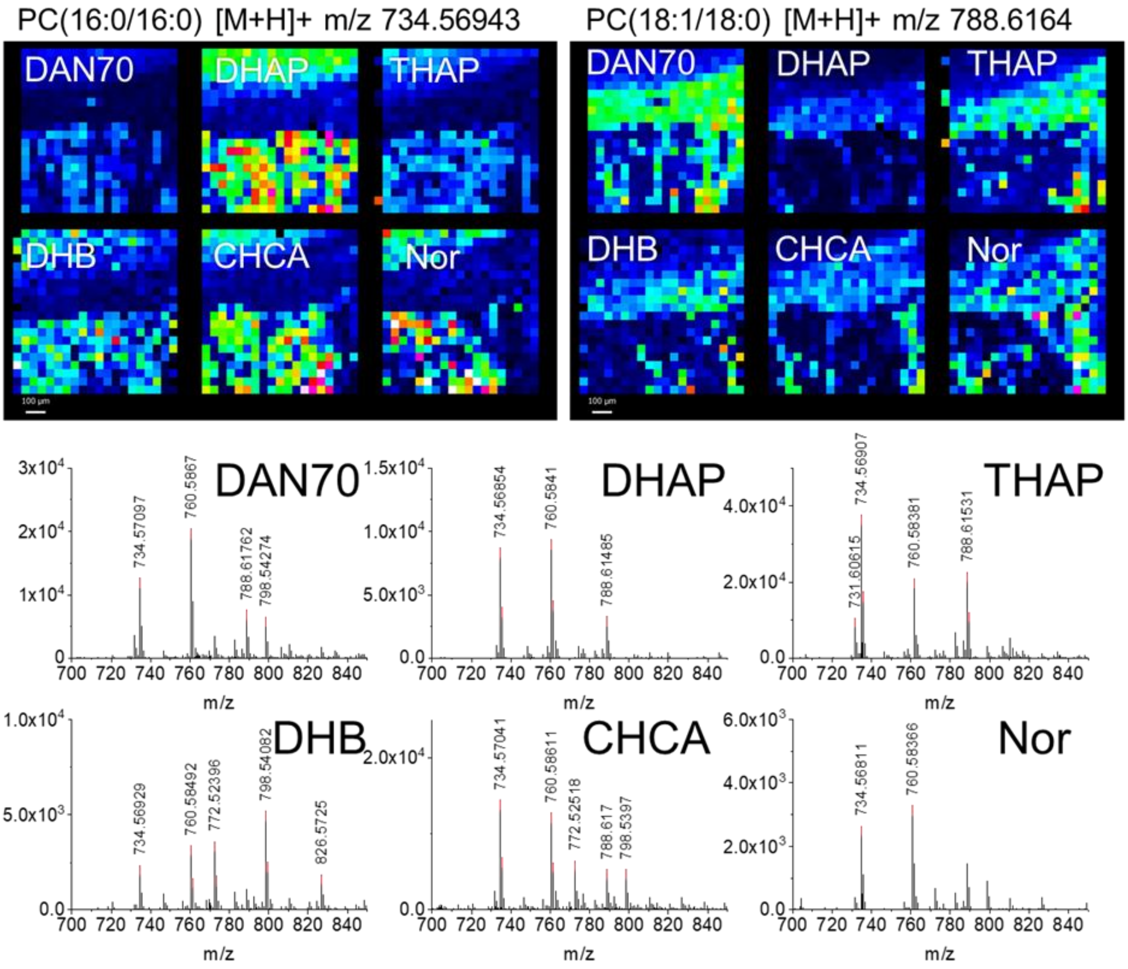

Figure S 8 Small area fibre/striatum tract images with various matrices. Distribution of PC(16:0/16:0) in the grey matter, PC(18:1/18:0) in the white matter and spectra of the lipid region.
