# Supplementary material for: Evaluation of 6 MALDI-Matrices for 10 μm Lipid Imaging and On-Tissue MSn with AP-MALDI-Orbitrap
Source: J Am Soc Mass Spectrom. 2022 Mar 31;33(5):760–71. doi: 10.1021/jasms.1c00327 (PMC9074099; doi:10.1021/jasms.1c00327)
Supplement: Supplementary file 2 — js1c00327_si_002.pdf [file js1c00327_si_002.pdf]

Evaluation of 6 MALDI-Matrices for 10 µm lipid imaging and on-tissue MSn with AP-MALDI-Orbitrap

Tina B. Angerer[1*], Jerome Bour[1], Jean-Luc Biagi[1], Eugene Moskovets[2], Gilles Frache[1*]

1 Luxembourg Institute of Science and Technology (LIST), Advanced Characterization platform, Materials Research and Technology, 41, rue du Brill, L-4422 Belvaux, Luxembourg
2 MassTech, Inc., Columbia, Maryland 21046, United States

*Corresponding Authors: tina.angerer@list.lu, gilles.frache@list.lu

Table of Contents

Positive ion mode MS/MS, Identified species 1
PC(16:0/9:0(OH)) [M+H]+ m/z 650.4392 1
PC(16:0/9:0(COOH)) [M+H]+ m/z 666.4341 2
PC(18:0/9:0(OH)) [M+H]+ m/z 678.4705 3
PC(16:0/16:0)-TMA [M+K]+ m/z 713.4518 4
PDME(16:0/16:0) [M+H]+ m/z 720.5538 5
SM(d36:1) [M+H]+ m/z 731.6062 6
PC(16:0/16:0) [M+H]+ m/z 734.56943 7
PC(16:0/16:0) [M+Na]+ m/z 756.5514 8
PC(16:0/18:1) [M+H]+ m/z 760.5851 9
PC(16:0/16:0)* [M+K]+ m/z 772.5253 10
PC(16:0/18:1)* [M+Na]+ m/z 782.5670 11
HexCer(d18:1/22:1) [M+H]+ m/z 782.6505 12
SM(d40:2) [M+H]+ m/z 785.6531 13
PC(18:1/18:1) [M+H]+ m/z 786.6007 14
PC(18:1/18:0) [M+H]+ m/z 788.6164 15
PE(40:7)* [M+H]+ m/z 790.5381 16
PE(18:0/22:6) [M+H]+ m/z 792.5538 17
PC(16:0/18:1)* [M+K]+ m/z 798.541 18
PC(16:0/22:6) [M+H]+ m/z 806.5694 19
PE(38:4) [M+K]+ m/z 806.5097 20
PC(18:1/20:4) [M+H]+ m/z 808.5851 21
HexCer(d18:1/24:2) [M+H]+ m/z 808.6661 22
SM(d42:2) [M+H]+ m/z 813.6844 23
PC(18:1/18:0)* [M+K]+ m/z 826.5723 24
PC(18:0/22:6) [M+H]+ m/z 834.6007 25
PC(38:6) [M+K]+ m/z 844.5253 26
PC(38:4) [M+K]+ m/z 848.5566 27
HexCer(d18:1/24:1(2OH)) [M+Na]+ m/z 848.6586 28
PC(40:6) [M+K]+ m/z 872.5566 29
PC(16:0/18:1)+PC(16:0/16:0) [M+M+H]+ m/z 1494.147 30
PC(16:0/18:1) [2M+H]+ m/z 1520.163 31
PC(18:1/18:0)+PC(16:0/18:1) [M+M+H]+ m/z 1548.194 32
Negative ion mode MS/MS, Identified species 33
Cer(d18:0/18:1) [M-H]- m/z 564.535 33

CerP(d18:1/18:0) [M-H]- m/z 644.5025 ... 34
PA(16:0/16:1) [M-H]- m/z 645.4501 ... 35
PA(16:0/16:0) [M-H]- m/z 647.4657 ... 36
CerP(d38:2) [M-H]- m/z 670.5181 ... 37
PA(16:0/18:1) [M-H]- m/z 673.4814 ... 38
SM(d34:1) [M-CH3]- m/z 687.5447 ... 39
SM(d18:1/18:0) [M-CH3]- m/z 715.576 ... 40
PE(16:0/18:0) [M-H]- m/z 718.5392 ... 41
PE(P-18:1/18:1) [M-H]- m/z 726.5443 ... 42
PE(P-18:0/18:1) [M-H]- m/z 728.56 ... 43
PE(18:1/18:1) [M-H]- m/z 742.5392 ... 44
PC(16:0/18:1) [M-CH3]- m/z 744.5549 ... 45
PA(18:0/22:6) [M-H]- m/z 747.497 ... 46
PE(P-18:1/20:1) [M-H]- m/z 754.5756 ... 47
PE(18:0/20:4) [M-H]- m/z 766.5392 ... 48
SM(d18:1/22:0) [M-CH_3]- m/z 771.6386 ... 49
PE(P-18:0/22:6) -H [M-H]- m/z 774.5443 ... 50
PE(18:0/20:4(OH)) [M-H]- m/z 782.5341 ... 51
PC(16:1/22:6) [M-CH_3]- m/z 788.5236 ... 52
PS(18:1/18:0) [M-H]- m/z 788.5447 ... 52
PE(18:0/22:6) [M-H]- m/z 790.5392 ... 53
HexCer(d18:1/22:0(2OH)) [M-H]- m/z 798.6465 ... 54
C18:1-Sulf [M-H]- m/z 806.5458 ... 55
C18(OH)-Sulf [M-H]- m/z 822.5407 ... 56
HexCer(d18:1/24:0(2OH)) [M-H]- m/z 826.6778 ... 57
PS(18:0/22:6) [M-H]- m/z 834.5291 ... 58
PS(18:1/22:0) [M-H]- m/z 844.6073 ... 59
PI(16:0/20:4) [M-H]- m/z 857.5186 ... 60
C22-Sulf. [M-H]- m/z 862.6084 ... 61
PI(18:0/18:1) [M-H]- m/z 863.5655 ... 62
PS(18:1/24:0) [M-H]- m/z 872.6386 ... 63
C22(OH)-Sulf [M-H]- m/z 878.6033 ... 64
PI(18:1/20:4) [M-H]- m/z 883.5342 ... 65
PI(18:0/20:4) [M-H]- m/z 885.5499 ... 66
C24:1-Sulf. [M-H]- m/z 888.624 ... 67
C24-Sulf. [M-H]- m/z 890.6397 ... 68
PI(18:0/20:4(OH)) [M-H]- m/z 901.5448 ... 69
C24(OH)-Sulf. [M-H]- m/z 906.6346 ... 70
Gal-GalNAc-Gal-Glc-(d36:1) [M-H]- m/z 1254.777 ... 71
GM1(d36:1) [M-H]- m/z 1544.869 ... 72
GM1(d38:1) [M-H]- m/z 1573.904 ... 73
GD1(d36:1) [M-H]- m/z 1835.9648 ... 74
GD1(d36:1) [M-2H+K]- m/z 1873.921 ... 75

Positive ion mode MS/MS, Identified species

PC(16:0/9:0(OH)) [M+H]+ m/z 650.4392

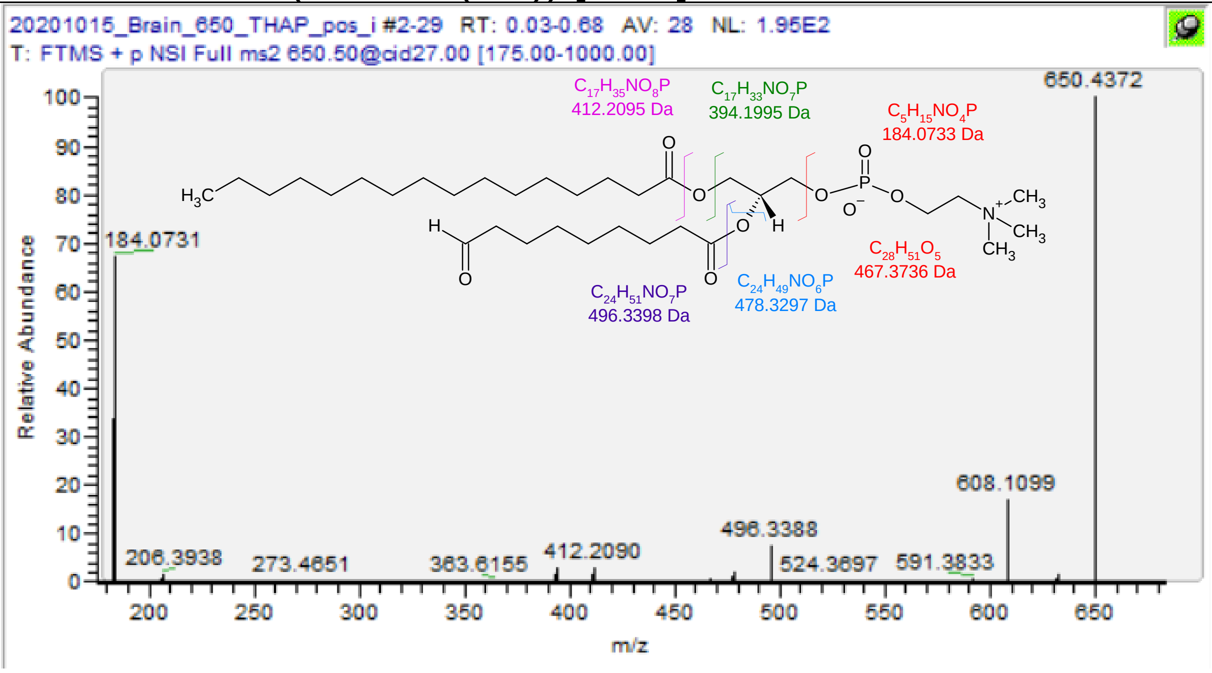

m/z	[I]	Species	Formula	Da	ppm
184.0731	69.03	PC-Hg	C5H15O4NP	184.0733	1.20
394.1983	2.52	PC(9:0(OH)/-) -H2O	C17H33O7NP	394.1989	1.57
412.2090	3.22	PC(9:0(OH)/-)	C17H35O8NP	412.2095	1.16
467.3718	0.58	PC -Hg	C28H51O5	467.3731	2.78
478.3282	1.82	PC(16:0/-) -H2O	C24H49O6NP	478.3292	2.09
496.3388	7.52	PC(16:0/-)	C24H51O7NP	496.3398	1.95
**650.4372**	**100**	**PC(16:0/9:0(OH))**	**C33H65O9NP**	**650.4392**	**3.00**

PC(16:0/9:0(COOH)) [M+H]+ m/z 666.4341

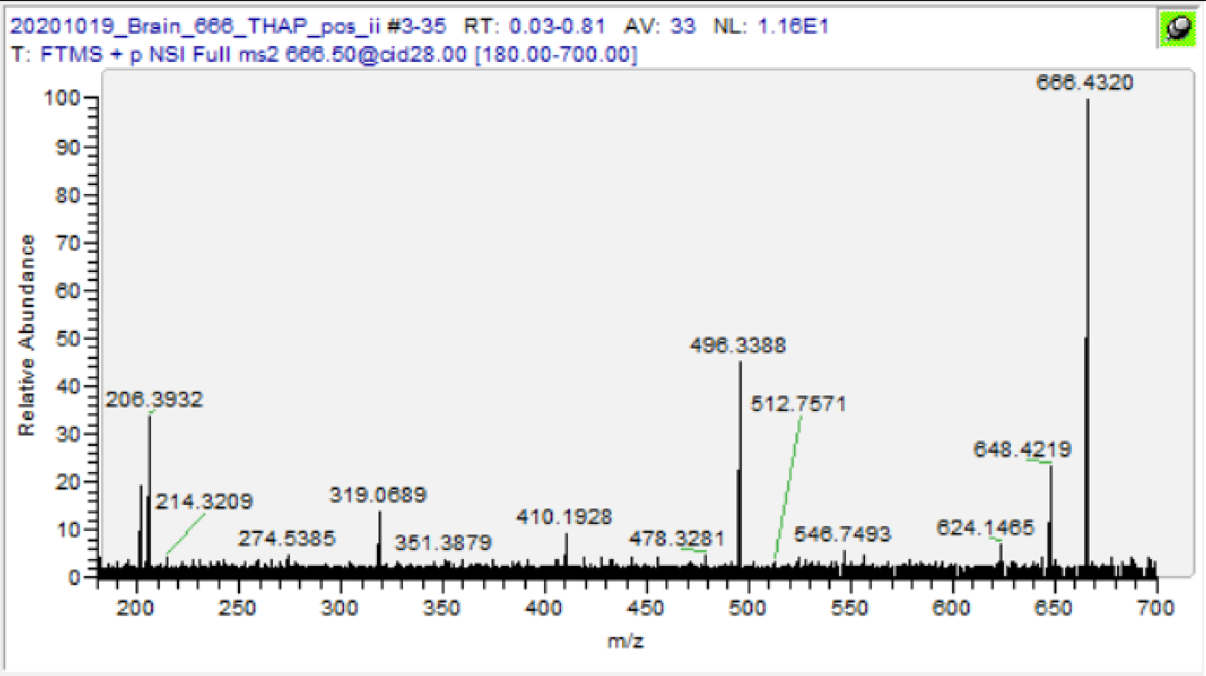

m/z	[I]	Species	Formula	Da	ppm
410.1930	8.19	PC(9:0(COOH)/-) -H2O	C17H33O8NP	410.1938	2.02
428.2039	4.77	PC(9:0(COOH)/-)	C17H35O9NP	428.2044	1.14
478.3281	5.67	PC(16:0/-) -H2O	C24H49O6NP	478.3292	2.30
496.3388	52.22	PC(16:0/-)	C24H51O7NP	496.3398	1.95
648.4218	20.89	PC -H2O	C33H63O9NP	648.4235	2.62
**666.4321**	**100**	**PC(16:0/9:0(COOH))**	**C33H65O10NP**	**666.4341**	**2.94**

PC(18:0/9:0(OH)) [M+H]+ m/z 678.4705

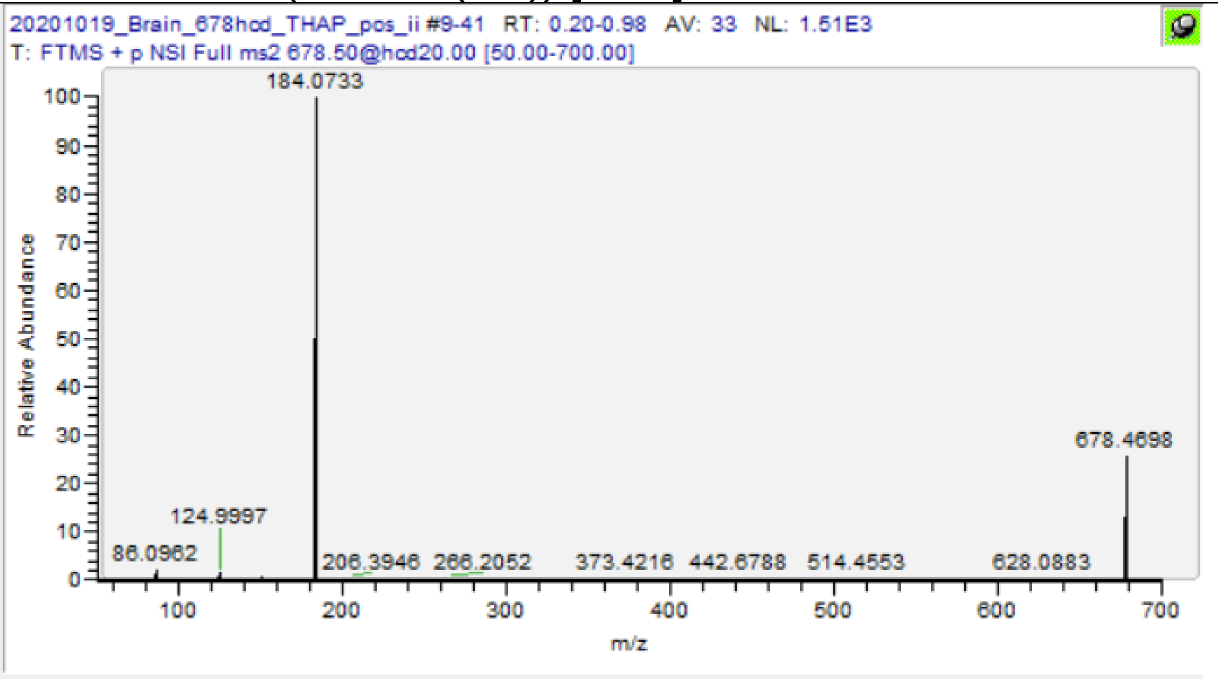

m/z	[I]	Species	Formula	Da	ppm
86.0962	2.41	PC-Frag	C5H12N	86.09643	2.67
124.9997	1.86	PC-Frag	C2H6O4P	124.9998	0.96
184.0733	100	PC-Hg	C5H15O4NP	184.0733	0.11
**678.4698**	**23.73**	**PC(18:0/9:0(OH))**	**C35H69O9NP**	**678.4705**	**0.96**

PC(16:0/16:0)-TMA [M+K]+ m/z 713.4518

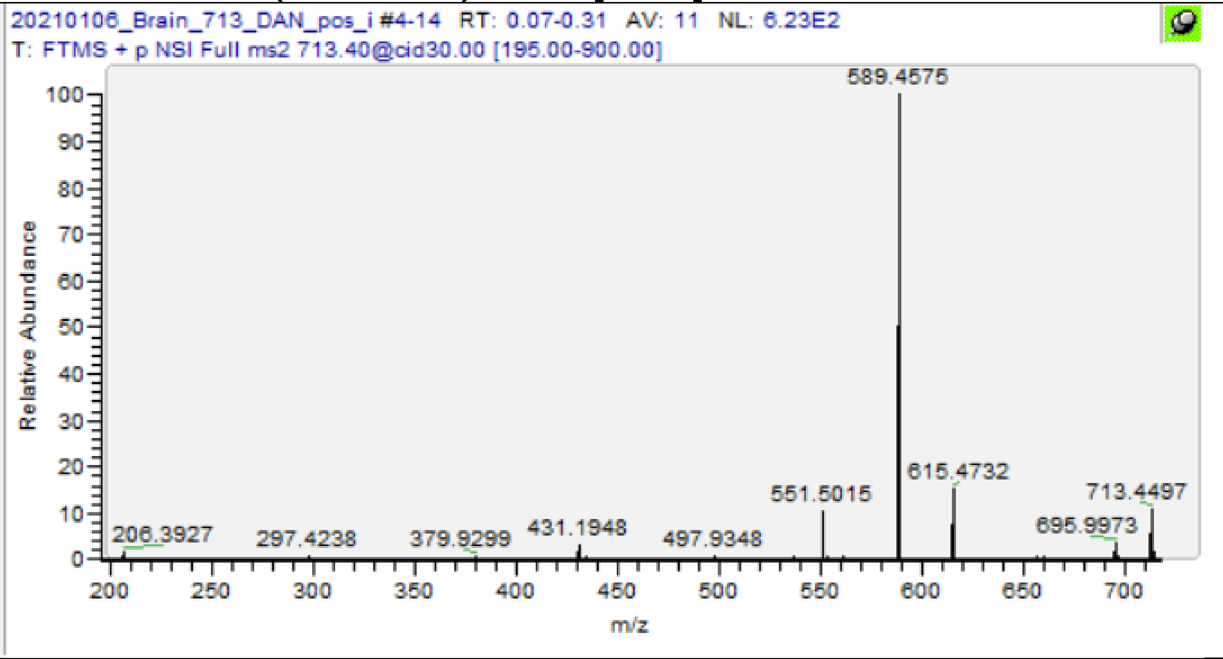

m/z	[I]	Species	Formula	Da	ppm
162.9557	100	PC-Frag +K	C2H5O4PK	162.9557	0
431.1948	3.04	PA(16:0) +K	C19H37O6PK	431.1959	2.66
551.5015	10.16	PL(16:0/16:0) -Hg	C35H67O4	551.5034	3.42
589.4575	100	PL(16:0/16:0) -Hg +K	C35H66O4K	589.4593	3.00
**713.4497**	**10.79**	**PC(16:0/16:0)-TMA +K**	**C37H71O8PK**	**713.4518**	**2.95**

PDME(16:0/16:0) [M+H]+ m/z 720.5538

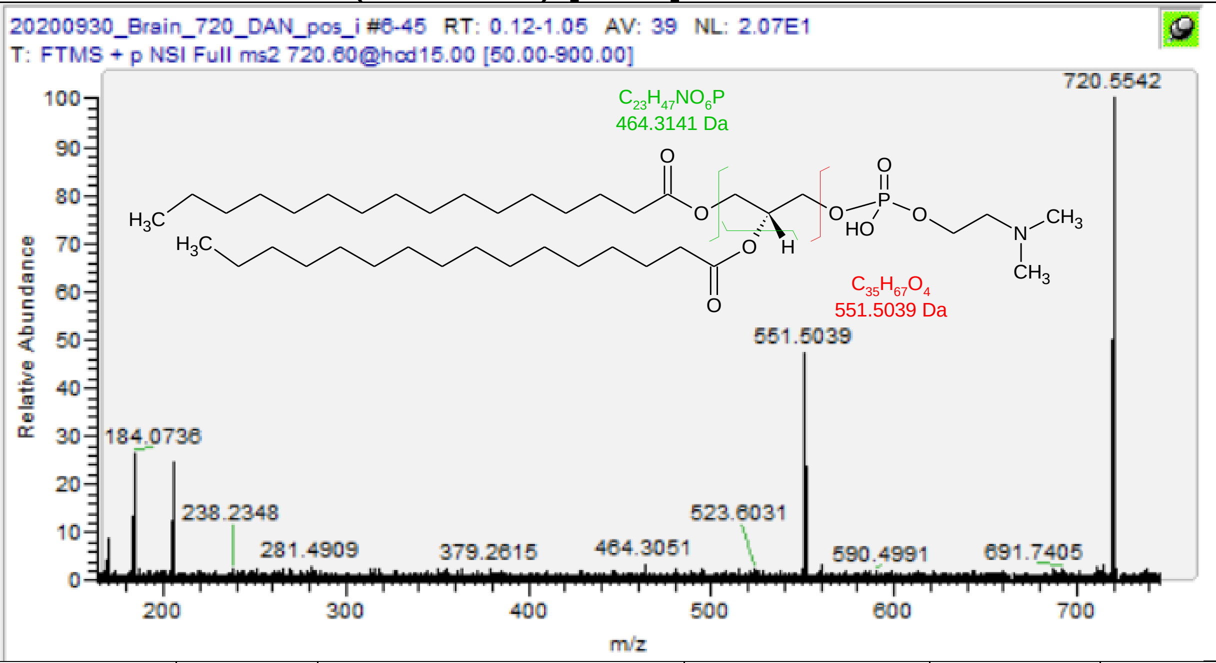

m/z	[I]	Species	Formula	Da	ppm
170.0579	11.96	PDME-Hg	C4H13O4NP	170.0577	-1.35
464.3059	3.05	PDME(16:0/-)	C23H47O6NP	464.3136	16.48
551.5038	55.43	PDME(16:0/16:0) -Hg	C35H67O4	551.5034	-0.74
**720.5543**	**100**	**PDME(16:0/16:0)**	**C39H79O8NP**	**720.5538**	**-0.72**
184.0736	28.46	PC-Hg	C5H15O4NP	184.0733	-1.52
720.5913	49.88	PC(O-32:0)	C40H83O7NP		-1.57

SM(d36:1) [M+H]+ m/z 731.6062

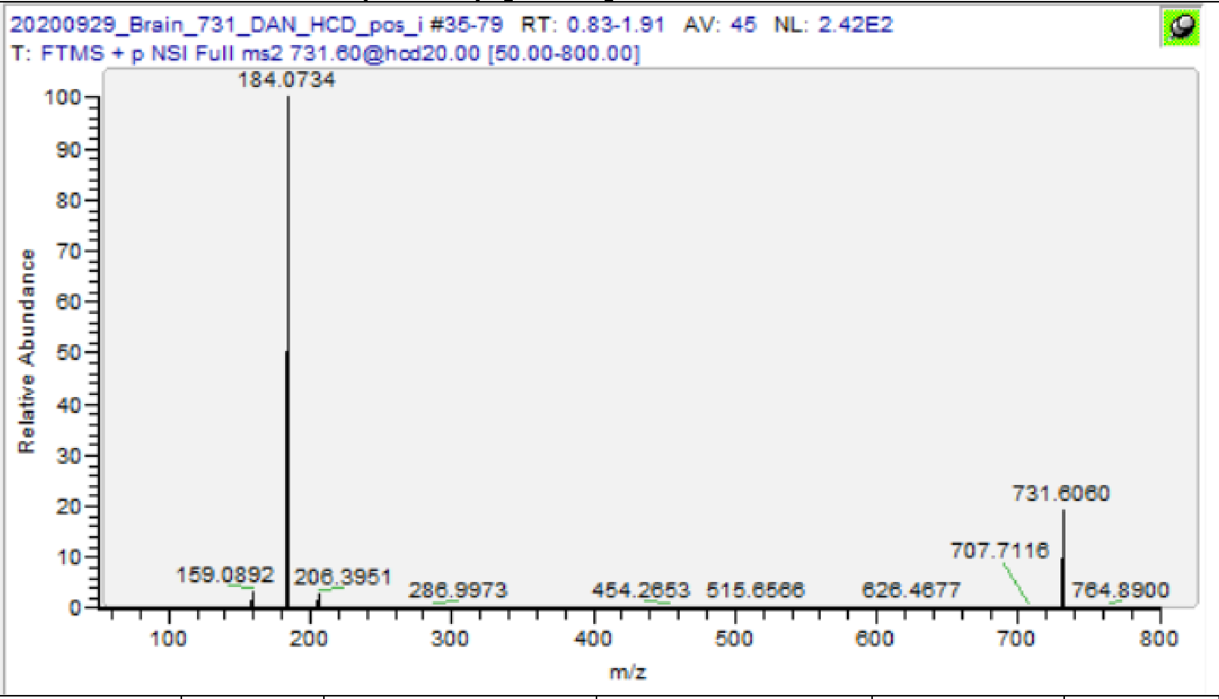

m/z	[I]	Species	Formula	Da	ppm
184.0734	100	SM-Hg	C5H15O4NP	184.0733	-0.43
713.5938	24.68	SM(d36:1) -H2O	C41H82O5N2P	713.5956	2.51
**731.6060**	**27.2**	**SM(d36:1)**	**C41H84O6N2P**	**731.6062**	**0.21**

PC(16:0/16:0) [M+H]+ m/z 734.56943

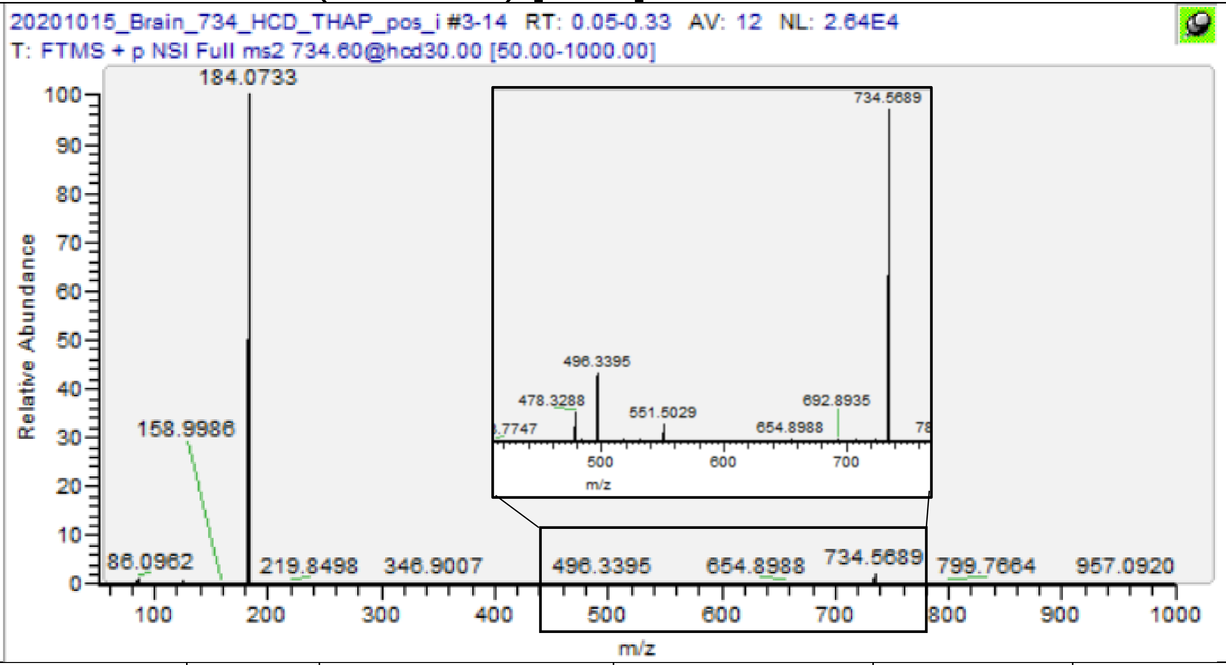

m/z	[I]	Species	Formula	Da	ppm
86.0962	0.88	PC-Frag	C5H12N	86.09643	2.67
124.9997	0.53	PC-Frag	C2H6O4P	124.9998	0.96
184.0732	100	PC-Hg	C5H15O4NP	184.0733	0.65
478.3289	0.18	PC(16:0/-) -H2O	C24H49O6NP	478.3292	0.63
496.3394	0.46	PC(16:0/-)	C24H51O7NP	496.3398	0.75
551.5029	0.11	PL(16:0/16:0) -Hg	C35H67O4	551.5034	0.89
**734.5689**	**2.31**	**PC(16:0/16:0)**	**C40H81O8NP**	**734.56943**	**0.72**

PC(16:0/16:0) [M+Na]+ m/z 756.5514

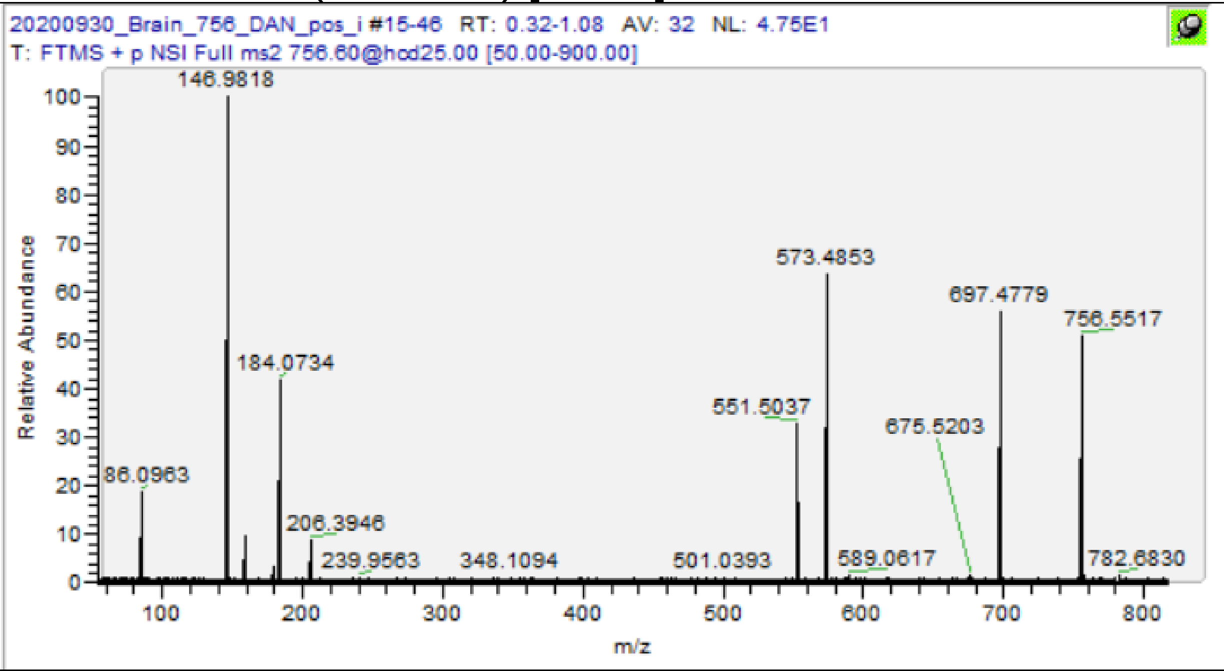

m/z	[I]	Species	Formula	Da	ppm
86.0962	16.11	PC-Frag	C5H12N	86.09643	2.67
146.9818	100	PC-Frag+Na	C2H5O4PNa	146.9818	-0.20
184.0734	60.35	PC-Hg	C5H15O4NP	184.0733	-0.43
551.5042	31.31	PL(16:0/16:0) -Hg	C35H67O4	551.5034	-1.47
573.4849	84.93	PL(16:0/16:0) -Hg +Na	C35H66O4Na	573.4853	0.75
697.4789	54.74	PC(16:0/16:0)-TMA +Na	C37H71O8PNa	697.4779	-1.46
**756.5530**	**47.36**	**PC(16:0/16:0)+Na**	**C40H80O8NPNa**	**756.5514**	**-2.14**

PC(16:0/18:1) [M+H]+ m/z 760.5851

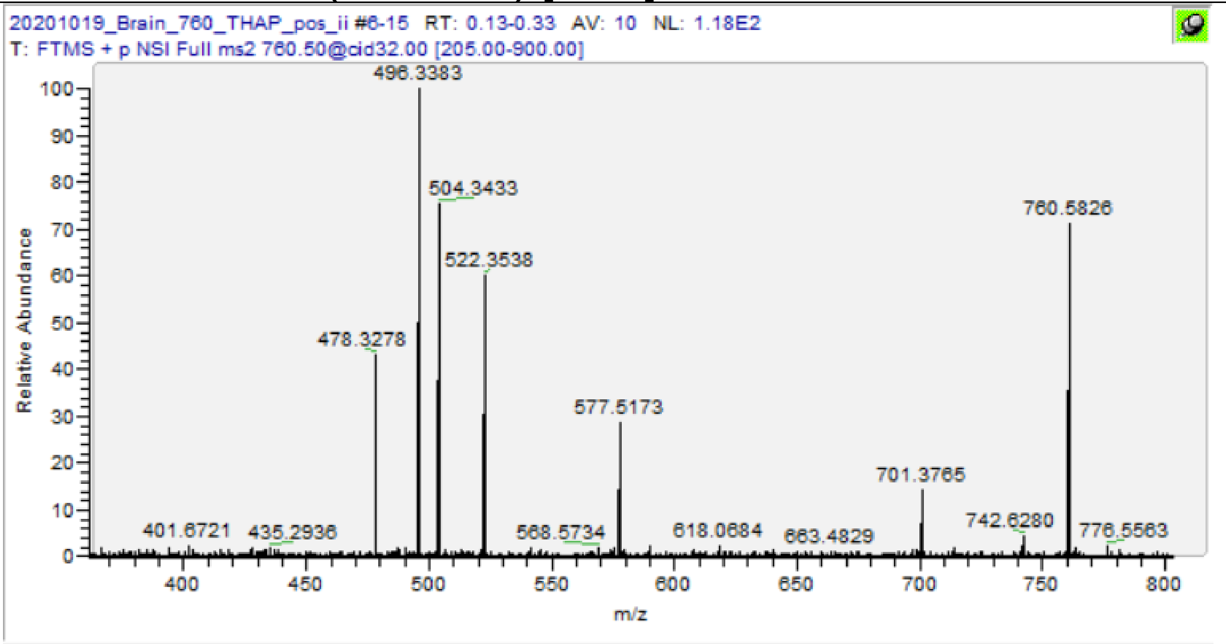

m/z	[I]	Species	Formula	Da	ppm
478.3279	45.02	PC(16:0/-) -H2O	C24H49O6NP	478.3292	2.72
496.3383	100	PC(16:0/-)	C24H51O7NP	496.3398	2.96
504.3433	74.77	PC(18:1/-) –H2O	C26H51O6NP	504.3449	3.07
522.3538	58.46	PC(18:1/-)	C26H53O7NP	522.3554	3.10
577.5174	30.31	PL(16:0/18:1)-Hg	C37H69O4	577.519	2.84
701.3765	13.7	PC(16:0/18:1) -TMA	C39H74O8P	701.5116	192.56
**760.5826**	**68.52**	**PC(16:0/18:1)**	**C42H83O8NP**	**760.5851**	**3.26**

PC(16:0/16:0)* [M+K]+ m/z 772.5253

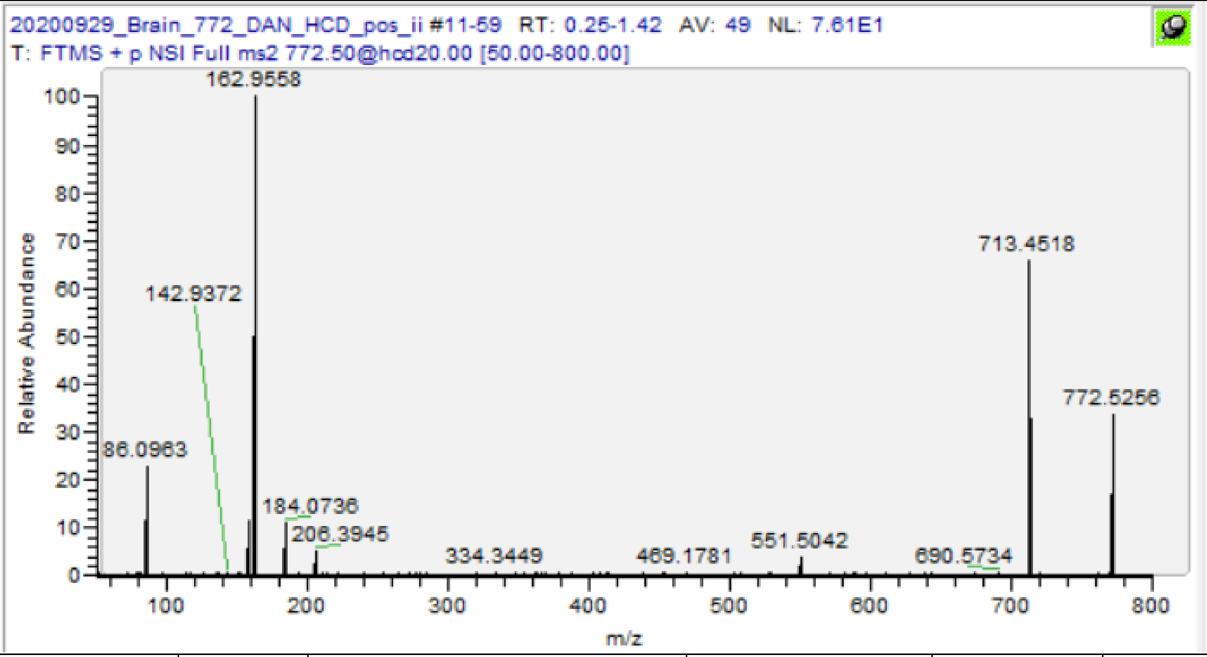

m/z	[I]	Species	Formula	Da	ppm
86.0963	24.16	PC-Frag	C5H12N	86.09643	1.51
162.9558	100	PC-Frag +K	C2H5O4PK	162.9557	-0.61
184.0735	11.33	PC-Hg	C5H15O4NP	184.0733	-0.98
551.5043	2.74	PL(16:0/16:0) -Hg	C35H67O4	551.5034	-1.65
589.4594	0.71	PL(16:0/16:0) -Hg +K	C35H66O4K	589.4593	-0.22
713.4518	76.03	PC(16:0/16:0)-TMA +K	C37H71O8PNa	713.4518	0.01
**772.5254**	**41.02**	**PC(16:0/16:0)+K**	**C40H80O8NPK**	**772.5253**	**-0.12**

PC(16:0/18:1)* [M+Na]+ m/z 782.5670

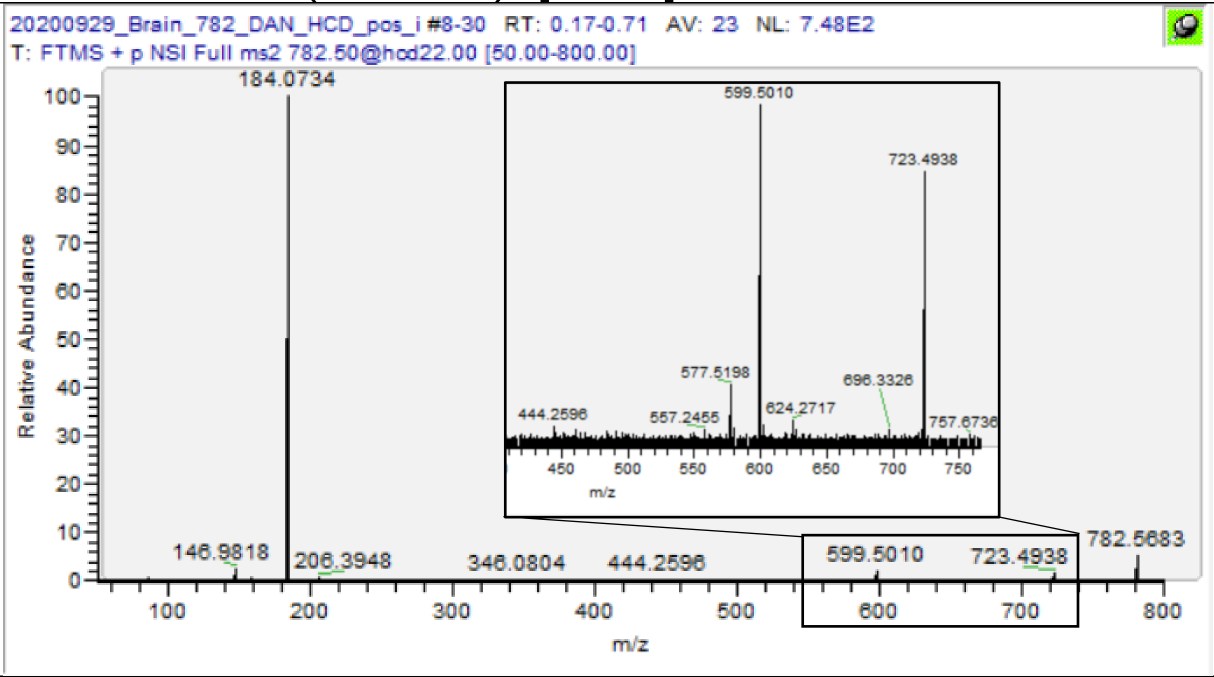

m/z	[I]	Species	Formula	Da	ppm
86.0962	0.5	PC-Frag	C5H12N	86.09643	2.67
184.0734	100	PC-Hg	C5H15O4NP	184.0733	-0.43
577.5200	0.36	PL(16:0/18:1)-Hg	C37H69O4	577.519	-1.66
599.5006	1.42	PL(16:0/18:1)-Hg +Na	C37H69O4Na	599.501	0.63
723.4939	1.93	PC(16:0/18:1)-TMA +Na	C39H73O8PNa	723.4935	-0.51
**782.5685**	**8.33**	**PC(16:0/18:1) +Na**	**C42H82O8NP Na**	**782.567**	**-1.88**

HexCer(d18:1/22:1) [M+H]+ m/z 782.6505

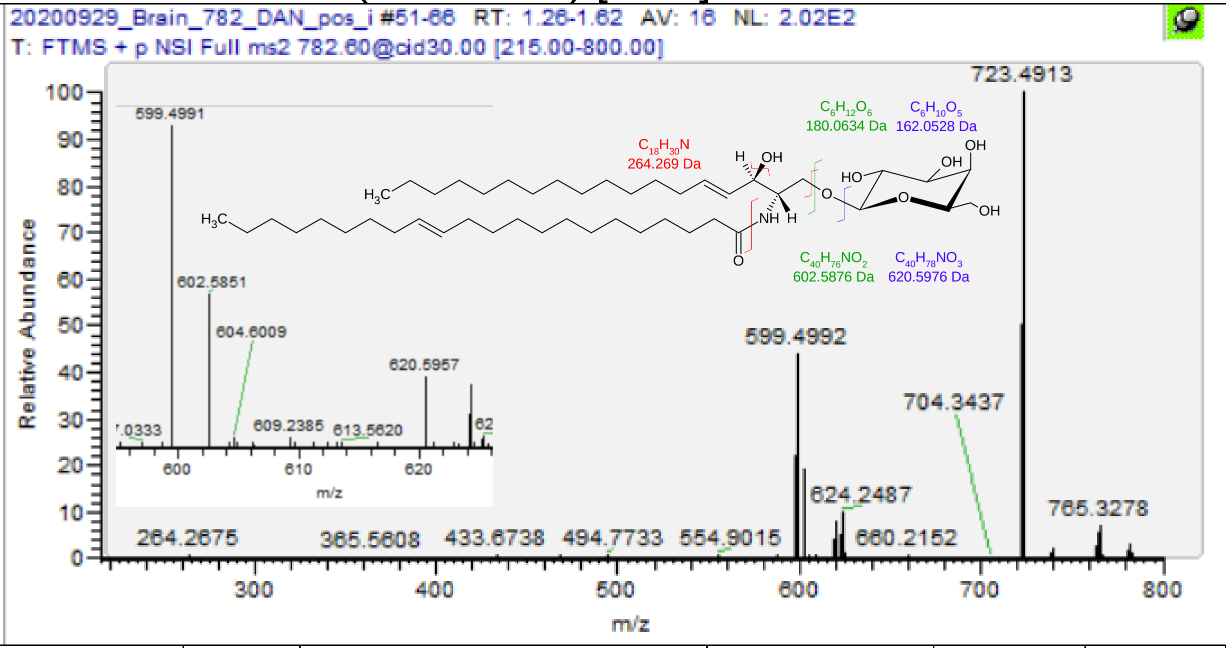

m/z	[I]	Species	Formula	Da	ppm
264.2678	0.59	Cer d18:1 Frag.	C18H34N	264.2686	2.95
602.5853	16.38	HexCer(d18:1/22:1) -Glc -H2O	C40H76O2N	602.5871	2.92
620.5959	7.77	HexCer(d18:1/22:1) -Glc	C40H78O3N	620.5976	2.77
**782.6489**	**3.04**	**HexCer(d18:1/22:1)**	**C46H88O8N**	**782.6505**	**1.98**

SM(d40:2) [M+H]+ m/z 785.6531

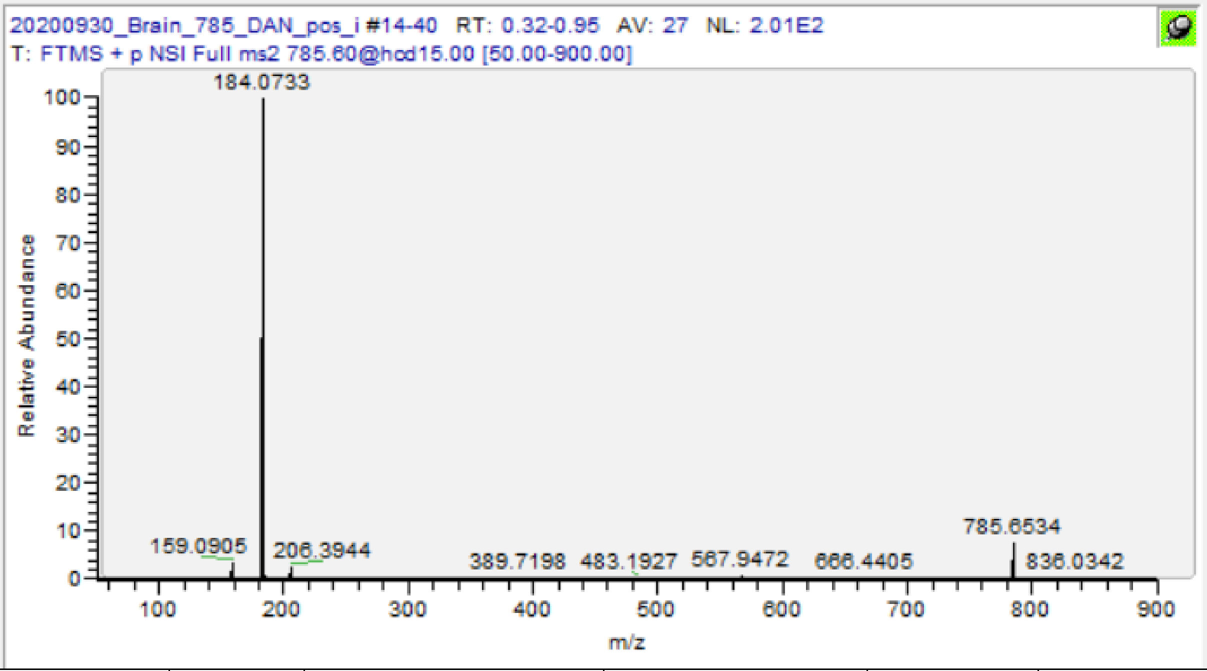

m/z	[I]	Species	Formula	Da	ppm
184.0733	100	PC-Hg	C5H15O4NP	184.0733	0.11
767.6404	9.88	SM(d40:2) -H2O	C45H88O5N2P	767.6425	2.79
**785.6534**	**7.56**	**SM(d40:2)**	**C45H90O6N2P**	**785.6531**	**-0.38**

PC(18:1/18:1) [M+H]+ m/z 786.6007

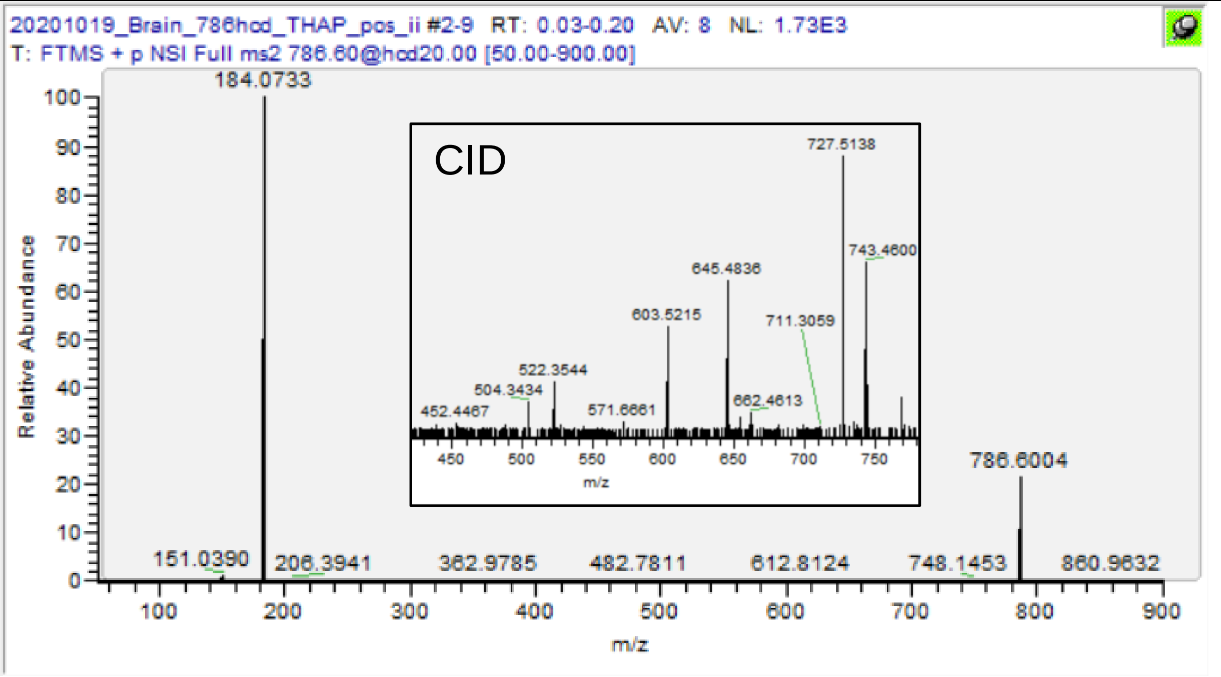

m/z	[I]	Species	Formula	Da	ppm
184.0733	100	PC-Hg	C5H15O4NP	184.0733	0.11
504.3435	9.53	PC(18:1/-) –H2O	C26H51O6NP	504.3449	2.68
522.3542	17.81	PC(18:1/-)	C26H53O7NP	522.3554	2.34
603.5216	29.3	PL(18:1/18:1) -Hg	C39H71O4	603.5347	21.69
726.5106	4.42	PC(18:1/18:1) -TMA	C41H75O8P	726.5194	12.13
**786.6004**	**21.45**	**PC(18:1/18:1)**	**C44H85O8NP**	**786.6007**	**0.42**

PC(18:1/18:0) [M+H]+ m/z 788.6164

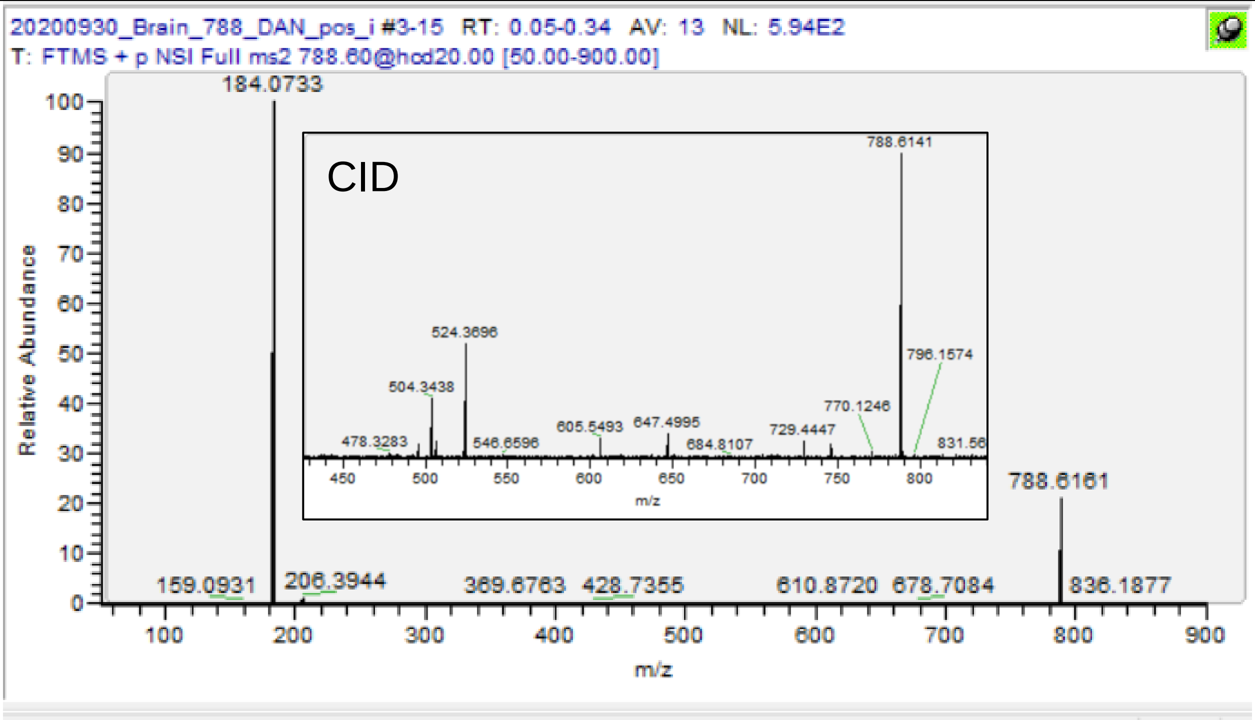

m/z	[I]	Species	Formula	Da	ppm
184.0734	100	PC-Hg	C5H15O4NP	184.0733	-0.43
504.3436	18.46	PC(18:1/-) –H2O	C26H51O6NP	504.3449	2.48
506.3595	5.21	PC(18:0/-) –H2O	C26H53O6NP	506.3605	1.97
522.3544	2	PC(18:1/-)	C26H53O7NP	522.3554	1.95
524.3694	28.73	PC(18:0/-)	C26H55O7NP	524.3711	3.18
605.5483	1.49	PL(18:1/18:0) -Hg	C39H73O4	605.5503	3.37
**788.6161**	**21.36**	**PC(18:1/18:0)**	**C44H87O8NP**	**788.6164**	**0.36**

PE(40:7)* [M+H]+ m/z 790.5381

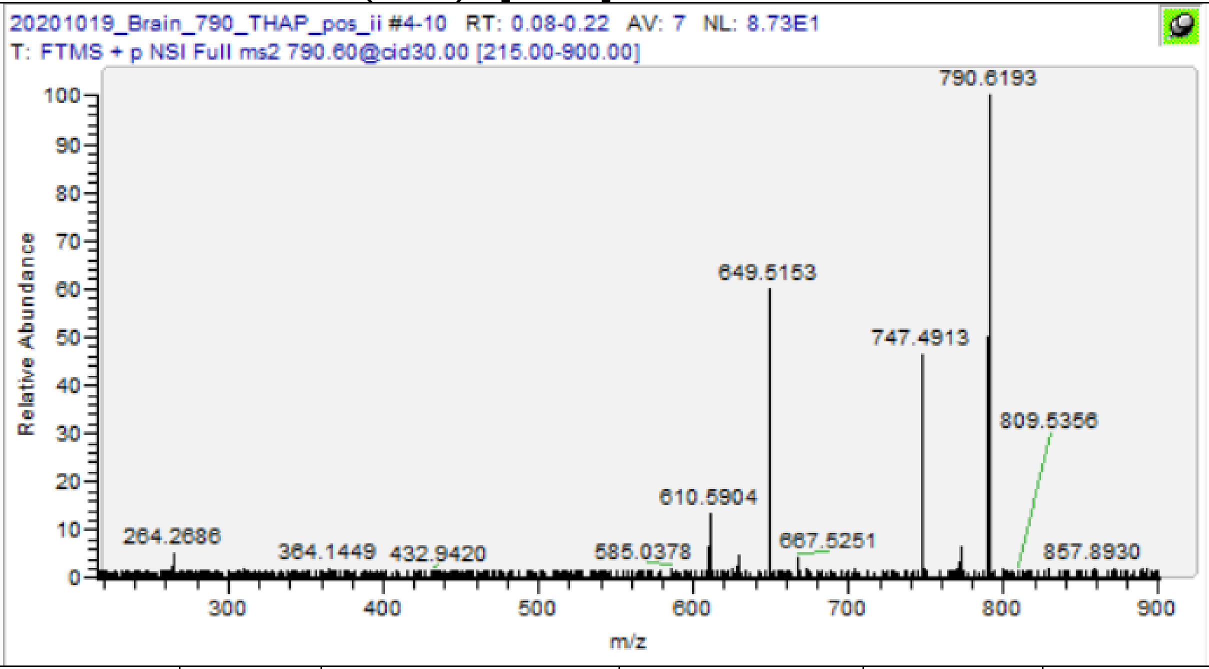

m/z	[I]	Species	Formula	Da	ppm
649.5186	0.36	PL(40:7) -Hg	C43H69O4	649.519	0.68
747.4914	59.81	PA(40:7)	C43H72O8P	747.4959	6.06
**790.5357**	**1.1**	**PE(40:7)**	**C45H77O8NP**	**790.5381**	**3.07**

PE(18:0/22:6) [M+H]+ m/z 792.5538

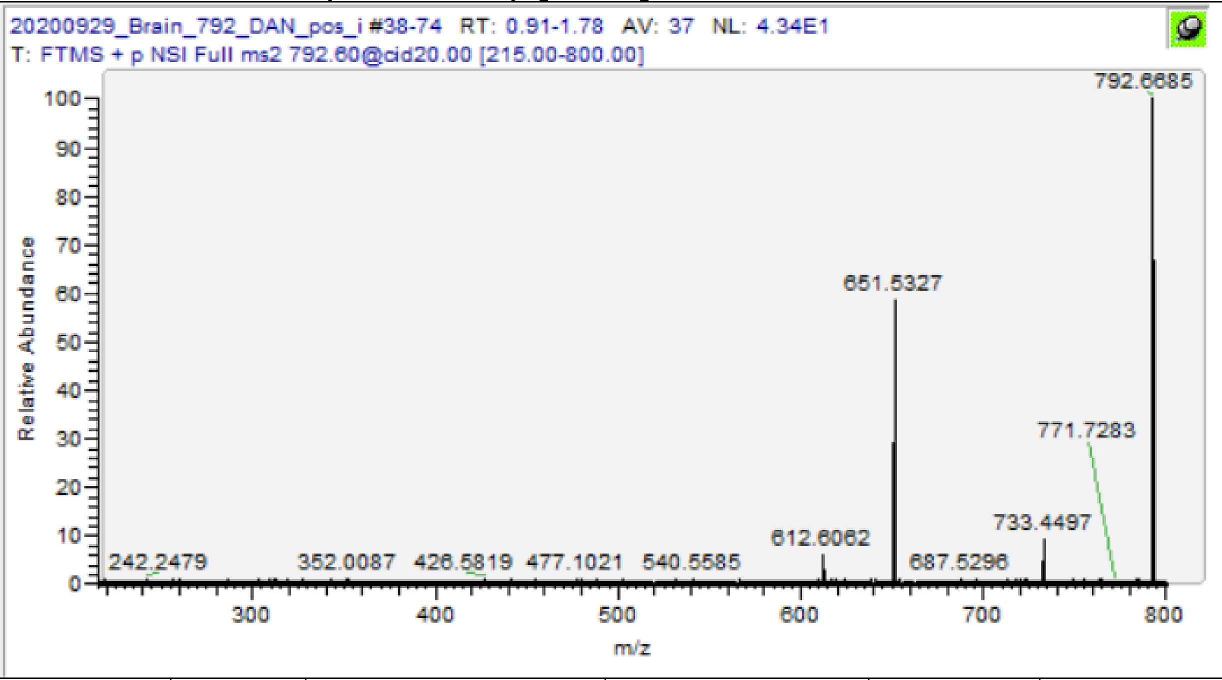

m/z	[I]	Species	Formula	Da	ppm
341.3045	1.29	PL(18:0)-frag	C21H41O3	341.305	1.52
651.5344	59.55	PL(18:0/22:6) -Hg	C43H71O4	651.5347	0.45
**792.5555**	**16.86**	**PE(18:0/22:6)**	**C45H79O8NP**	**792.55378**	**-2.17**

PC(16:0/18:1)* [M+K]+ m/z 798.541

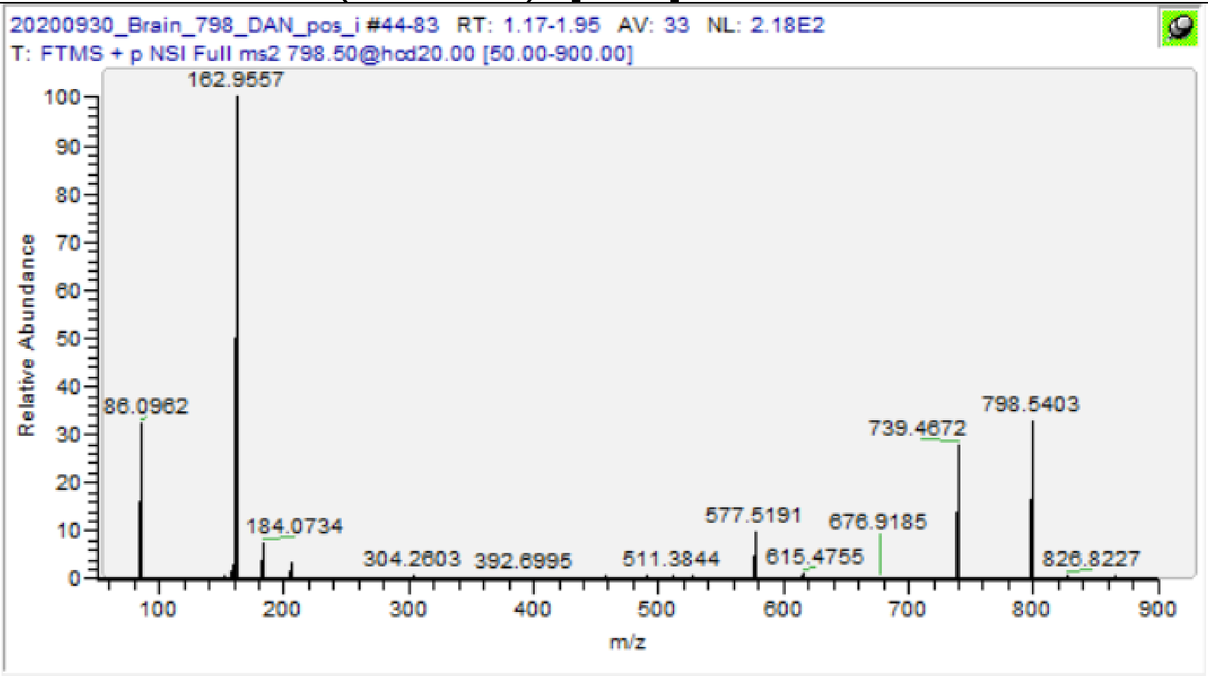

m/z	[I]	Species	Formula	Da	ppm
86.0962	31.94	PC-Frag	C5H12N	86.09643	2.67
162.9557	100	PC-Frag +K	C2H5O4PK	162.9557	0.00
184.0734	7.49	PC-Hg	C5H15O4NP	184.0733	-0.43
577.5191	9.62	PL(16:0/18:1) -Hg	C37H69O4	577.519	-0.10
615.4755	1.25	PL(16:0/18:1) -Hg +K	C37H68O4K	615.4749	-0.94
739.4672	29.96	PC(16:0/18:1) -TMA +K	C39H73O8PK	739.4675	0.35
**798.5403**	**36.79**	**PC(16:0/18:1) +K**	**C42H82O8NPK**	**798.541**	**0.83**

PC(16:0/22:6) [M+H]+ m/z 806.5694

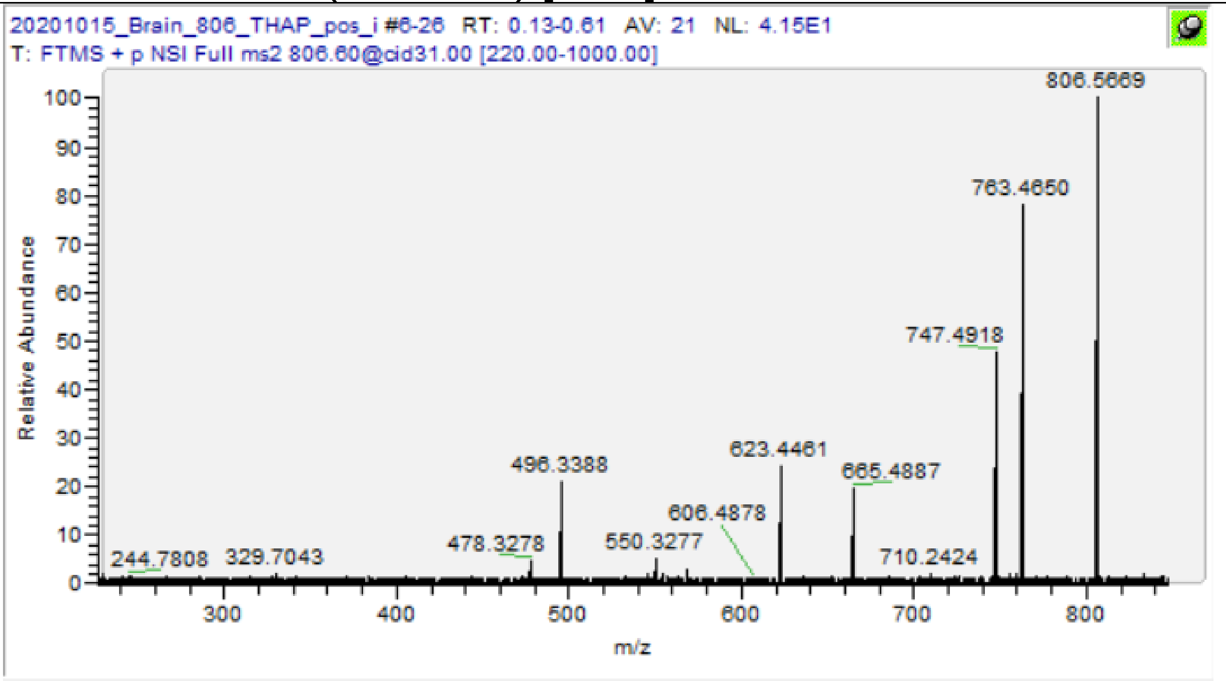

m/z	[I]	Species	Formula	Da	ppm
86.0962	2.72	PC-Frag	C5H12N	86.09643	2.67
124.9997	1.95	PC-Frag	C2H6O4P	124.9998	0.96
184.0733	100	PC-Hg	C5H15O4NP	184.0733	0.11
478.3278	2.96	PC(16:0/-) -H2O	C24H49O6NP	478.3292	2.93
496.3388	19	PC(16:0/-)	C24H51O7NP	496.3398	1.95
550.3277	5.52	PC(22:6/-) -H2O	C30H49O6NP	550.3292	2.73
568.3387	3.85	PC(22:6/-)	C30H51O7NP	568.3398	1.88
623.5001	19.24	PL(16:0/22:6) -Hg	C41H67O4	623.5034	5.28
747.4920	45.28	PC(16:0/22:6) -TMA	C43H72O8P	747.4959	5.26
**806.5691**	**5.47**	**PC(16:0/22:6)**	**C46H81O8NP**	**806.5694**	**0.41**

PE(38:4) [M+K]+ m/z 806.5097

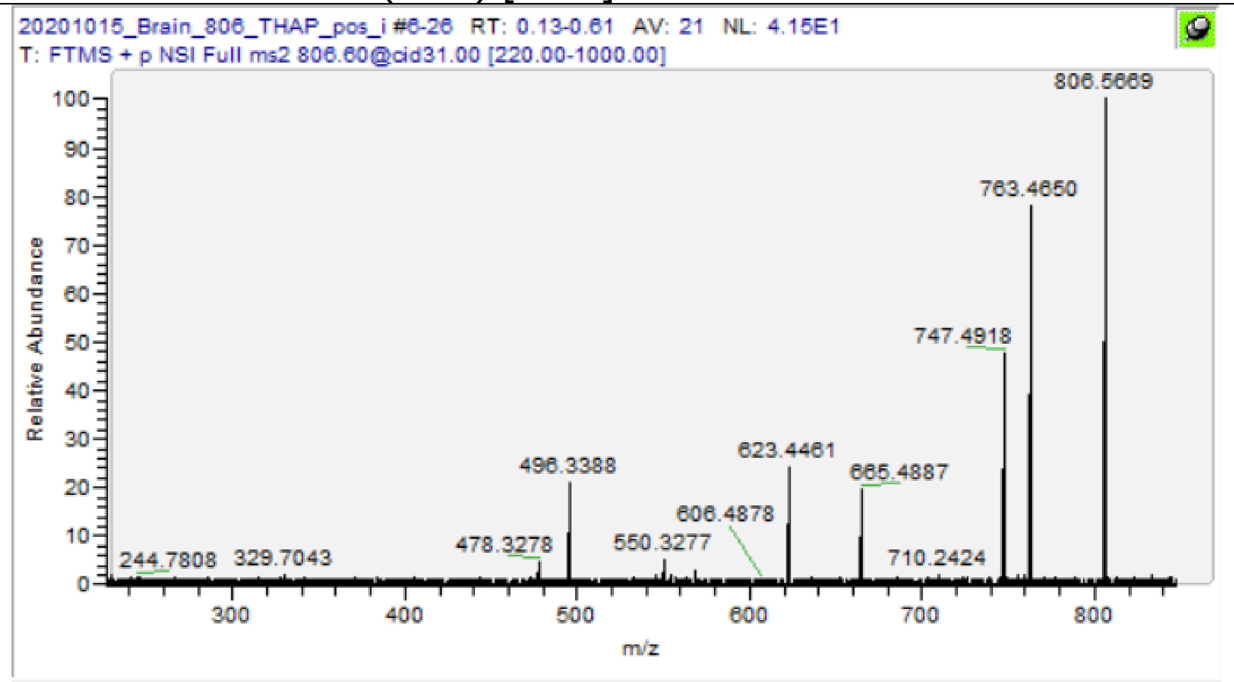

m/z	[I]	Species	Formula	Da	ppm
665.4884	21.08	PL(38:4) -Hg +K	C41H70O4K	665.4906	3.26
763.4652	72.64	PA(38:4) +K	C41H73O8PK	763.4675	2.96
**806.5067**	**21.8**	**PE(38:4) +K**	**C43H78NO8PK**	**806.5097**	**3.67**

PC(18:1/20:4) [M+H]+ m/z 808.5851

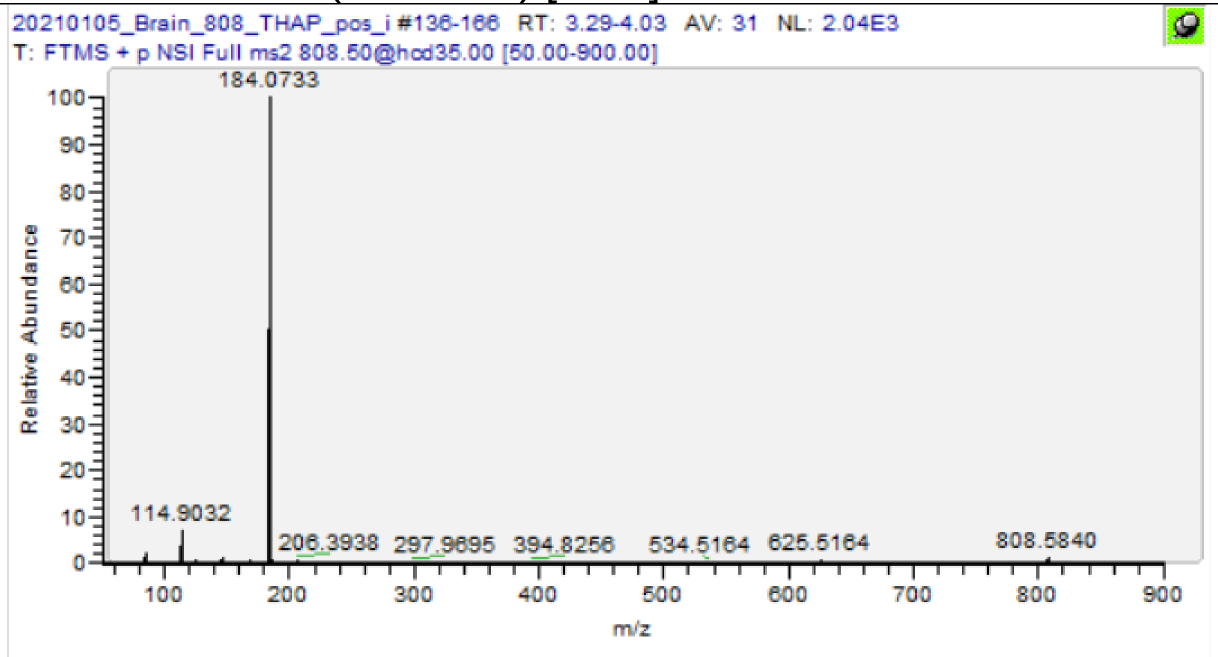

m/z	[I]	Species	Formula	Da	ppm
184.0733	100	PC-Hg	C5H15O4NP	184.0733	0.11
522.3546	0.05	PC(18:1,–)	C26H53O7NP	522.3554	1.56
625.5164	0.55	PC(18:1/20:4) -Hg	C41H69O4	625.519	4.22
749.5094	0.12	PC(18:1/20:4) -TMA	C43H74O8P	749.5116	2.90
**808.5839**	**0.93**	**PC(18:1/20:4)**	**C46H83O8NP**	**808.5851**	**1.45**

HexCer(d18:1/24:2) [M+H]+ m/z 808.6661

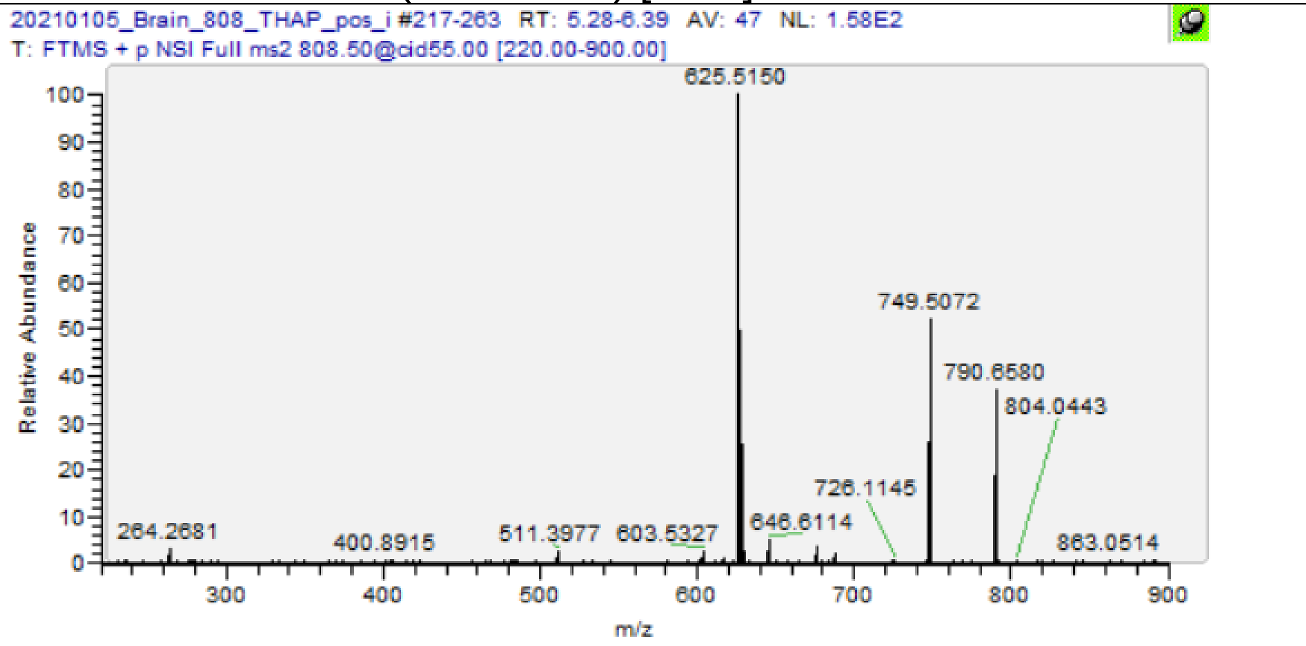

m/z	[I]	Species	Formula	Da	ppm
264.2685	0.7	Cer d18:1 Frag.	C18H34N	264.2686	0.30
628.601	6.07	HexCer(d18:1/24:2) -Glc -H2O	C42H78O2N	628.6027	2.72
646.6114	1.59	HexCer(d18:1/24:2) -Glc	C42H80O3N	646.6133	2.89
790.658	21.41	HexCer(d18:1/24:2) -H2O	C48H88O7N	790.6555	-3.12
**808.6642**	**1.22**	**HexCer(d18:1/24:2)**	**C48H90O8N**	**808.6661**	**2.34**

SM(d42:2) [M+H]+ m/z 813.6844

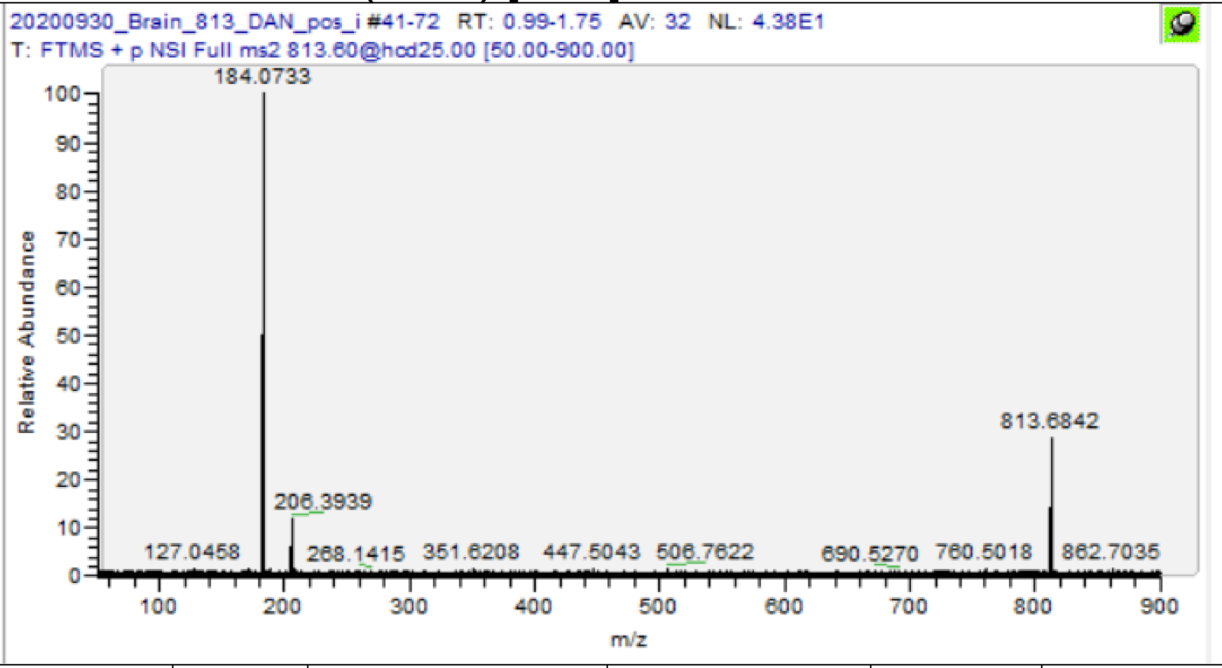

m/z	[I]	Species	Formula	Da	ppm
184.0733	100	PC-Hg	C5H15O4NP	184.0733	0.11
**813.6846**	**27.74**	**SM (d42:2)**	**C47H94O6N2P**	**813.6844**	**-0.25**

PC(18:1/18:0)* [M+K]+ m/z 826.5723

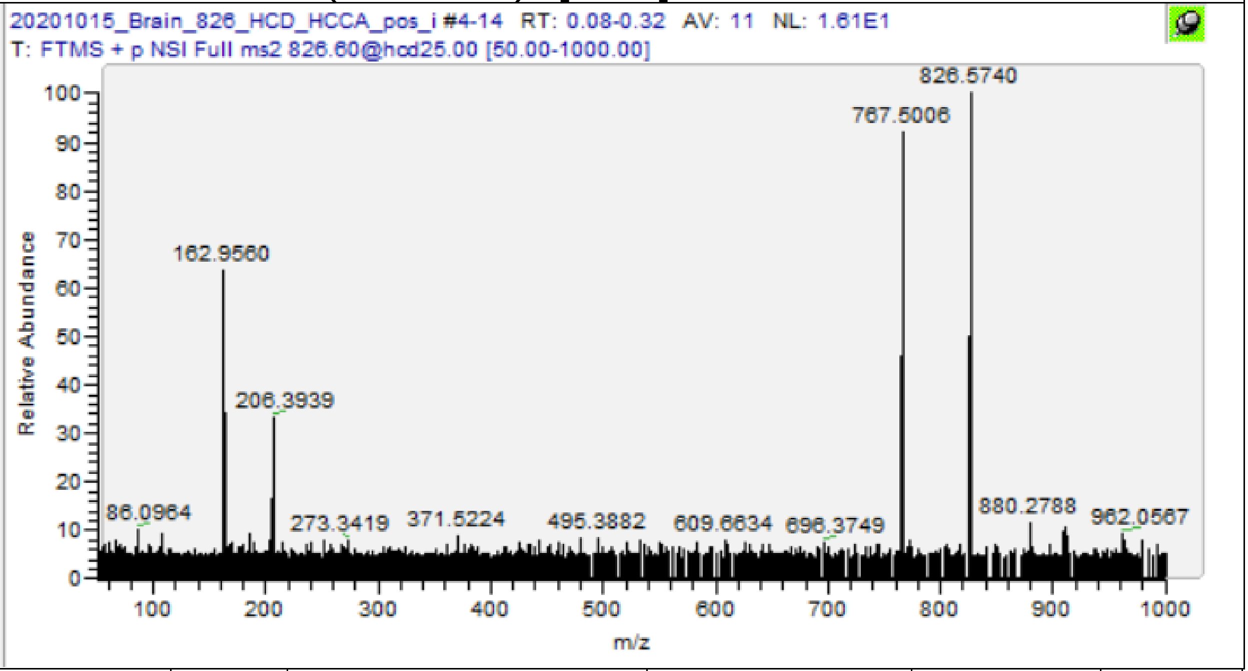

m/z	[I]	Species	Formula	Da	ppm
86.0964	8.75	PC-Frag	C5H12N	86.09643	0.35
162.9560	55.72	PC-Frag +K	C2H5O4PK	162.9557	-1.84
643.5067	4.04	PL(18:1/18:0) -Hg +K	C39H72O4K	643.5062	-0.75
767.4998	85.7	PC(18:1/18:0) -TMA +K	C41H77O8PK	767.4988	-1.36
**826.5739**	**100**	**PC(18:1/18:0) +K**	**C44H86O8NPK**	**826.5723**	**-1.98**

PC(18:0/22:6) [M+H]+ m/z 834.6007

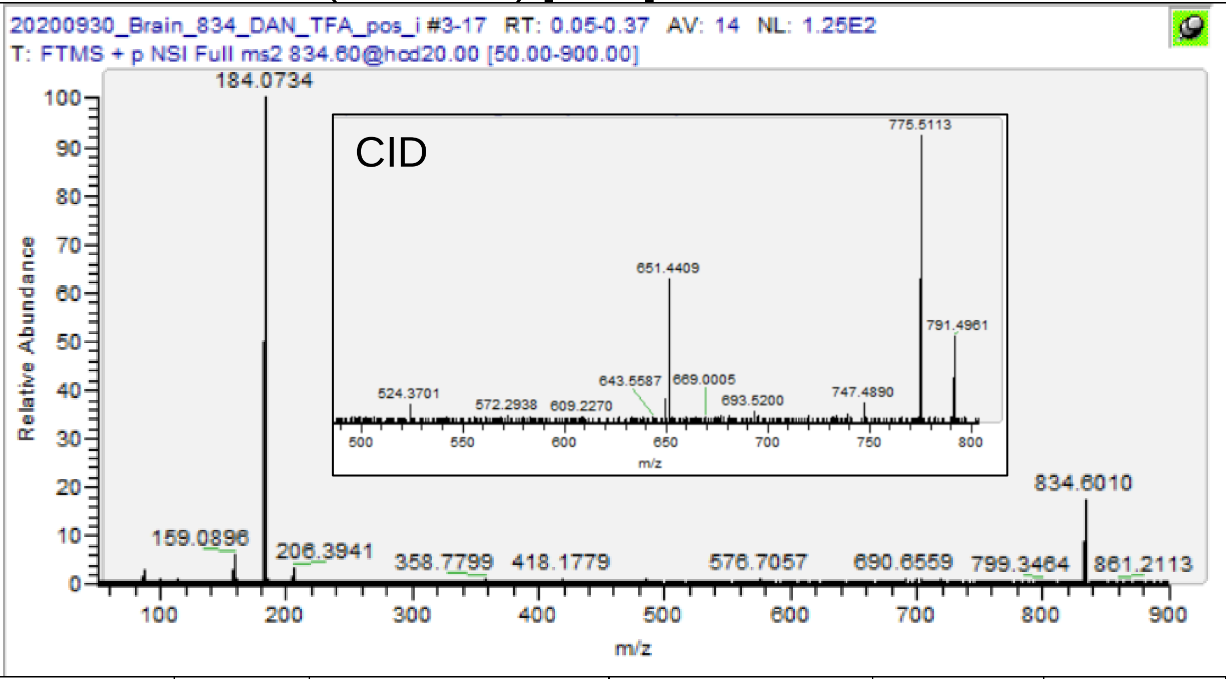

m/z	[I]	Species	Formula	Da	ppm
184.0734	100	PC-Hg	C5H15O4NP	184.0733	-0.43
524.3701	1.25	PC(18:0,–)	C26H55O7NP	524.3711	1.84
651.5218	6.72	PC(18:0/22:6) -Hg	C43H71O4	651.5347	19.78
775.5115	23.48	PC(18:0/22:6) -TMA	C45H76O8P	775.5272	20.282
**834.6012**	**16.55**	**PC(18:0/22:6)**	**C48H85O8NP**	**834.6007**	**-0.56**

PC(38:6) [M+K]+ m/z 844.5253

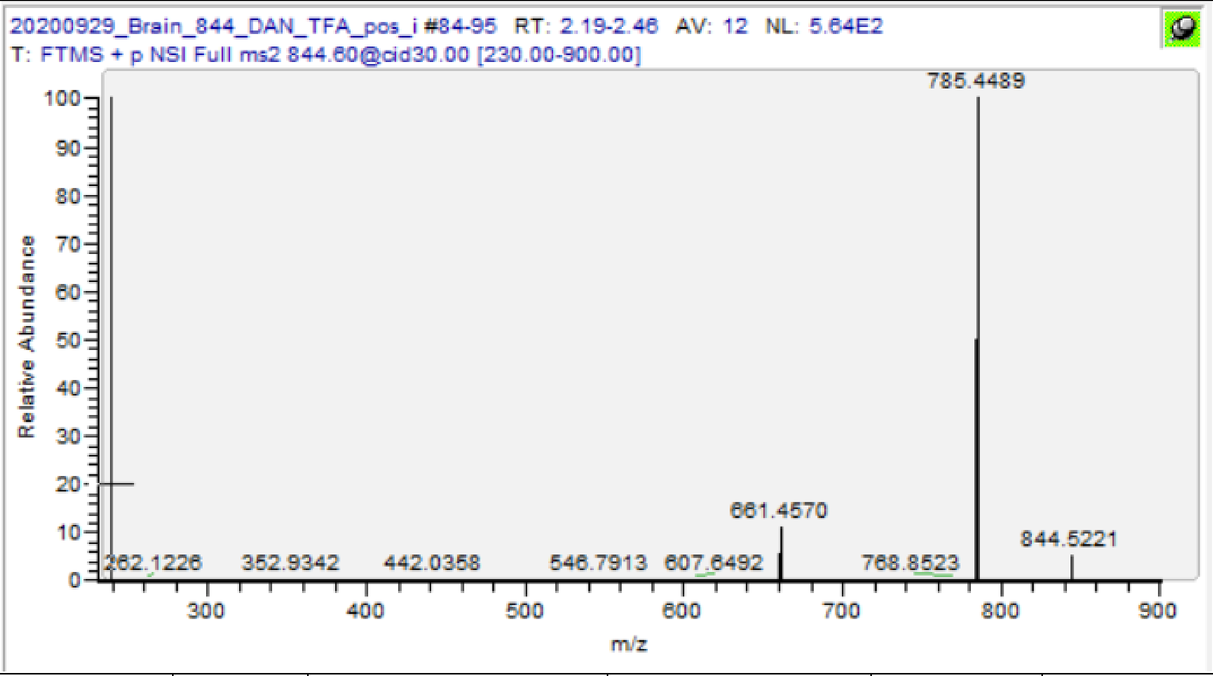

m/z	[I]	Species	Formula	Da	ppm
661.4575	11.35	PL(38:6) -Hg +K	C41H66O4K	661.4593	2.68
785.4495	100	PC(38:6) -TMA +K	C43H71O8PK	785.4518	2.94
**844.5228**	**21.36**	**PC(38:6) +K**	**C46H80NO8PK**	**844.5253**	**2.97**

PC(38:4) [M+K]+ m/z 848.5566

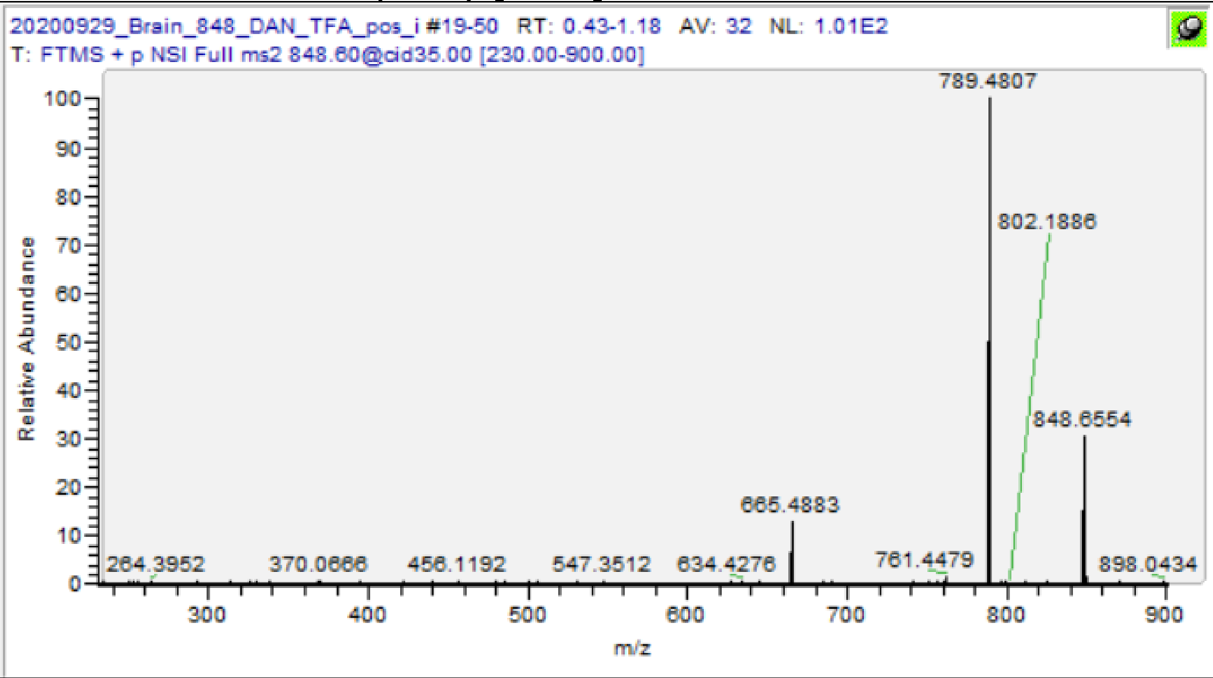

m/z	[I]	Species	Formula	Da	ppm
665.4885	12.82	PL(38:4) -Hg +K	C41H70O4K	665.4906	3.11
789.4807	100	PC(38:4) -TMA +K	C43H75O8PK	789.4831	3.05
**848.5556ᵞ**	**15.12**	**PC(38:4) +K**	**C46H84O8NPK**	**848.5566**	**1.19**

ᵞ Value from MS1 scan

HexCer(d18:1/24:1(2OH)) [M+Na]+ m/z 848.6586

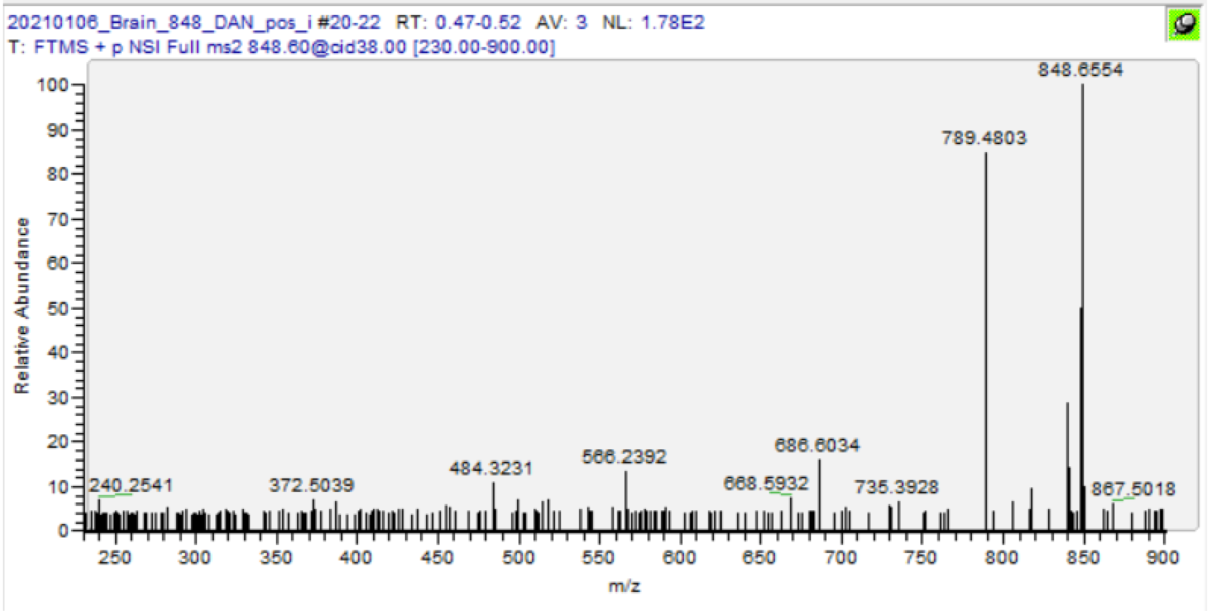

m/z	[I]	Species	Formula	Da	ppm
484.3231	3.82	HexCer(d18:1/24:1(2OH)) -C24 + Na	C24H47O7NNa	484.3245	2.83
668.5932	1.79	HexCer(d18:1/24:1(2OH)) -Glc -H2O + Na	C42H79O3NNa	668.5952	3.02
686.6034	4.79	HexCer(d18:1/24:1(2OH)) -Glc + Na	C42H81O4NNa	686.6058	3.47
**848.6564**	**100**	**HexCer(d18:1/24:1(2OH)) + Na**	**C48H91O9NNa**	**848.6586**	**2.59**

PC(40:6) [M+K]+ m/z 872.5566

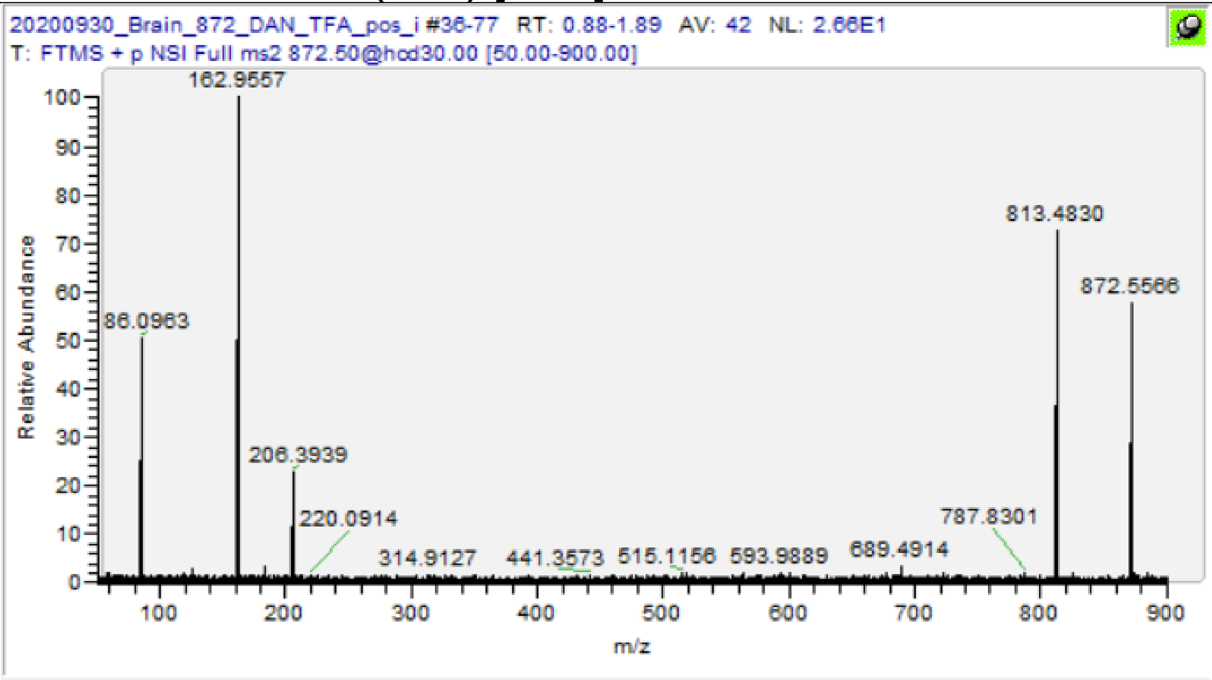

m/z	[I]	Species	Formula	Da	ppm
86.0963	49.49	PC-Frag	C5H12N	86.09643	1.51
162.9557	100	PC-Frag +K	C2H5O4PK	162.9557	0.00
184.0735	4	PC-Hg	C5H15O4NP	184.0733	-0.98
689.4914	4.1	PL(40:6) -Hg +K	C43H70O4K	689.4906	-1.20
813.4829	97.88	PC(40:6) -TMA +K	C45H75O8PK	813.4831	0.26
**872.5565**	**77.68**	**PC(40:6) +K**	**C48H85O8NPK**	**872.5566**	**0.13**

PC(16:0/18:1)+PC(16:0/16:0) [M+M+H]+ m/z 1494.147

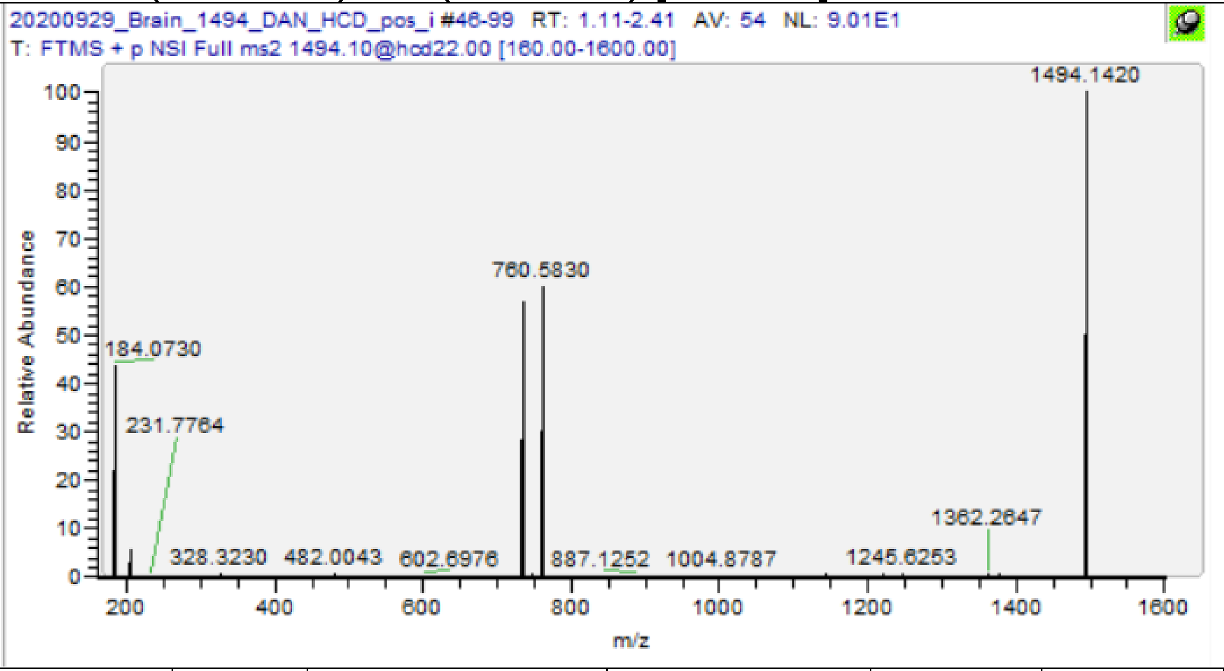

m/z	[I]	Species	Formula	Da	ppm
184.0736	27.75	PC-Hg	C5H15O4NP	184.0733	-1.52
734.5698	96.83	PC(16:0/16:0)	C40H81O8NP	734.5694	-0.50
760.5859	100	PC(16:0/18:1)	C42H83O8NP	760.5851	-1.08
**1494.1488**	**27.75**	**PC(16:0/18:1) +PC(16:0/16:0)**	**C82H163O16N2P2**	**1494.147**	**-1.04**

PC(16:0/18:1) [2M+H]+ m/z 1520.163

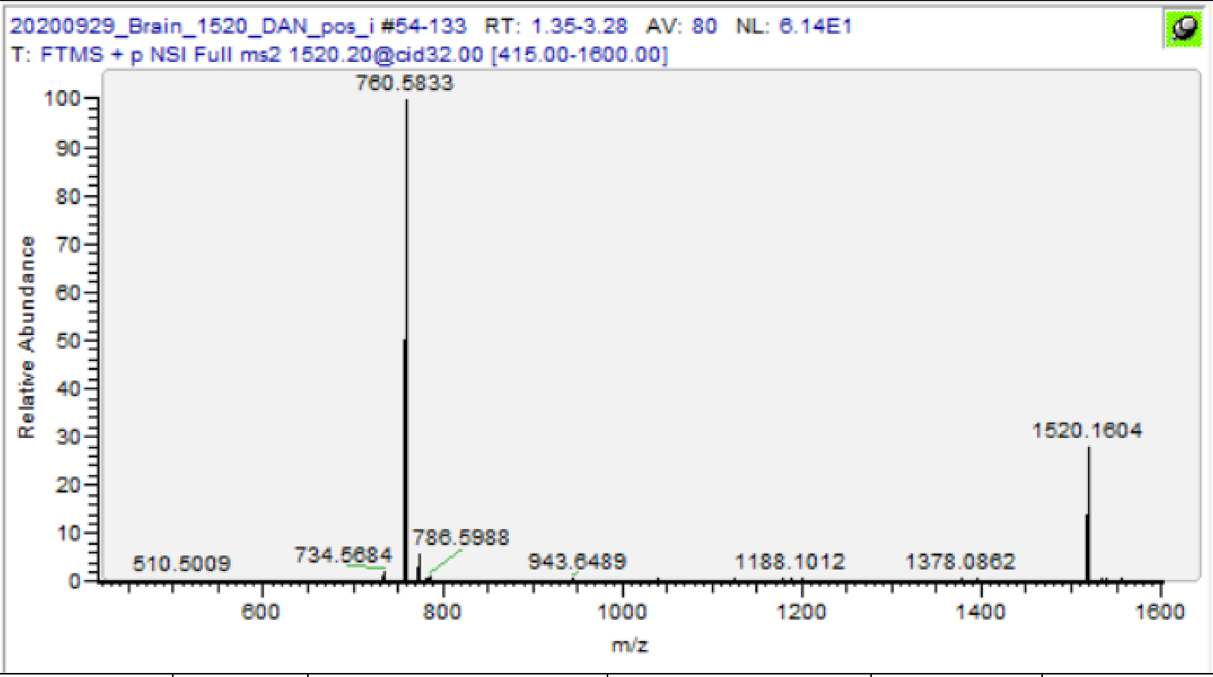

m/z	[I]	Species	Formula	Da	ppm
86.0964	1.86	PC-Frag	C5H12N	86.09643	0.35
124.9999	4.2	PC-Frag	C2H6O4P	124.9998	-0.64
184.0735	100	PC-Hg	C5H15O4NP	184.0733	-0.98
760.5833	100	PC(16:0/18:1)	C42H83O8NP	760.5851	2.34
**1520.1604**	**26.74**	**2 xPC(16:0/18:1)**	**C84H165O16N2P2**	**1520.163**	**1.64**
734.5683	2.3	PC(16:0/16:0)	C40H81O8NP	734.5694	1.54
786.5991	1.14	PC(18:1/18:1)	C44H85O8NP	786.6007	2.07

PC(18:1/18:0)+PC(16:0/18:1) [M+M+H]+ m/z 1548.194

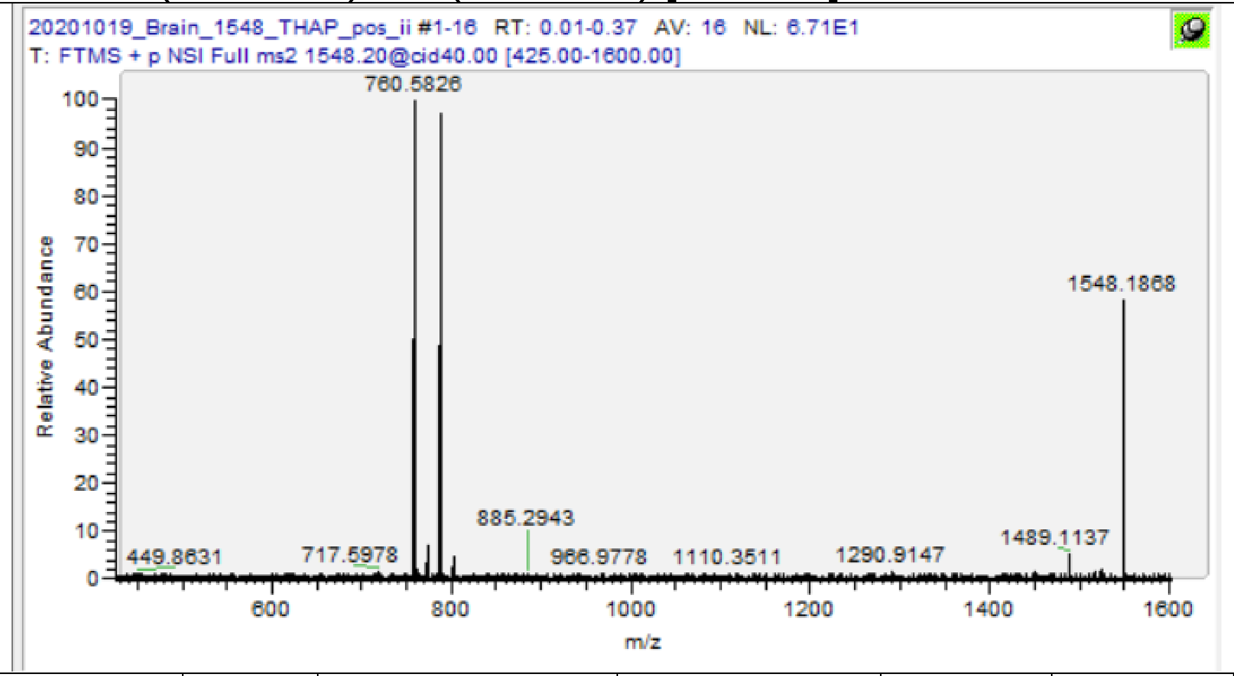

m/z	[I]	Species	Formula	Da	ppm
760.5826	100	PC(16:0/18:1)	C42H83O8NP	760.5851	3.26
788.6141	97.64	PC(18:1/18:0)	C44H87O8NP	788.6164	2.89
**1548.1868**	**54.73**	**PC(18:1/18:0) +PC(16:0/18:1)**	**C86H169O16N2P2**	**1548.194**	**4.64**

Negative ion mode MS/MS, Identified species

Cer(d18:0/18:1) [M-H]- m/z 564.535

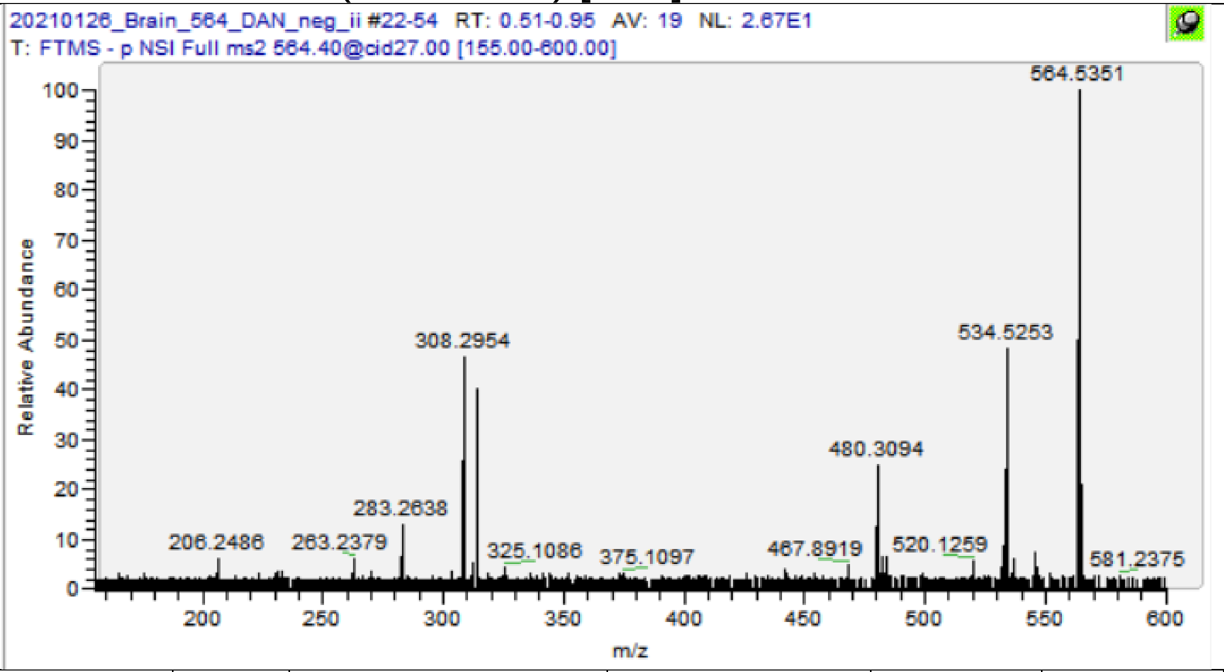

m/z	[I]	Species	Formula	Da	ppm
263.2378	2.17	Cer(18:1)-Frag	C18H31O	263.2369	-3.27
283.2638	3.37	FA 18:0	C18H35O2	283.2643	1.59
308.2954	14.08	Cer(18:1)-Frag	C20H38ON	308.2948	-1.98
534.5253	12.7	Cer(d18:0/18:1) -COH2	C35H68O2N	534.5245	-1.57
**564.535**	**100**	**Cer(d18:0/18:1)**	**C36H70O3N**	**564.535**	**0.04**

CerP(d18:1/18:0) [M-H]- m/z 644.5025

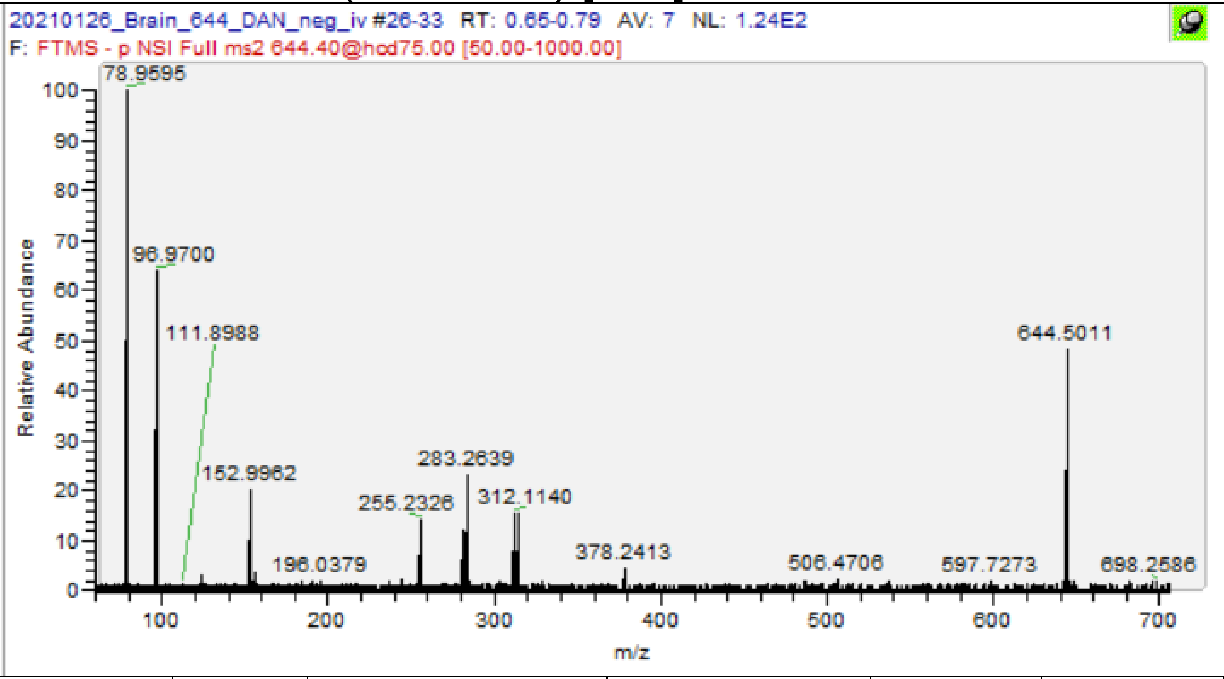

m/z	[I]	Species	Formula	Da	ppm
78.9595	100	PL-Frag	PO3	78.95905	-5.70
96.97	64.59	PL-Frag	H2O4P	96.96962	-3.92
152.9962	20.18	PL-Frag	C3H6O5P	152.9958	-2.42
378.2412	12.94	CerP(d18:1)	C18H37O5NP	378.2415	0.74
**644.5019**	**100**	**CerP(d18:1/18:0)**	**C36H71NO6P**	**644.5025**	**0.85**

PA(16:0/16:1) [M-H]- m/z 645.4501

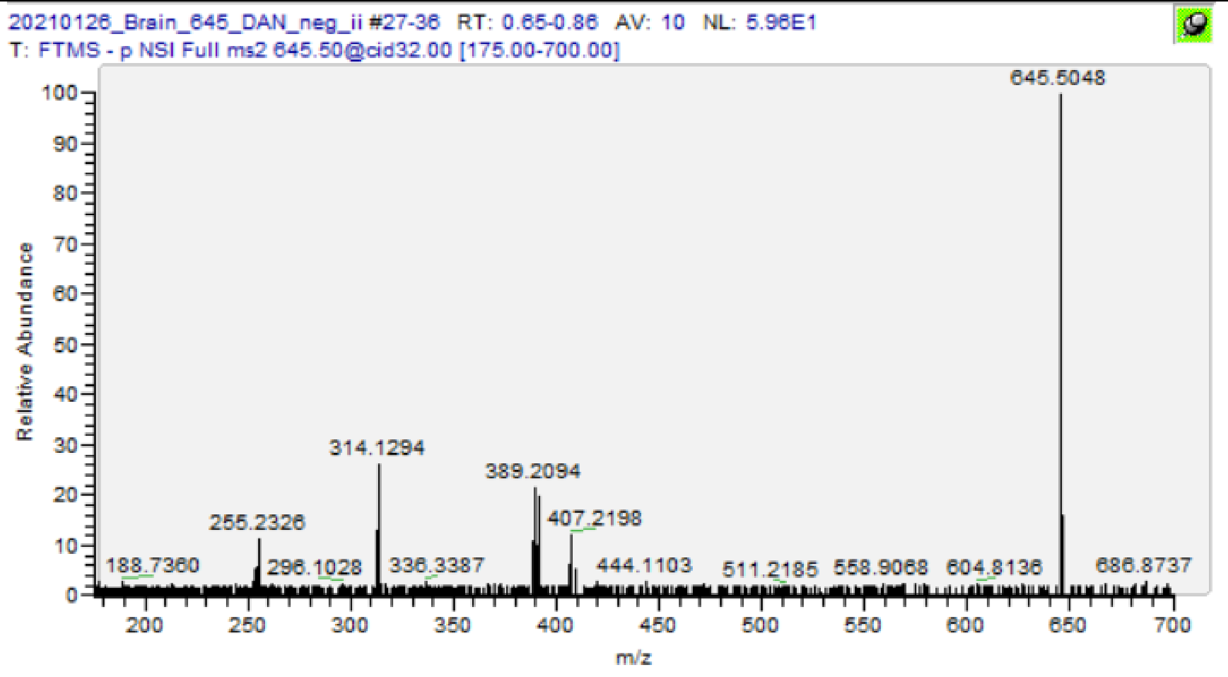

m/z	[I]	Species	Formula	Da	ppm
253.2169	4.2	FA 16:1	C16H29O2	253.2173	1.58
255.2326	13.04	FA 16:0	C16H31O2	255.233	1.37
389.2094	17.37	PA(16:1) -H2O	C19H34O6P	389.2099	1.16
391.225	16	PA(16:0) -H2O	C19H36O6P	391.2255	1.28
407.2198	12.5	PA(16:1)	C19H36O7P	407.2204	1.50
409.2355	7.71	PA(16:0)	C19H38O7P	409.2361	1.37
**645.4503**	**3.83**	**PA(16:0/16:1)**	**C35H66O8P**	**645.4501**	**-0.34**

PA(16:0/16:0) [M-H]- m/z 647.4657

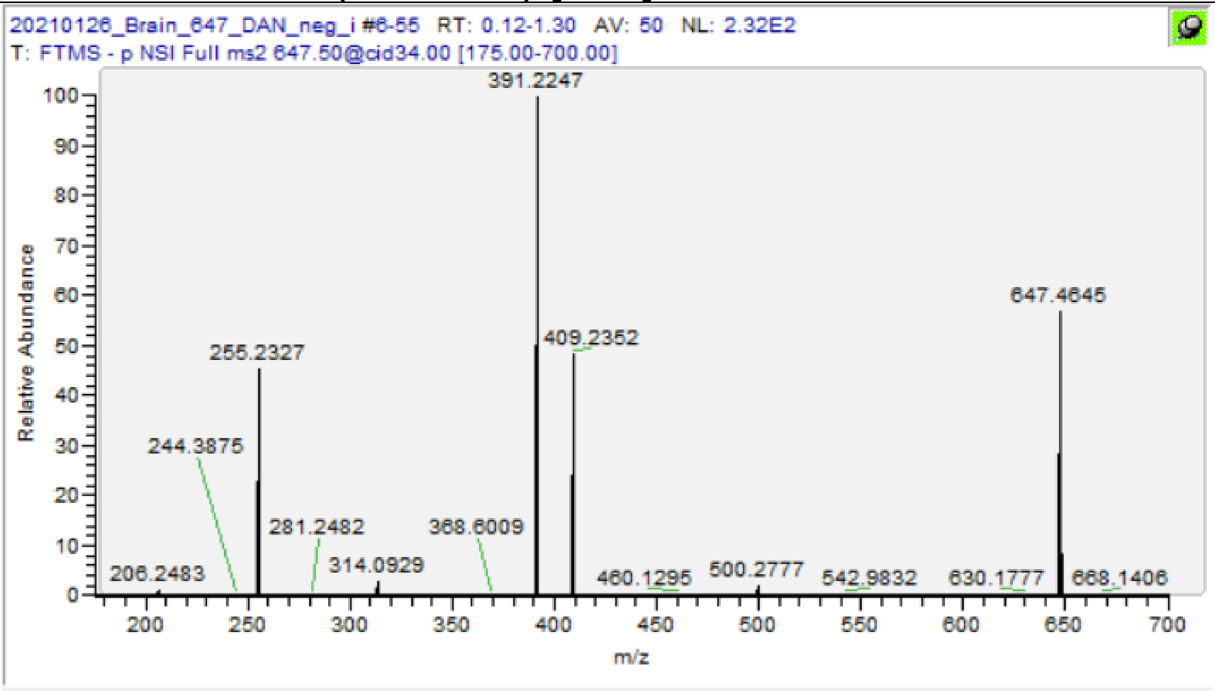

m/z	[I]	Species	Formula	Da	ppm
255.2327	53.81	FA 16:0	C16H31O2	255.233	0.98
391.2247	100	PA(16:0) -H2O	C19H36O6P	391.2255	2.04
409.2352	53.7	PA(16:0)	C19H38O7P	409.2361	2.10
**647.4645**	**55.4**	**PA(16:0/16:0)**	**C35H68O8P**	**647.4657**	**1.90**

CerP(d38:2) [M-H]- m/z 670.5181

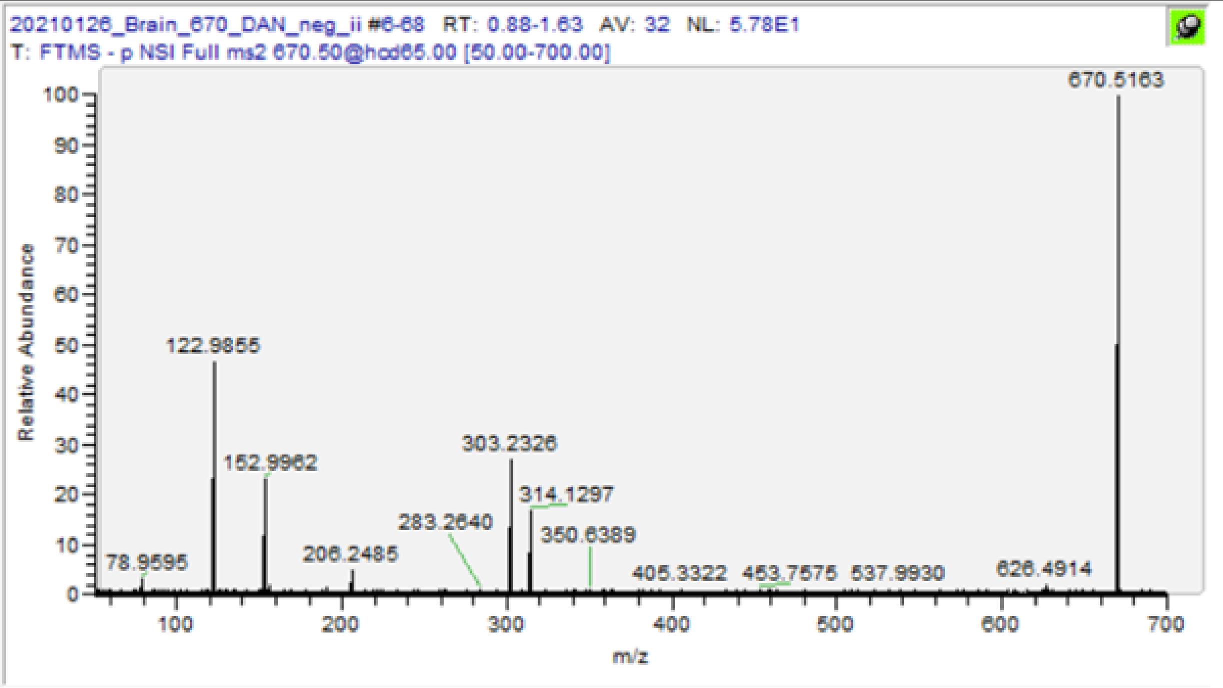

m/z	[I]	Species	Formula	Da	ppm
78.9595	5.49	PL-Frag	PO3	78.95905	-5.70
122.9855	63.42	PL-Frag	C2H4O4P	122.9853	-1.87
152.9962	30.12	PL-Frag	C3H6O5P	152.9958	-2.42
626.4922	2.18	CerP 36:1;O2 -H2O	C36H69O5NP	626.4919	-0.51
**670.5173**	**34.76**	**CerP(d38:2)**	**C38H73O6NP**	**670.5181**	**1.19**

PA(16:0/18:1) [M-H]- m/z 673.4814

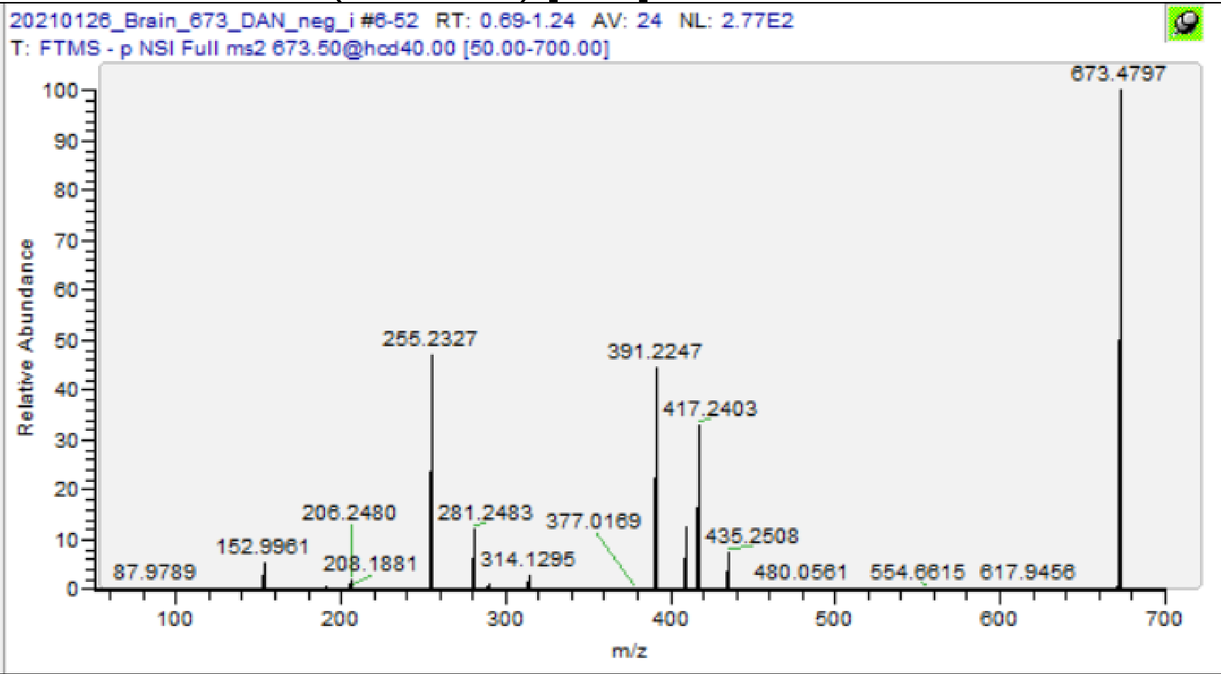

m/z	[I]	Species	Formula	Da	ppm
152.9962	9.48	PL-Frag	C3H6O5P	152.9958	-2.42
255.2327	79.48	FA 16:0	C16H31O2	255.233	0.98
281.2483	23.66	FA 18:1	C18H33O2	281.2486	1.07
391.2246	62.74	PA(16:0) -H2O	C19H36O6P	391.2255	2.30
409.2352	19.64	PA(16:0)	C19H38O7P	409.2361	2.10
417.2402	48.62	PA(18:1) -H2O	C21H38O6P	417.2412	2.28
435.2506	12.22	PA(18:1)	C21H40O7P	435.2517	2.55
**673.4795**	**100**	**PA(16:0/18:1)**	**C37H70O8P**	**673.4814**	**2.79**

SM(d34:1) [M-CH3]- m/z 687.5447

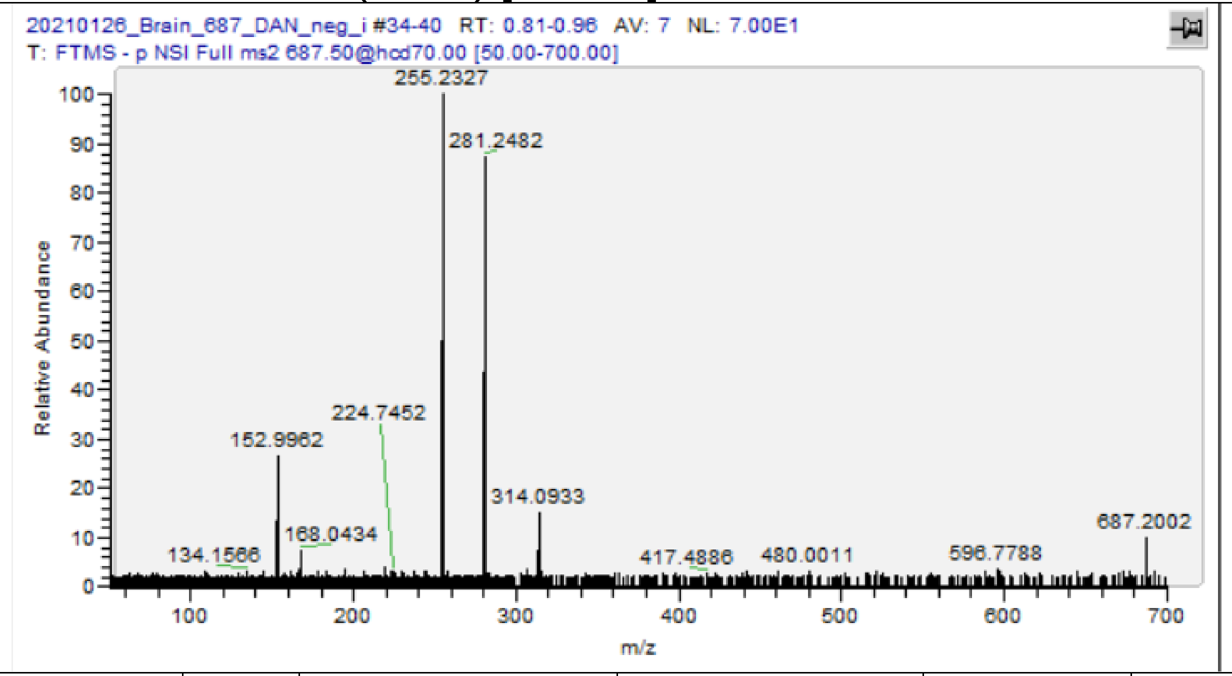

m/z	[I]	Species	Formula	Da	ppm
168.0434	7.87	PC-Hg	C4H11O4NP	168.0431	-1.67
255.2327	86.39	FA 16:0	C16H31O2	255.233	0.98
281.2483	100	FA 18:1	C18H33O2	281.2486	1.07
**687.5426**	**18.33**	**SM(d18:1/16:0) -CH3 / SM(d16:0/18:1) -CH3**	**C38H76O6N2P**	**687.5447**	**2.98**

SM(d18:1/18:0) [M-CH3]- m/z 715.576

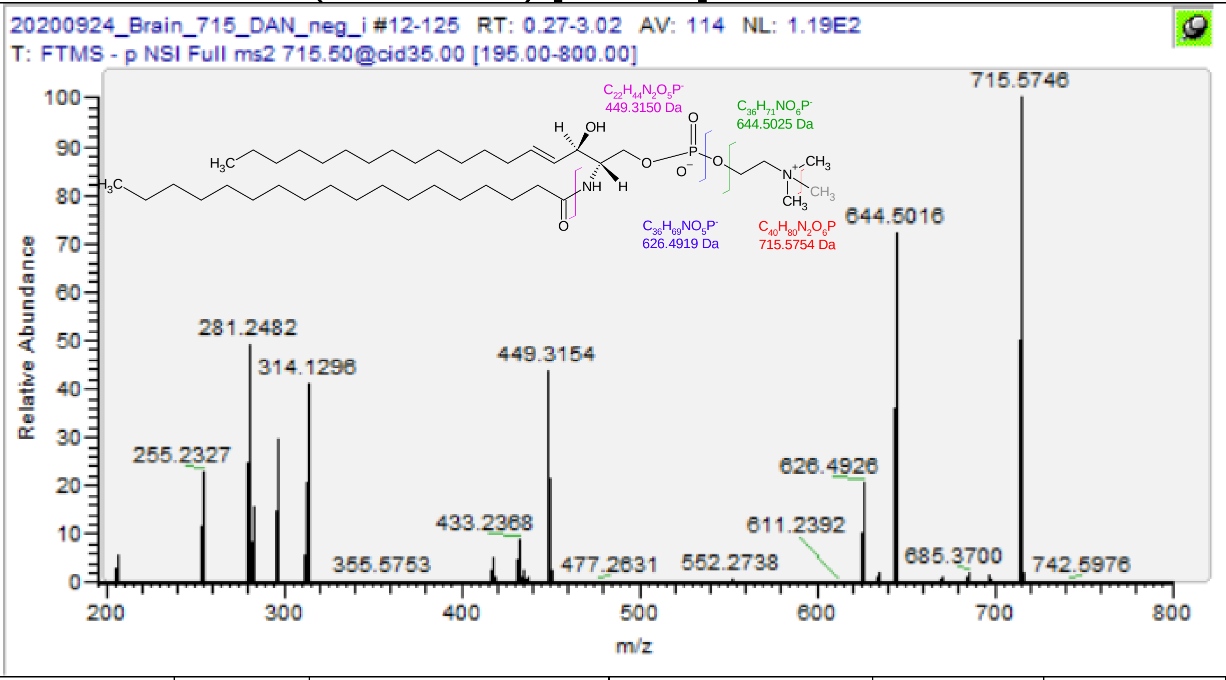

m/z	[I]	Species	Formula	Da	ppm
449.3155	60.64	LSM(18:1);O2 -CH3	C22H46N2O5P	449.315	-1.16
626.4927	28.36	CerP 36:1;O2 -H2O	C36H69O5NP	626.4919	-1.31
644.5017	100	CerP 36:1;O2	C36H71NO6P	644.5025	1.16
**715.5750**	**55.63**	**SM(d18:1/18:0) -CH3**	**C40H80O6N2P**	**715.576**	**1.33**
255.2327	33.11	FA(16:0)	C16H31O2	255.233	0.98
281.2482	68.71	FA(18:1)	C18H33O2	281.2486	1.42
283.2642	21.79	FA(18:0)	C18H35O2	283.2643	0.18
297.2433	40.61	FA(18:1);O	C18H33O3	297.2435	0.74
417.2417	6.56	PA(18:1) -H2O	C21H38O6P	417.2412	-1.32
433.2368	13.01	PA(18:2)	C21H38O7P	433.2361	-1.71
435.2523	3	PA(18:1)	C21H40O7P	435.2517	-1.36
437.2674	1.75	PA(18:0)	C21H42O7P	437.2674	-0.09

PE(16:0/18:0) [M-H]- m/z 718.5392

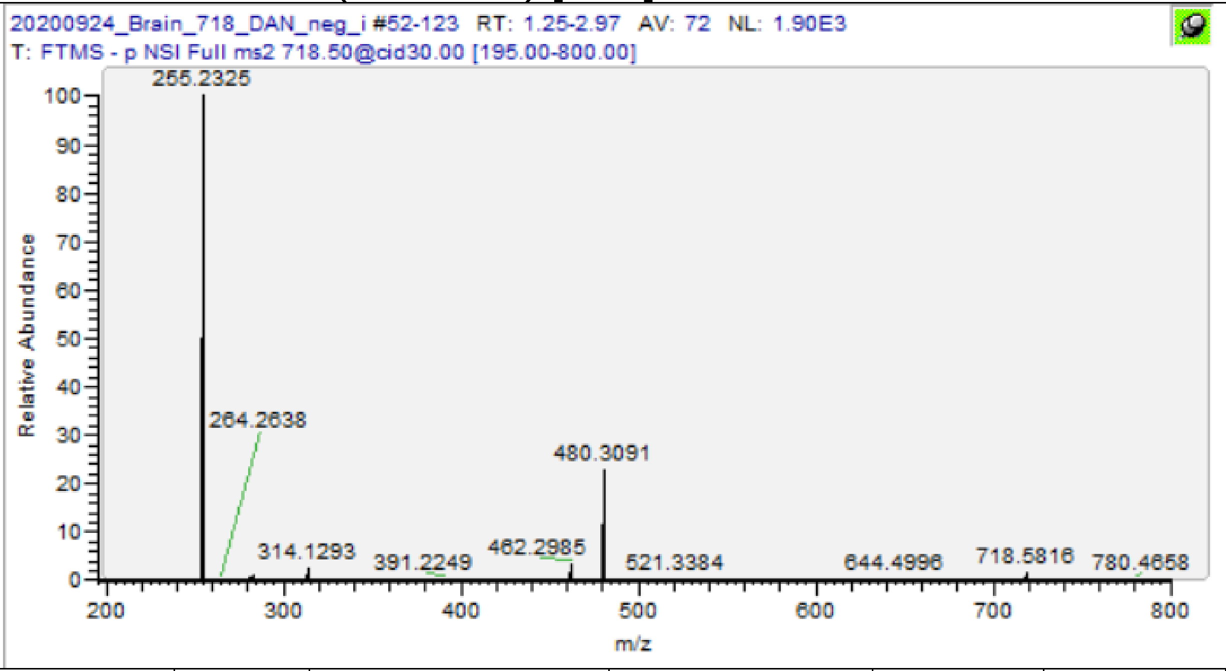

m/z	[I]	Species	Formula	Da	ppm
255.2325	100	FA(16:0)	C16H31O2	255.233	1.76
283.2638	0.96	FA(18:0)	C18H35O2	283.2643	1.59
419.2559	0.06	PA(18:0) -H2O	C21H40O6P	419.2568	2.15
437.2662	0.06	PA(18:0/-)	C21H42O7P	437.2674	2.65
462.2985	3.6	PE(18:0) -H2O	C23H45O6NP	462.299	1.08
480.3091	23.25	PE(18:0)	C23H47O7NP	480.3096	0.96
**718.5377ɣ**	**1.84**	**PE(16:0/18:0)**	**C39H77O8NP**	**718.5392**	**2.13**

ɣ Value from MS1 scan

PE(P-18:1/18:1) [M-H]- m/z 726.5443

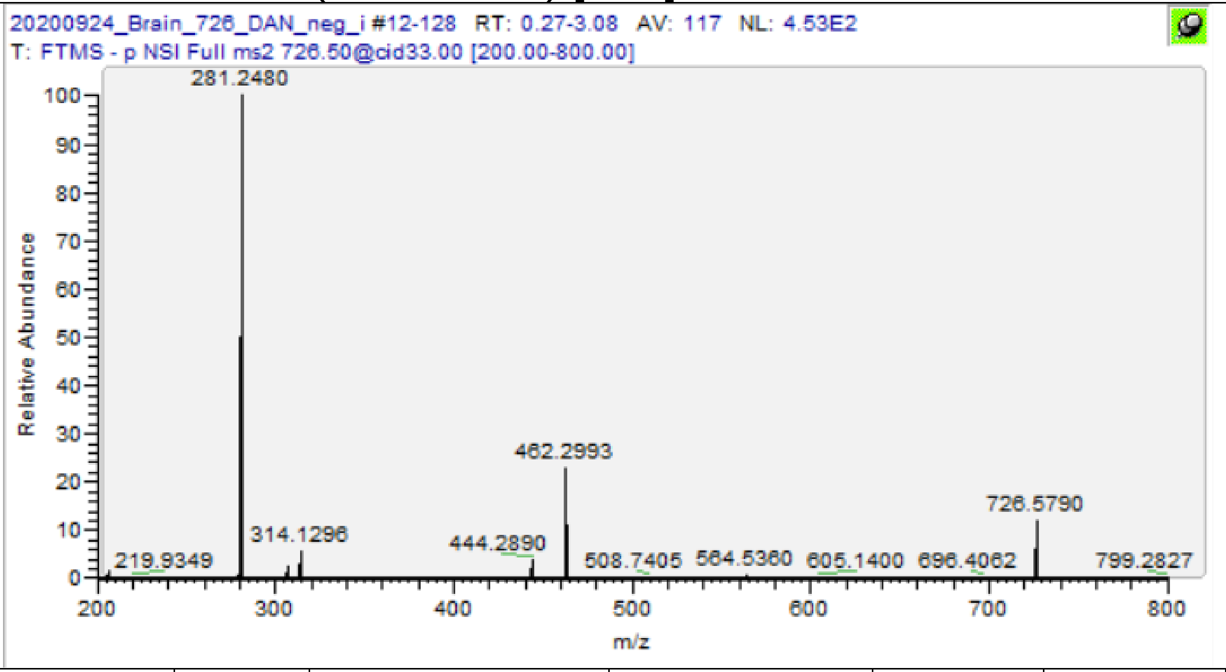

m/z	[I]	Species	Formula	Da	ppm
281.2480	100	FA(18:1)	C18H33O2	281.2486	2.13
444.2890	3.88	PE(P-18:1) -H2O	C23H43O5NP	444.2884	-1.28
462.2992	22.03	PE(P-18:1)	C23H45O6NP	462.299	-0.43
**726.5427**	**12.21**	**PE(P-18:1/18:1)**	**C41H77O7NP**	**726.5443**	**2.22**
564.5361	0.82	Cer 36:1;O2	C36H70NO3	564.5361	0.00
726.5789	13.17	HexCer 36:1;O2	C42H80NO8	726.5889	13.76

PE(P-18:0/18:1) [M-H]- m/z 728.56
PE(P-16:0/20:1) [M-H]- m/z 728.56

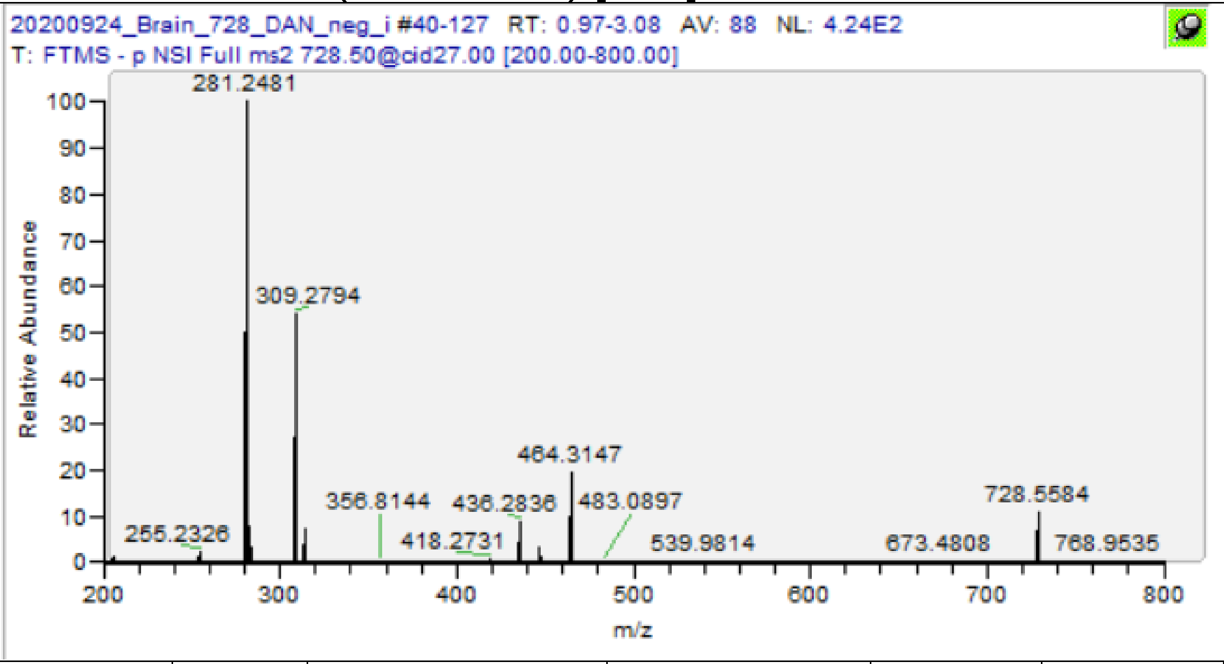

m/z	[I]	Species	Formula	Da	ppm
281.2481	100	FA(18:1)	C18H33O2	281.2486	1.78
309.2794	55.94	FA 20:1	C20H37O2	309.2799	1.62
418.2731	0.76	PE(P-16:0) -H2O	C21H41O5NP	418.2728	-0.77
436.2837	9.07	PE(P-16:0)	C21H43O6NP	436.2834	-0.80
446.3046	3.18	PE(P-18:0) -H2O	C23H45O5NP	446.3041	-1.17
464.3147	20.12	PE(P-18:0)	C23H47O6NP	464.3147	-0.11
**728.5584**	**9.35**	**PE(P-18:0/18:1)/ PE(P-16:0/20:1)**	**C41H79O7NP**	**728.56**	**2.14**

PE(18:1/18:1) [M-H]- m/z 742.5392

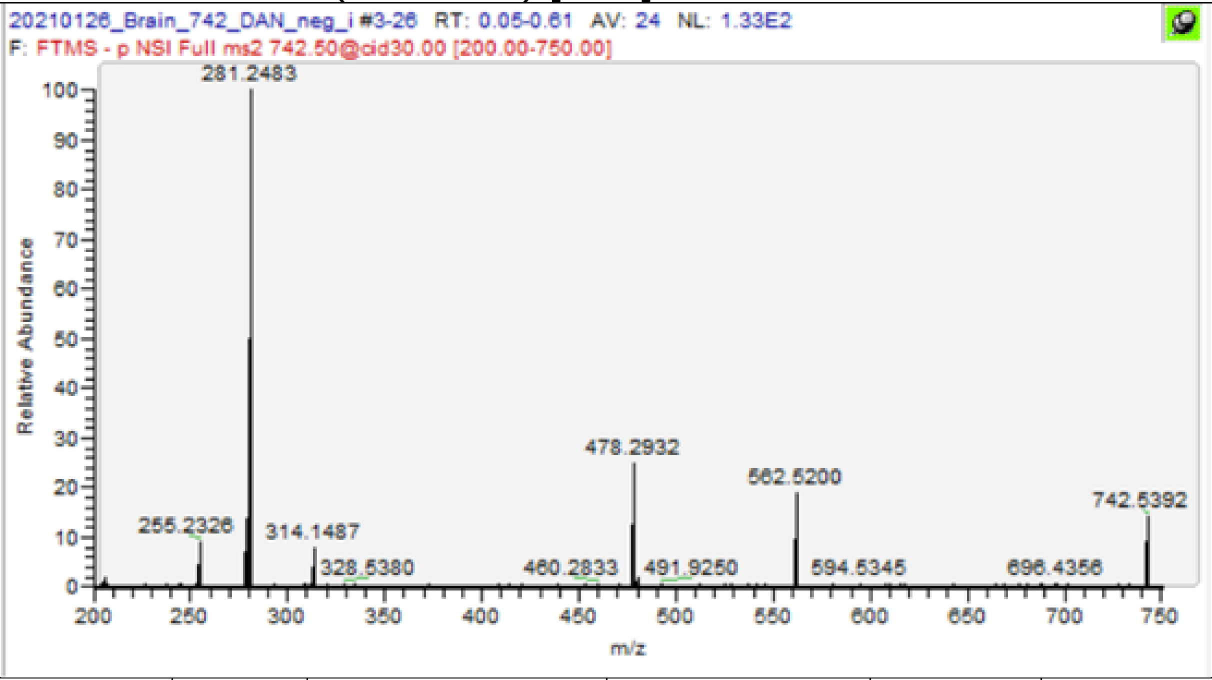

m/z	[I]	Species	Formula	Da	ppm
281.2483	100	FA 18:1	C18H33O2	281.2486	1.06
460.2833	0.89	PE(18:1) -OH	C23H43O6NP	460.2834	0.10
478.2933	25.8	PC(18:1)	C23H45O7NP	478.2939	1.27
**742.5391**	**13.64**	**PE(18:1/18:1)**	**C41H77O8NP**	**742.5392**	**0.17**

PC(16:0/18:1) [M-CH3]- m/z 744.5549

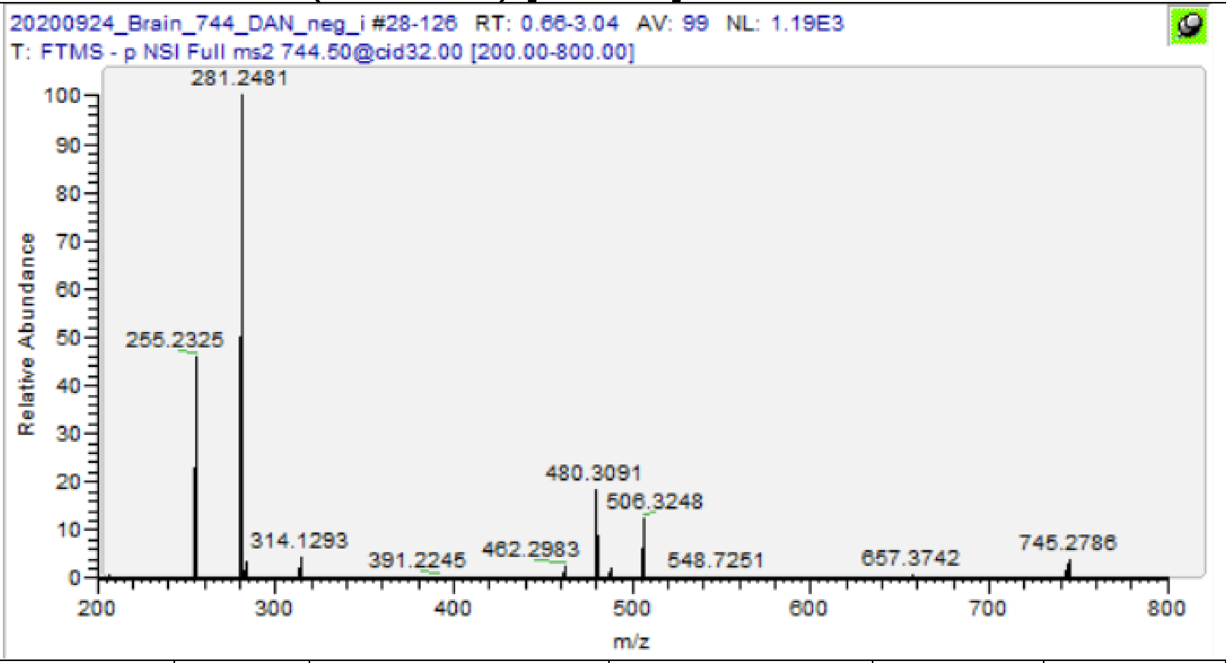

m/z	[I]	Species	Formula	Da	ppm
255.2325	46.82	FA(16:0)	C16H31O2	255.233	1.76
281.2481	100	FA(18:1)	C18H33O2	281.2486	1.78
462.2983	2.58	PC(16:0) -CH3 -OH	C23H45O6NP	462.299	1.51
480.3091	18.72	PC(16:0) -CH3	C23H47O7NP	480.3096	0.96
488.3138	2.05	PC(18:1) -CH3 -H2O	C25H47O6NP	488.3147	1.74
506.3247	12.55	PC(18:1) -CH3	C25H49O7NP	506.3252	1.01
**744.5535**	**12.55**	**PC(16:0/18:1)-CH3**	**C41H79O8NP**	**744.5549**	**1.85**

PA(18:0/22:6) [M-H]- m/z 747.497

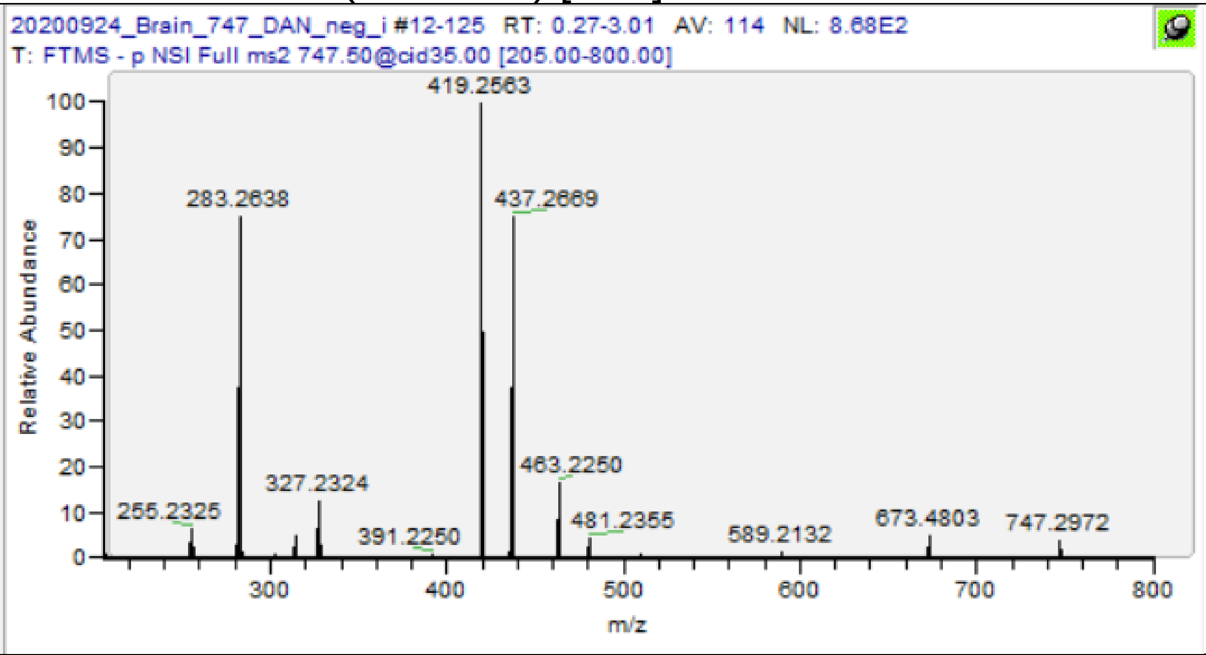

m/z	[I]	Species	Formula	Da	ppm
283.2427	4.9	FA(22:6)-CO2	C21H31	283.2431	1.48
283.2638	72.41	FA(18:0)	C18H35O2	283.2643	1.59
327.2324	12.09	FA 22:6	C22H31O2	327.233	1.68
419.2563	100	PA(18:0) -H2O	C21H40O6P	419.2568	1.19
437.2668	77.11	PA(18:0)	C21H42O7P	437.2674	1.28
463.2250	16.25	PA(22:6) -H2O	C25H36O6P	463.2255	1.08
481.2355	4.1	PA(22:6)	C25H38O7P	481.2361	1.16
**747.4959**	**30**	**PA(18:0/22:6)**	**C43H72O8P**	**747.497**	**1.51**
255.2325	6.05	FA(16:0)	C16H31O2	255.233	1.76
281.2481	6.03	FA(18:1)	C18H33O2	281.2486	1.78
391.2250	0.88	PA(16:0) -H2O	C19H36O6P	391.2255	1.28
465.2620	0.15	PG(16:0)-H2O	C22H42O8P	465.2623	0.60
483.2720	0.26	PG(16:0)	C22H44O9P	483.2728	1.74
673.4803	4.61	PA(18:1/16:0)	C37H70O8P	673.4814	1.60
747.5150	4.5	PG(18:1/16:0)	C40H76O10P	747.5182	4.30

PE(P-18:1/20:1) [M-H]- m/z 754.5756

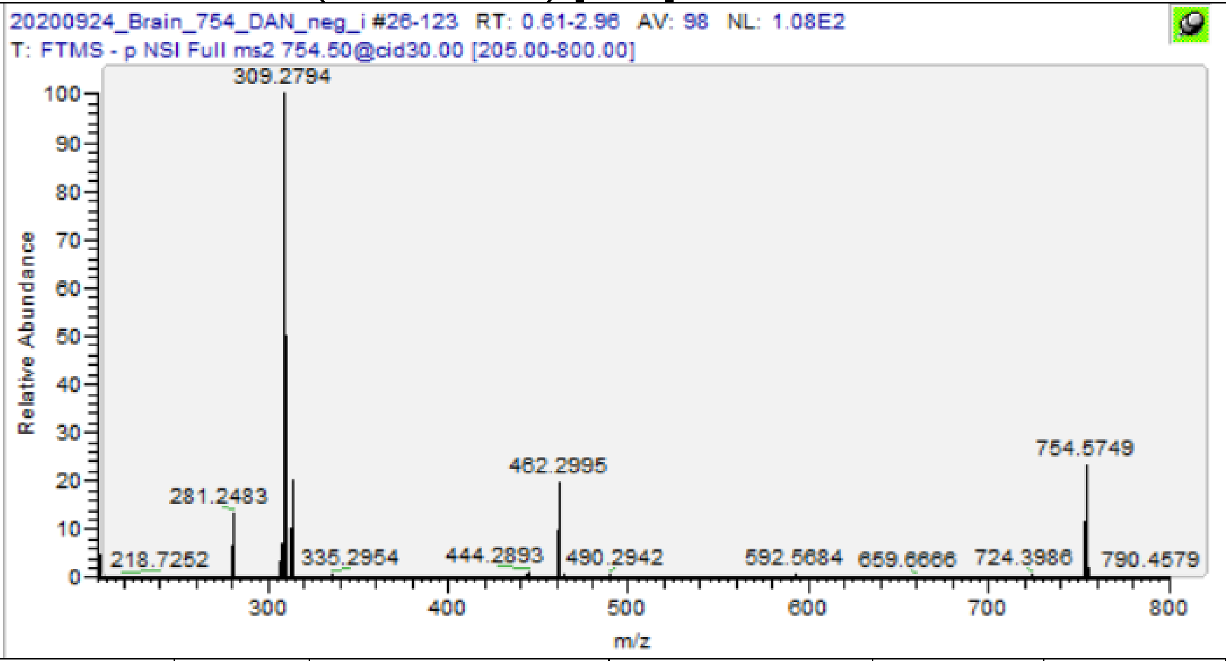

m/z	[I]	Species	Formula	Da	ppm
309.2794	100	FA 20:1	C20H37O2	309.2799	1.62
444.2892	0.6	PE(P-18:1) -H2O	C23H43O5NP	444.2884	-1.73
462.2995	18.2	PE(P-18:1)	C23H45O6NP	462.299	-1.08
**754.5749**	**25.72**	**PE(P-18:1/20:1)**	**C43H81O7NP**	**754.5756**	**0.94**

PE(18:0/20:4) [M-H]- m/z 766.5392

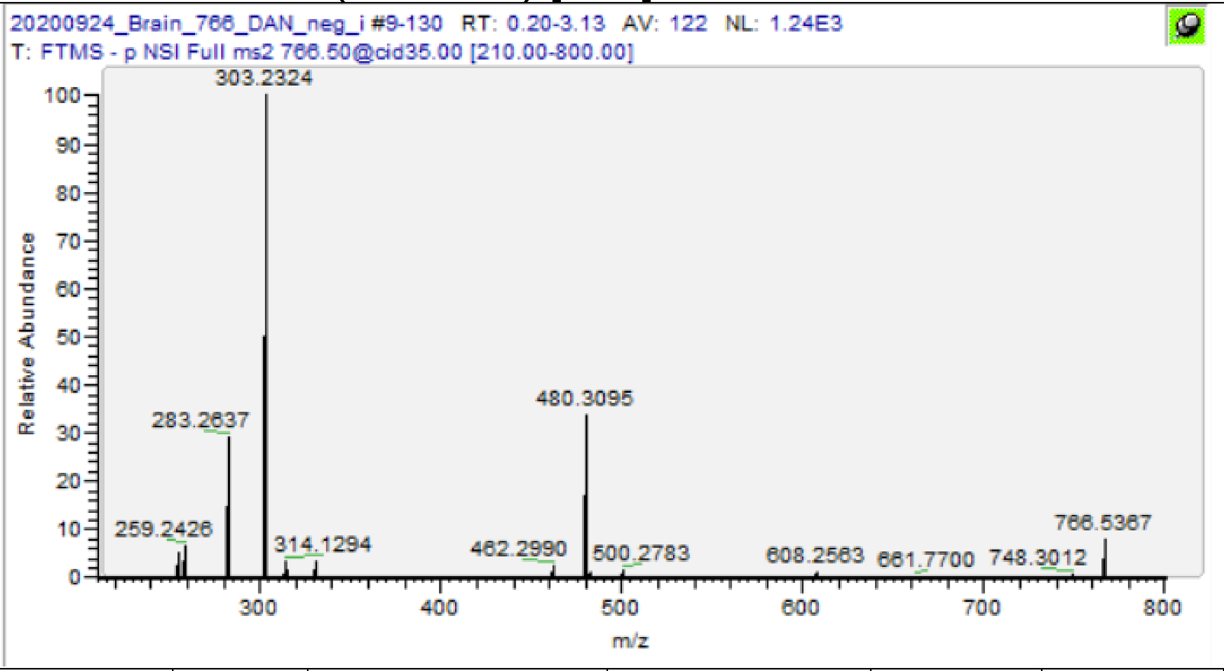

m/z	[I]	Species	Formula	Da	ppm
259.2427	6.18	FA(20:4)-CO2	C19H31	259.2431	1.62
283.2637	29.2	FA(18:0)	C18H35O2	283.2643	1.94
303.2325	100	FA(20:4)	C20H31O2	303.233	1.48
462.2990	2.58	PE(18:0)-H2O	C23H45O6NP	462.299	0.00
480.3095	33.25	PE(18:0)	C23H47O7NP	480.3096	0.12
482.2676	1.09	PE(20:4)-H2O	C25H41O6NP	482.2677	0.21
500.2784	1.71	PE(20:4)	C25H43O7NP	500.2783	-0.28
**766.5373**	**10.69**	**PE(18:0/20:4)**	**C43H77O8NP**	**766.5392**	**2.52**
255.2325	4.9	FA(16:0)	C16H31O2	255.233	1.76
331.2638	3.38	FA 22:4	C22H35O2	331.2643	1.36
452.2781	0.39	PE(16:0)	C21H43O7NP	452.2783	0.35
528.3091	0.22	PE 22:4	C27H47O7NP	528.3096	0.87
766.5373	10.69	PE(16:0/22:4)	C43H77O8NP	766.5392	2.52

SM(d18:1/22:0) [M-CH3]- m/z 771.6386

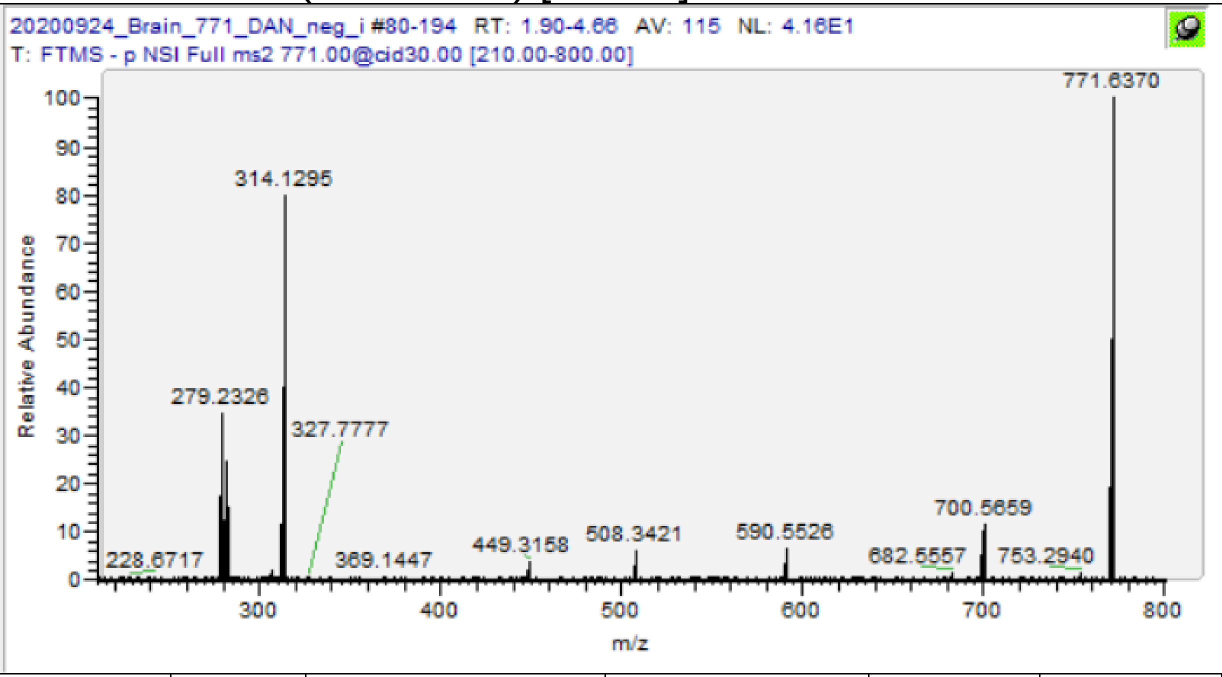

m/z	[I]	Species	Formula	Da	ppm
449.3158	3.53	LSM 18:1;O2 -CH3	C22H46N2O5P	449.31498	-1.83
682.5557	1.65	CerP 40:1;O2 -H2O	C40H79O6NP	682.55448	-1.79
700.5659	11.84	CerP 40:1;O2	C40H79O6NP	700.56505	-1.21
**771.637**	**100**	**SM(d18:1/22:0) -CH3**	**C44H88O6N2P**	**771.63855**	**2.01**

PE(P-18:0/22:6) -H [M-H]- m/z 774.5443

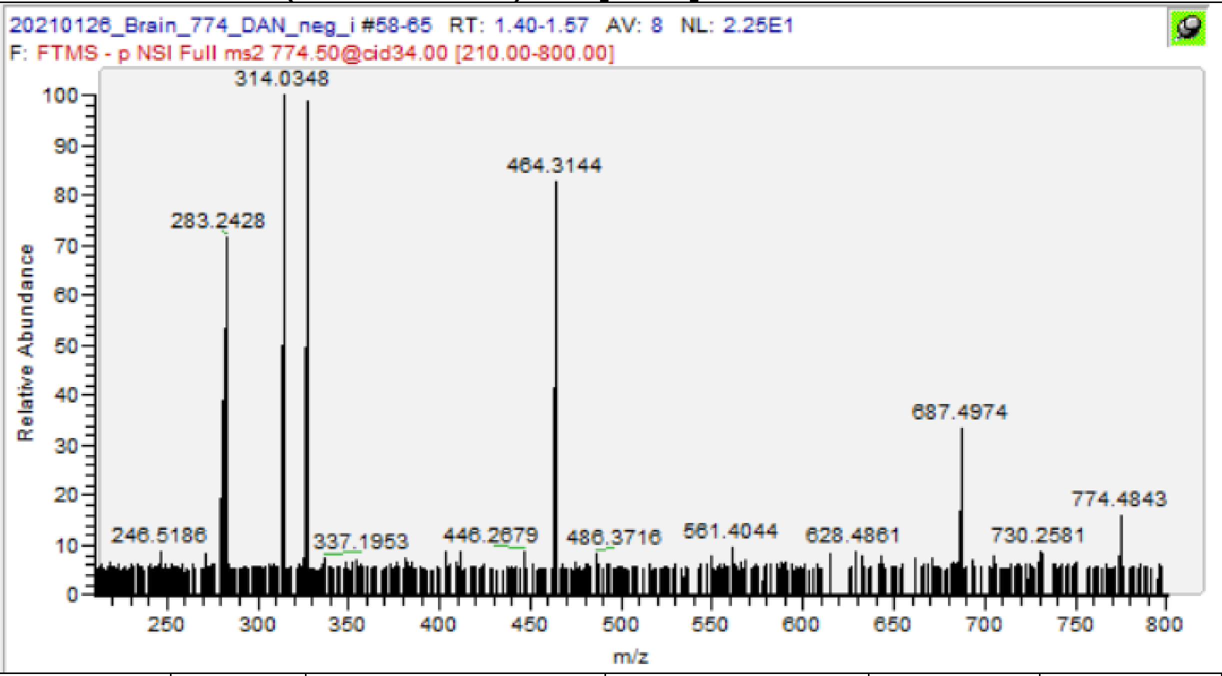

m/z	[I]	Species	Formula	Da	ppm
283.2426	48.86	FA 22:6 -CO2	C21H31	283.2431	1.84
327.2325	100	FA 22:6	C22H31O2	327.233	1.38
446.3045	11.79	PE(P-18:0) -CH3 -H2O	C23H45O5NP	446.3041	-0.94
464.3148	85.87	PE(P-18:0)	C23H47O6NP	464.3147	-0.32
**774.5427**	**100**	**PE(P-18:0/22:6) -H**	**C45H77O7NP**	**774.5443**	**2.08**

PE(18:0/20:4(OH)) [M-H]- m/z 782.5341

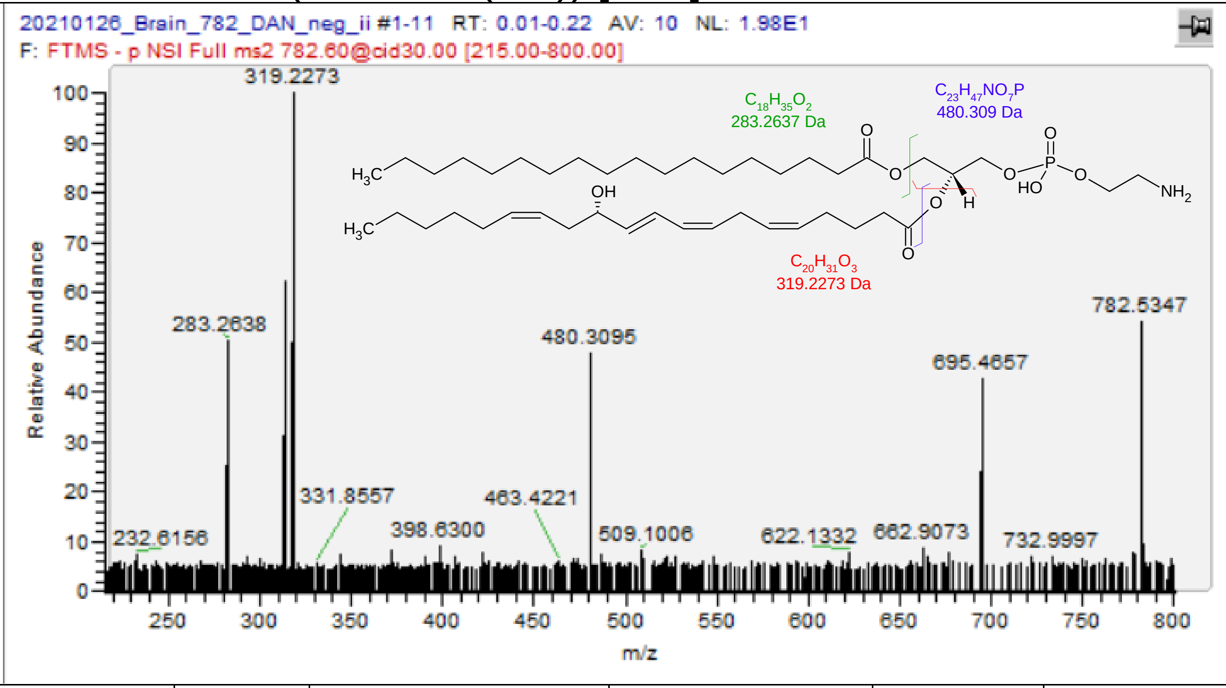

m/z	[I]	Species	Formula	Da	ppm
283.2638	49.38	FA 18:0	C18H35O2	283.2643	1.59
319.2273	100	FA(20:4(OH)	C20H31O3	319.2279	1.79
480.3095	46.83	PE(18:0)	C23H47O7NP	480.3096	0.12
**782.5347**	**53.53**	**PE(18:0/20:4(OH)**	**C43H77O9NP**	**782.5341**	**-0.72**

PC(16:1/22:6) [M-CH3]- m/z 788.5236
PS(18:1/18:0) [M-H]- m/z 788.5447

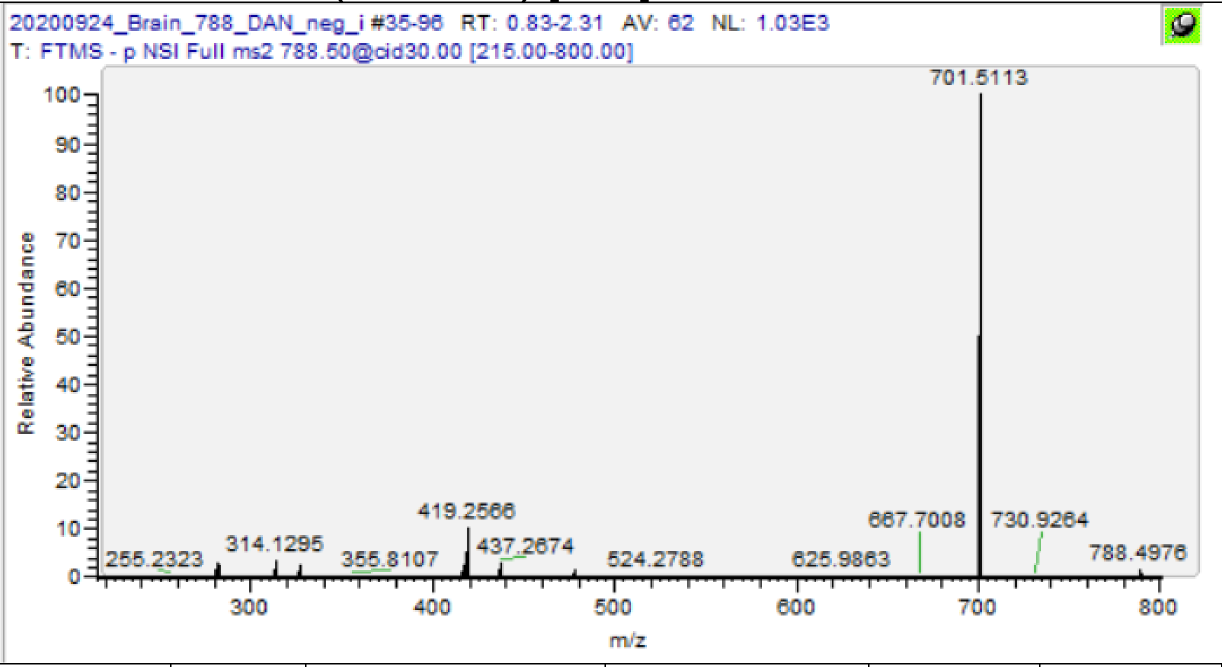

m/z	[I]	Species	Formula	Da	ppm
281.2482	2.96	FA(18:1)	C18H33O2	281.2486	1.42
283.2638	2.78	FA(18:0)	C18H35O2	283.2643	1.59
417.2409	2.74	PA(18:1) -H2O	C21H38O6P	417.2412	0.60
419.2565	10.72	PA(18:0) -H2O	C21H40O6P	419.2568	0.72
435.2516	0.15	PA(18:1)	C21H40O7P	435.2517	0.25
437.2672	3.28	PA(18:0)	C21H42O7P	437.2674	0.37
701.5113	100	PA(18:1/18:0)	C39H74O8P	701.5127	1.97
**788.5447**	**89**	**PS(18:1/18:0)**	**C42H79O10NP**	**788.5447**	**0.01**
283.2426	1.11	FA(22:6)-CO2	C21H31	283.2431	1.84
327.2325	2.63	FA 22:6	C22H31O2	327.233	1.38
478.2939	1.64	PC(16:1)-CH3	C23H45O7NP	478.2939	0.02
**788.5244**	**42.76**	**PC(16:1/22:6)-CH3**	**C45H75O8NP**	**788.5236**	**-1.04**

PE(18:0/22:6) [M-H]- m/z 790.5392

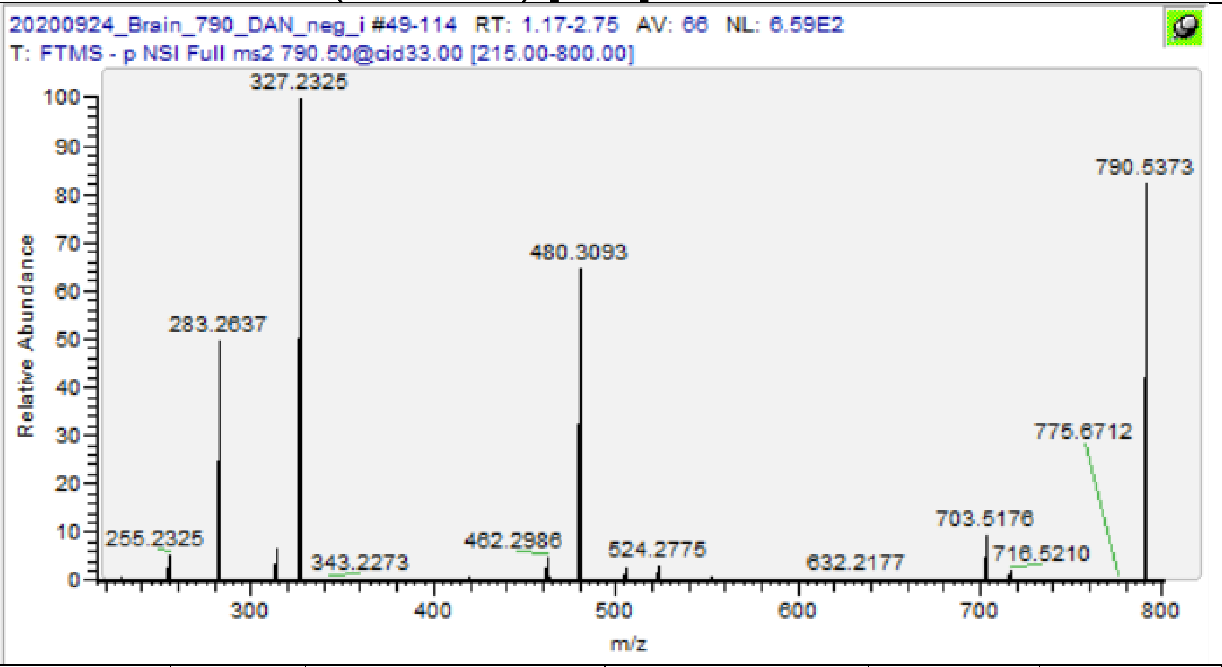

m/z	[I]	Species	Formula	Da	ppm
283.2426	29.82	FA(22:6)-CO2	C21H31	283.2431	1.84
283.2637	33.72	FA(18:0)	C18H35O2	283.2643	1.94
327.2325	68.6	FA 22:6	C22H31O2	327.233	1.38
419.2562	0.36	PA(18:0) -H2O	C21H40O6P	419.2568	1.43
462.2986	3.17	PE(18:0) -H2O	C23H45O6NP	462.299	0.87
480.3093	43.06	PE(18:0)	C23H47O7NP	480.3096	0.54
506.2671	1.7	PE(22:6) -H2O	C27H41O6NP	506.2677	1.19
524.2776	2.17	PE(22:6)	C27H43O7NP	524.2783	1.26
**790.5374**	**100**	**PE(18:0/22:6)**	**C45H77O8NP**	**790.5392**	**2.31**

HexCer(d18:1/22:0(2OH)) [M-H]- m/z 798.6465

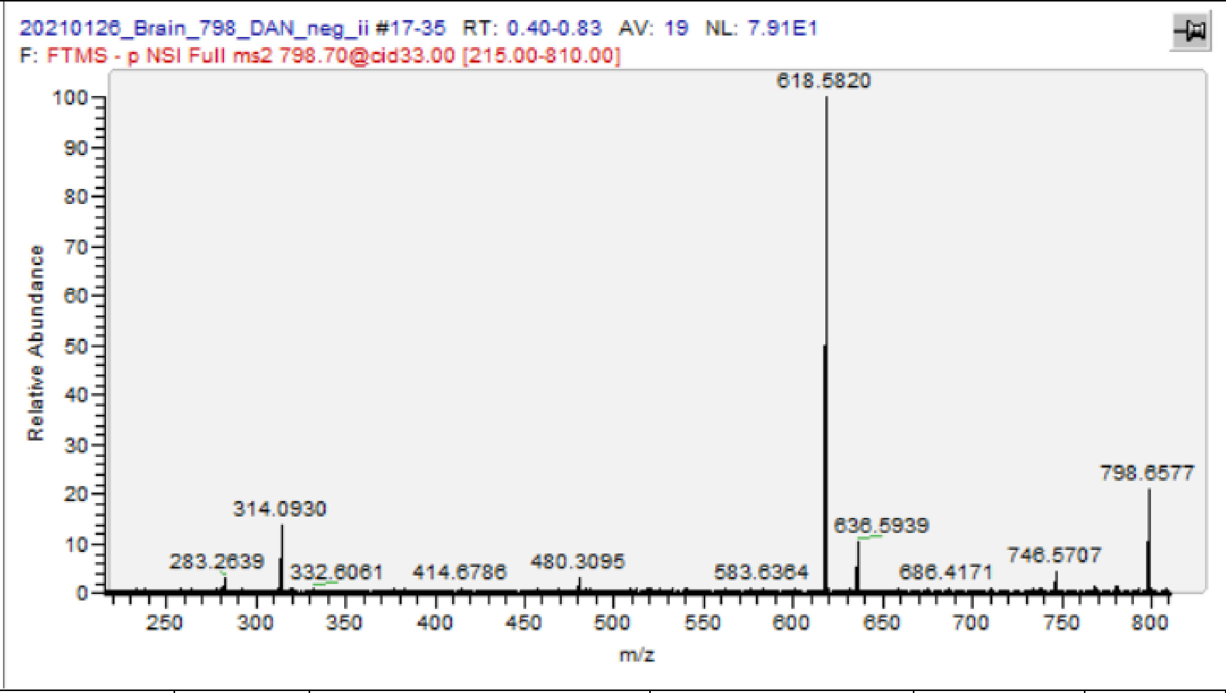

m/z	[I]	Species	Formula	Da	ppm
618.5819	100	Cer(d40:1(2OH)) -H2O	C40H76O3N	618.5831	1.89
636.594	10.49	Cer(d40:1(2OH))	C40H78O4N	636.5936	-0.58
780.6371	1.07	HexCer(d40:1(2OH)) -H2O	C46H86O8N	780.6359	-1.55
**798.6443**	**100**	**HexCer(d18:1/22:0(2OH))**	**C46H88O9N-**	**798.6465**	**2.70**

C18:1-Sulf [M-H]- m/z 806.5458

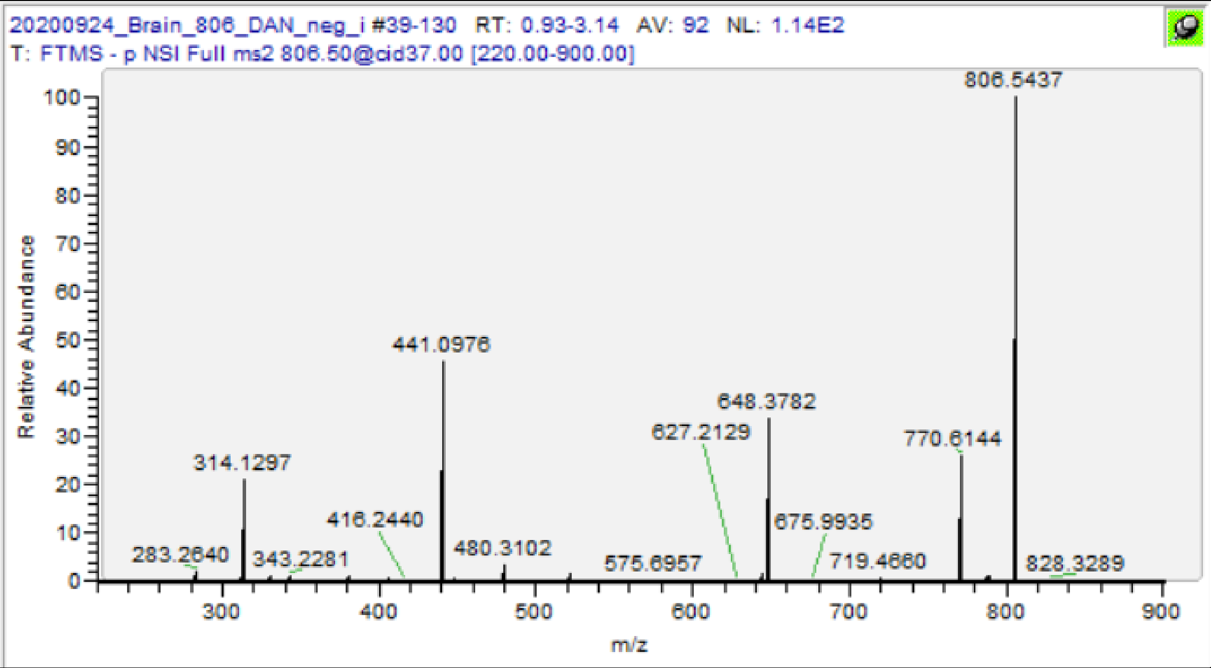

m/z	[I]	Species	Formula	Da	ppm
522.2748	1.82	C18:1 Sulf. -C18 -H2O	C24H44O9NS	522.2742	-1.09
788.5358	0.92	C18:1 Sulf. -H2O	C42H78O10NS	788.5352	-0.77
**806.5436**	**100**	**C18:1 Sulf**	**C42H80O11NS**	**806.5458**	**2.68**

C18(OH)-Sulf [M-H]- m/z 822.5407

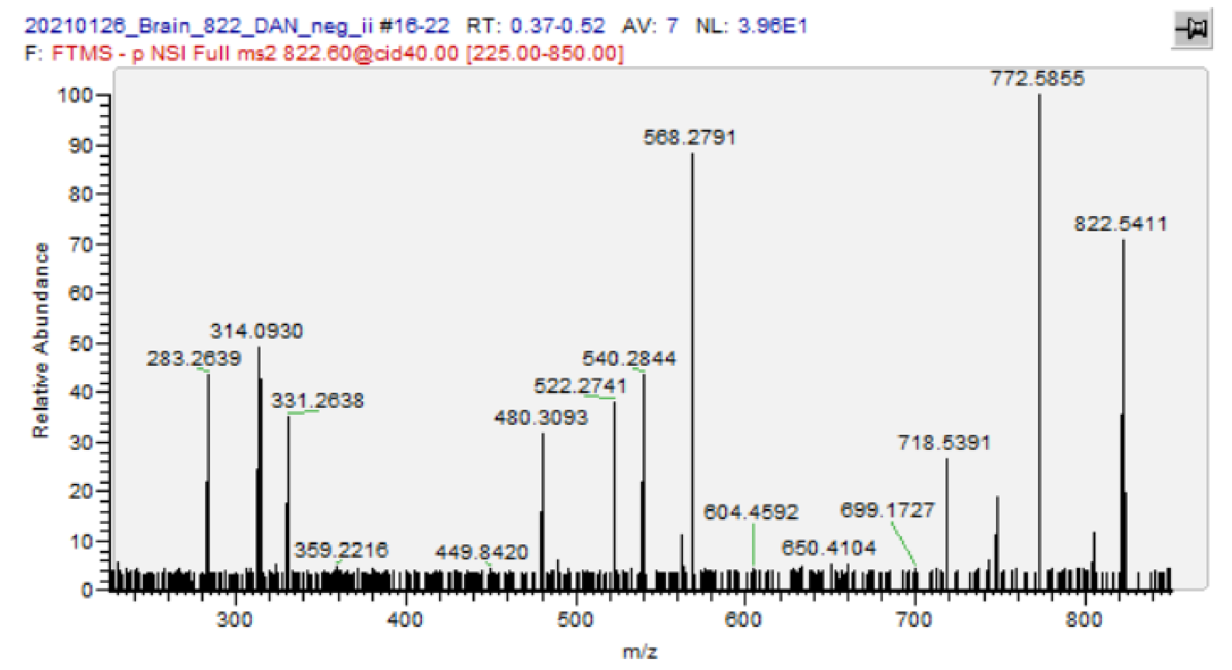

m/z	[I]	Species	Formula	Da	ppm
522.2741	38.2	C18(OH) Sulf. -C18 -H2O	C24H44O9NS	522.2742	0.25
540.2844	45.03	C18(OH) Sulf. -C18	C24H46O10NS	540.2848	0.72
568.2791	89.36	C18(OH) Sulf. -C17	C25H46O11NS	568.2797	1.07
**822.5411**	**70.85**	**C18(OH) Sulf**	**C42H80O12NS**	**822.5407**	**-0.52**

HexCer(d18:1/24:0(2OH)) [M-H]- m/z 826.6778

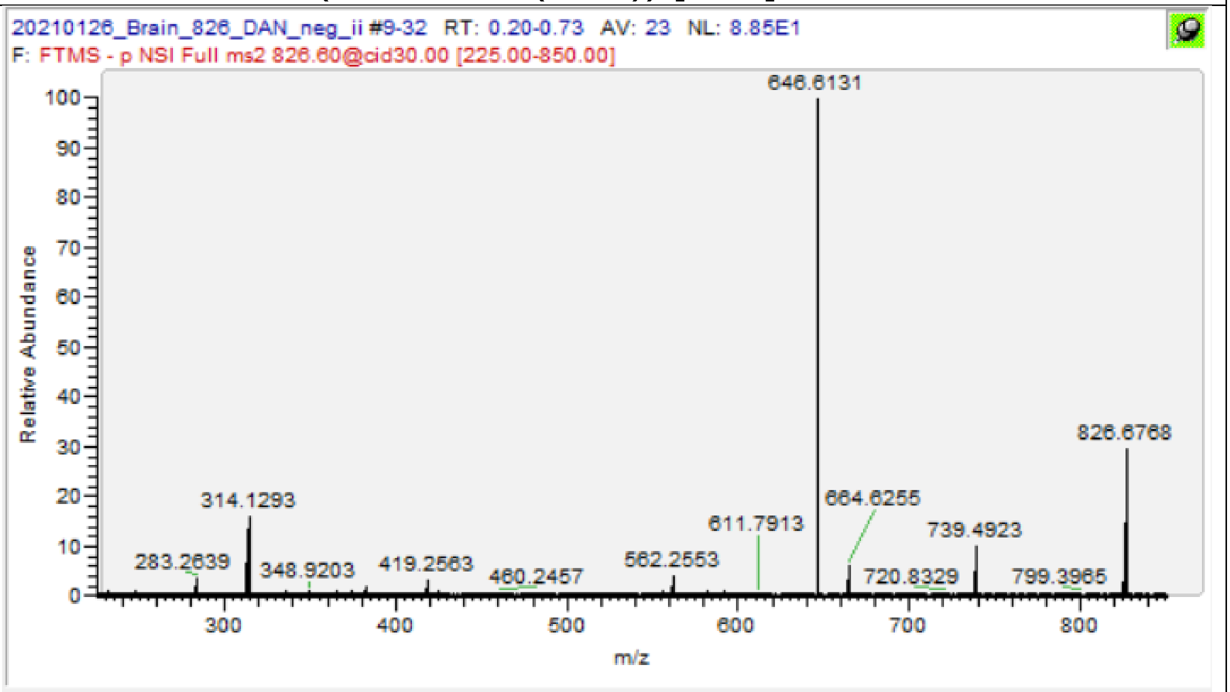

m/z	[I]	Species	Formula	Da	ppm
383.3528	2.32	FA 24:0;O	C24H47O3	383.3531	0.70
646.613	100	Cer(d18:1/24:0(2OH)) -H2O	C42H80O3N	646.6144	2.12
664.6218	8.67	Cer(d18:1/24:0(2OH))	C42H82O4N	664.6249	4.71
**826.6757**	**59.34**	**HexCer(d18:1/24:0(2OH))**	**C48H92O9N**	**826.6778**	**2.49**

PS(18:0/22:6) [M-H]- m/z 834.5291

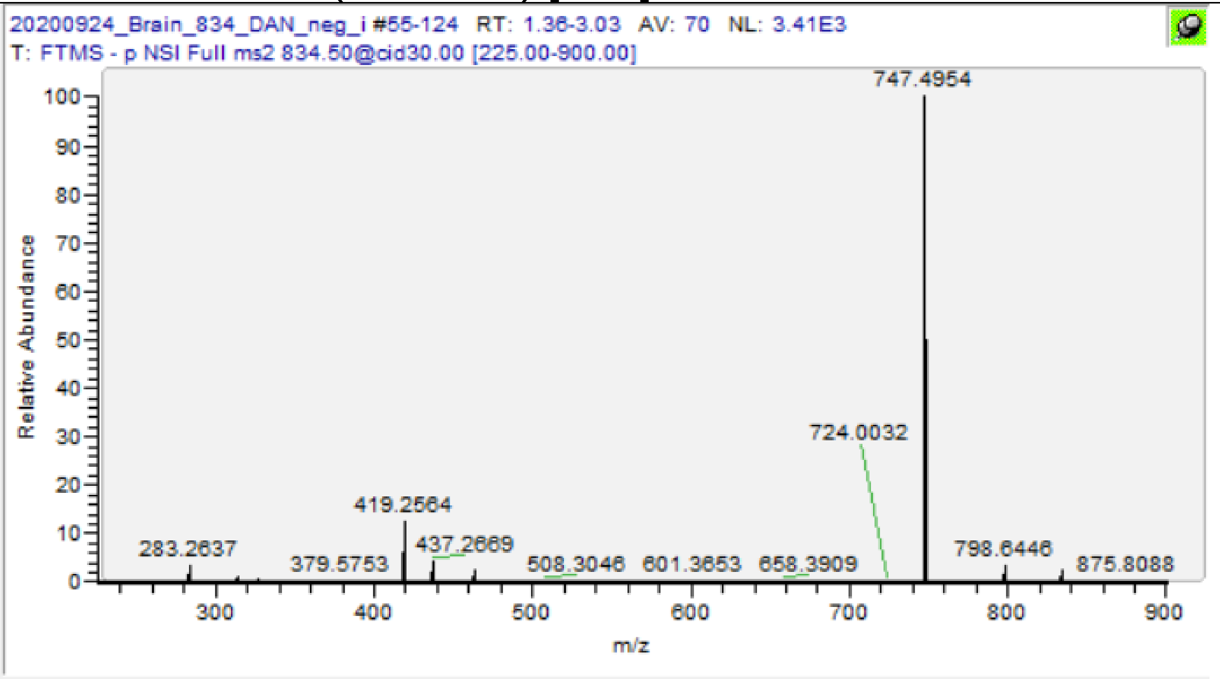

m/z	[I]	Species	Formula	Da	ppm
283.2427	0.1	FA(22:6)-CO2	C21H31	283.2431	1.48
283.2637	3.75	FA (18:0)	C18H35O2	283.2643	1.94
327.2325	0.68	FA (22:6)	C22H31O2	327.233	1.38
419.2563	13.12	PA(18:0) -H2O	C21H40O6P	419.2568	1.19
437.2669	4.51	PA(18:0)	C21H42O7P	437.2674	1.05
463.2251	2.75	PA(22:6) -H2O	C25H36O6P	463.2255	0.86
481.236	0.22	PA(22:6)	C25H38O7P	481.2361	0.12
747.4954	100	PA(18:0/22:6)	C43H72O8P	747.497	2.18
**834.5274**	**78**	**PS(18:0/22:6)**	**C46H77O10NP**	**834.5291**	**1.99**

PS(18:1/22:0) [M-H]- m/z 844.6073

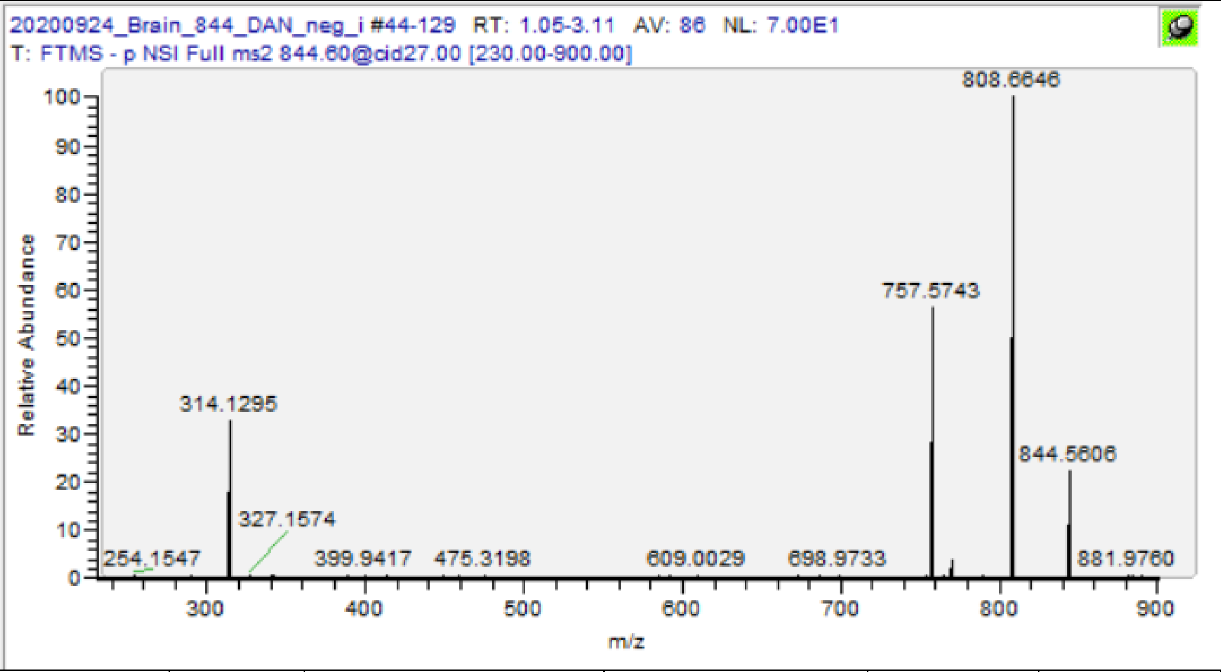

m/z	[I]	Species	Formula	Da	ppm
475.3198	0.57	PA(22:0) -H2O	C25H48O6P	475.3194	-0.84
757.5743	56.73	PA(18:1/22:0)	C43H82O8P	757.5753	1.29
**844.6081**	**10.6**	**PS(18:1/22:0)**	**C46H87O10NP**	**844.6073**	**-0.93**

PI(16:0/20:4) [M-H]- m/z 857.5186

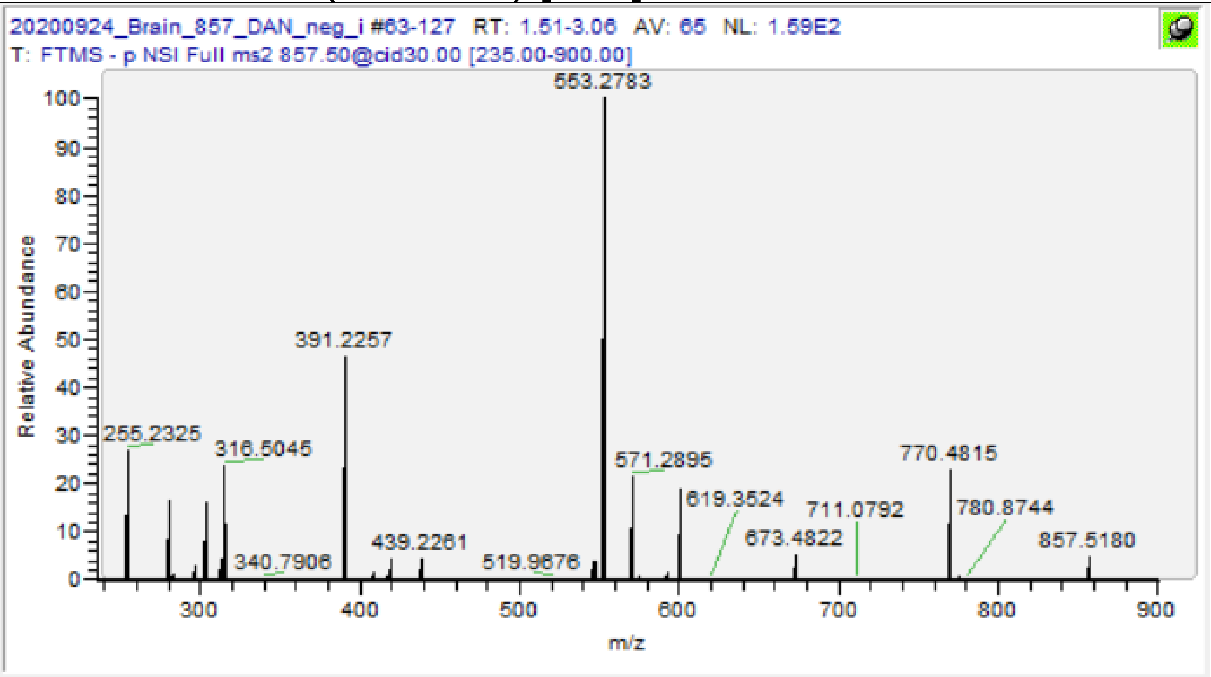

m/z	[I]	Species	Formula	Da	ppm
255.2325	26.3	FA(16:0)	C16H31O2	255.233	1.76
303.2328	16.04	FA(20:4)	C20H31O2	303.233	0.49
391.2256	47.15	PA(16:0) -H2O	C19H36O6P	391.2255	-0.26
409.2363	1.67	PA(16:0)	C19H38O7P	409.2361	-0.59
439.2260	4.16	PA(20:4) -H2O	C23H36O6P	439.2255	-1.14
553.2784	100	PI(16:0)-H2O	C25H46O11P	553.2783	-0.14
571.2896	21.69	PI(16:0)	C25H48O12P	571.2889	-1.24
601.2789	18.69	PI(20:4)-H2O	C29H46O11P	601.2783	-0.96
**857.5180**	**4.63**	**PI(16:0/20:4)**	**C45H78O13P**	**857.5186**	**0.64**

C22-Sulf. [M-H]- m/z 862.6084

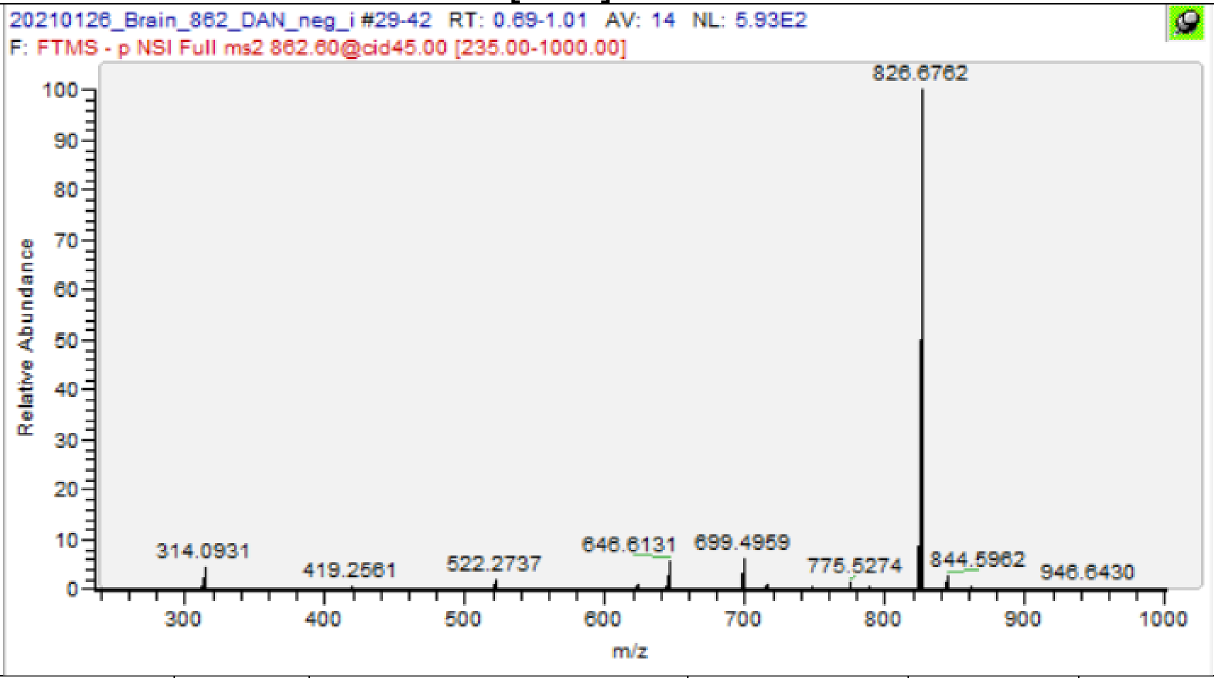

m/z	[I]	Species	Formula	Da	ppm
522.2736	1.8	C22 Sulf. -C22 -H2O	C24H44O9NS	522.2742	1.21
646.6132	5.69	Cer(d18:1/24:0(2OH)) -H2O	C42H80O3N	646.6144	1.81
699.496	5.81	PA(18:0/18:2)	C39H72O8P	699.497	1.47
826.6761	100	HexCer(d18:1/24:0(2OH))	C48H92O9N	826.6778	2.01
**862.6062**	**0.24**	**C22 Sulf.**	**C46H88O11NS**	**862.6084**	**2.50**

PI(18:0/18:1) [M-H]- m/z 863.5655

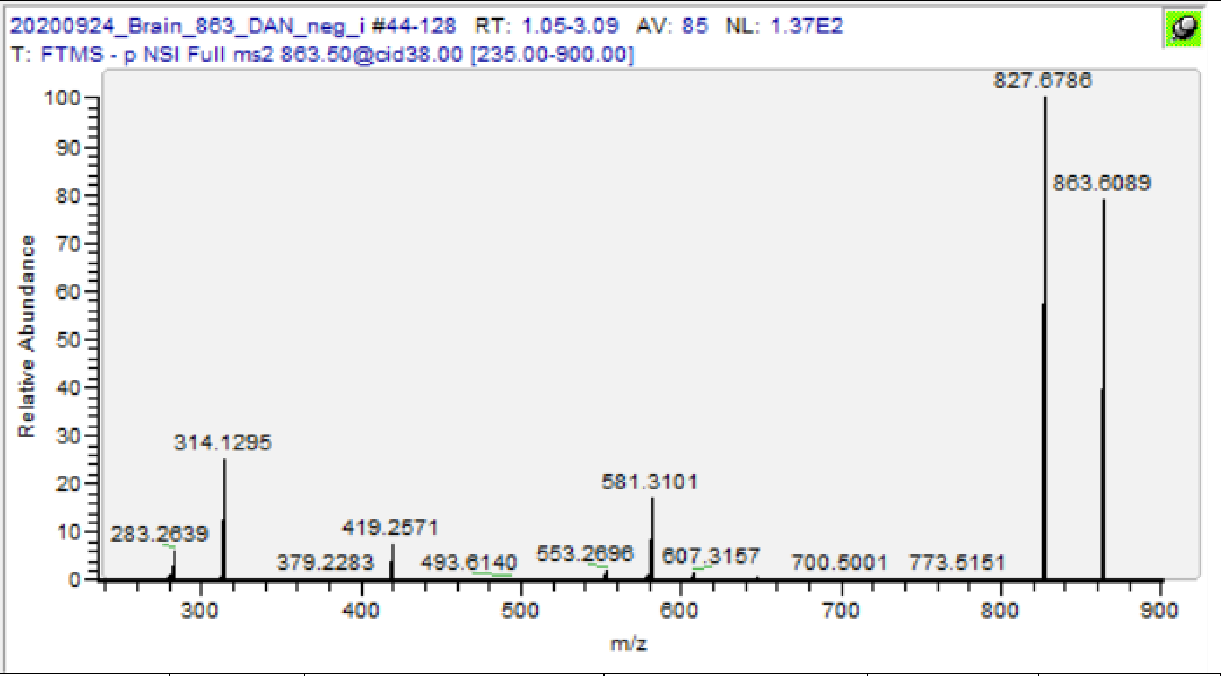

m/z	[I]	Species	Formula	Da	ppm
281.2484	1.07	FA(18:1)	C18H33O2	281.2486	0.71
283.2639	6.62	FA(18:0)	C18H35O2	283.2643	1.24
417.2421	0.37	PA(18:1) -H2O	C21H38O6P	417.2412	-2.28
419.2572	7.27	PA(18:0) -H2O	C21H40O6P	419.2568	-0.95
579.2947	1.33	PI(18:0)-H2O	C27H48O11P	579.294	-1.26
581.3101	16.6	PI(18:0)-H2O	C27H50O11P	581.3096	-0.83
**863.5636**	**56**	**PI(18:0/18:1)**	**C45H84O13P**	**863.5655**	**2.20**

PS(18:1/24:0) [M-H]- m/z 872.6386

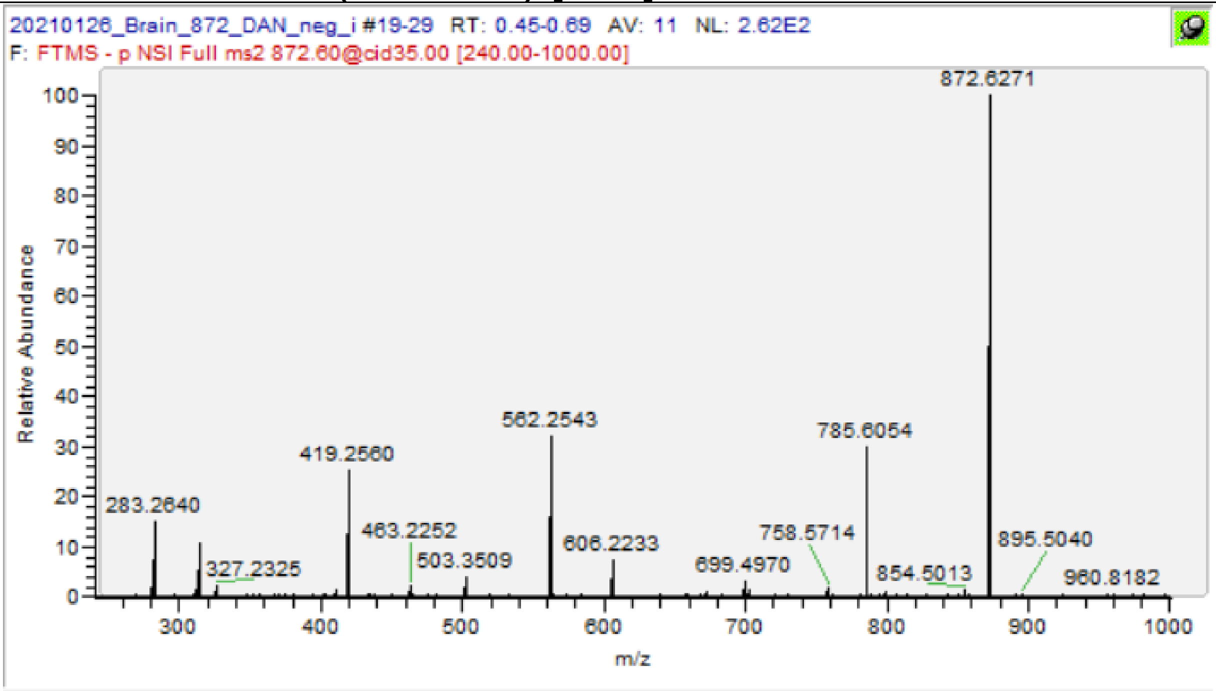

m/z	[I]	Species	Formula	Da	ppm
281.2482	7.12	FA 18:1	C18H33O2	281.2486	1.42
283.264	32.11	FA 18:0	C18H35O2	283.2643	0.88
367.3576	2.78	FA 24:0	C24H47O2	367.3582	1.49
785.6053	83.61	PA(18:1/24:0)	C45H86O8P	785.6066	1.62
**872.6341**	**1.93**	**PS(18:1/24:0)**	**C48H91O10NP**	**872.6386**	**5.12**

C22(OH)-Sulf [M-H]- m/z 878.6033

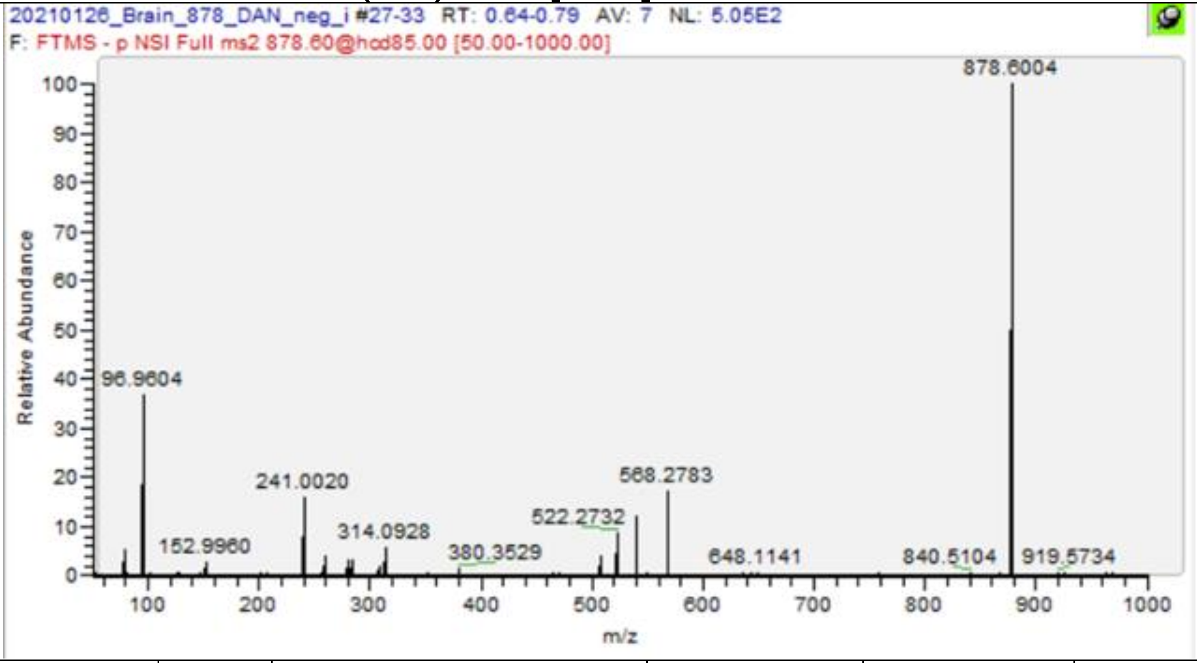

m/z	[I]	Species	Formula	Da	ppm
96.9604	32.7	Sulfate	HO4S	96.9601	-3.09
241.002	16.54	Sulf-Hg	C6H9O8S	241.0024	1.49
507.2641	10.03	C22(OH) Sulf. -C22 -N -H2O	C24H43O9S	507.2633	-1.52
522.2745	27.51	C22(OH) Sulf. -C22 -H2O	C24H44O9NS	522.2742	-0.52
540.2849	28.72	C22(OH) Sulf. -C22	C24H46O10NS	540.2848	-0.20
550.2695	1.09	C22(OH) Sulf. -C21 -H2O	C25H44O10NS	550.2691	-0.65
568.2793	81.45	C22(OH) Sulf. -C21	C25H46O11NS	568.2797	0.72
618.5836	3.13	C22(OH) Sulf. -Gala -H2O	C40H76O3N	618.5831	-0.86
636.5944	0.44	C22(OH) Sulf. -Gala	C40H78O4N	636.5936	-1.21
860.5915	13.47	C22(OH) Sulf. -H2O	C46H86O11NS	860.5927	1.41
**878.6008**	**100**	**C22(OH) Sulf**	**C46H88O12NS**	**878.6033**	**2.81**

PI(18:1/20:4) [M-H]- m/z 883.5342

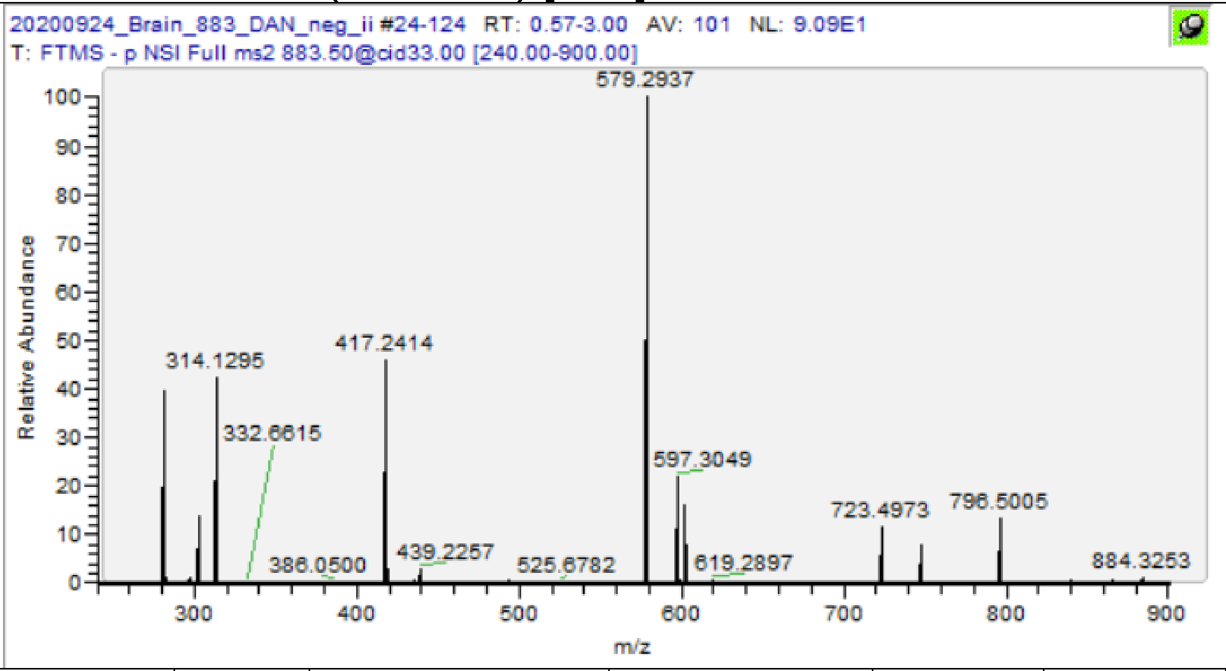

m/z	[I]	Species	Formula	Da	ppm
281.2482	39.3	FA(18:1)	C18H33O2	281.2486	1.42
297.0378	1.15	GPI -2H2O	C9H14O9P	297.0381	0.98
303.2328	13.36	FA(20:4)	C20H31O2	303.233	0.49
417.2414	45.61	PA(18:1) -H2O	C21H38O6P	417.2412	-0.60
435.2524	0.8	PA(18:1)	C21H40O7P	435.2517	-1.59
439.2257	2.98	PA (20:4) -H2O	C23H36O6P	439.2255	-0.46
579.2937	100	PI(18:0)-H2O	C27H48O11P	579.294	0.47
597.3050	21.19	PI(18:1)	C27H50O12P	597.3045	-0.77
601.2789	15.83	PI(20:4)-H2O	C29H46O11P	601.2783	-0.96
619.2897	0.45	PI(20:4)	C29H48O12P	619.2889	-1.31
**883.5321ɣ**	**100**	**PI(18:1/20:4)**	**C47H80O13P**	**883.5342**	**2.38**

ɣ Value from MS1 scan

PI(18:0/20:4) [M-H]- m/z 885.5499

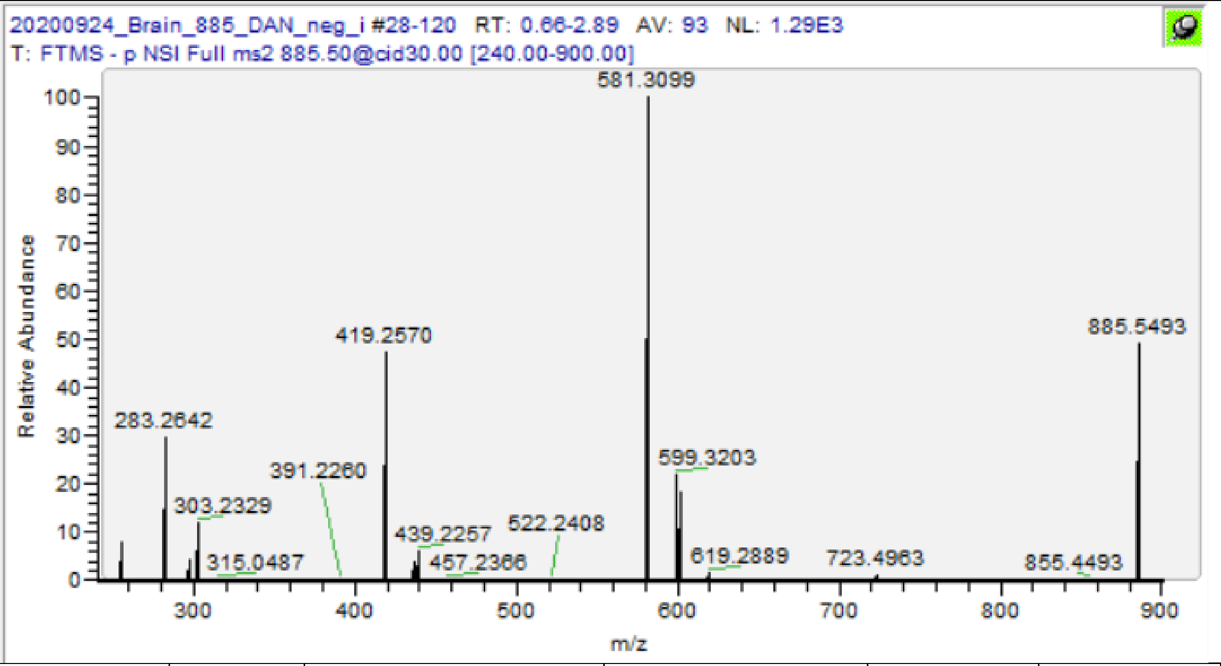

m/z	[I]	Species	Formula	Da	ppm
259.2431	0.36	FA(20:4)-CO2	C19H31	259.2431	0.08
283.2642	30.34	FA(18:0)	C18H35O2	283.2643	0.18
297.0381	4.21	GPI -2H2O	C9H14O9P	297.0381	-0.03
303.2329	12.24	FA(20:4)	C20H31O2	303.233	0.16
315.0487	0.47	GPI	C9H16O10P	315.0487	-0.13
419.2570	47.46	PA(18:0) -H2O	C21H40O6P	419.2568	-0.48
437.2676	3.89	PA(18:0)	C21H42O7P	437.2674	-0.55
439.2257	6.24	PA (20:4) -H2O	C23H36O6P	439.2255	-0.46
581.3098	100	PI(18:0)-H2O	C27H50O11P	581.3096	-0.31
599.3203	22.09	PI(18:0)	C27H52O12P	599.3202	-0.18
601.2785	18.34	PI(20:4)-H2O	C29H46O11P	601.2783	-0.30
619.2888	1.65	PI (20:4)	C29H48O12P	619.2889	0.15
723.4962	1.16	PA(18:0/20:4)	C41H72O8P	723.497	1.15
**885.5493**	**48.69**	**PI(18:0/20:4)**	**C47H82O13P**	**885.5499**	**0.62**

C24:1-Sulf. [M-H]- m/z 888.624

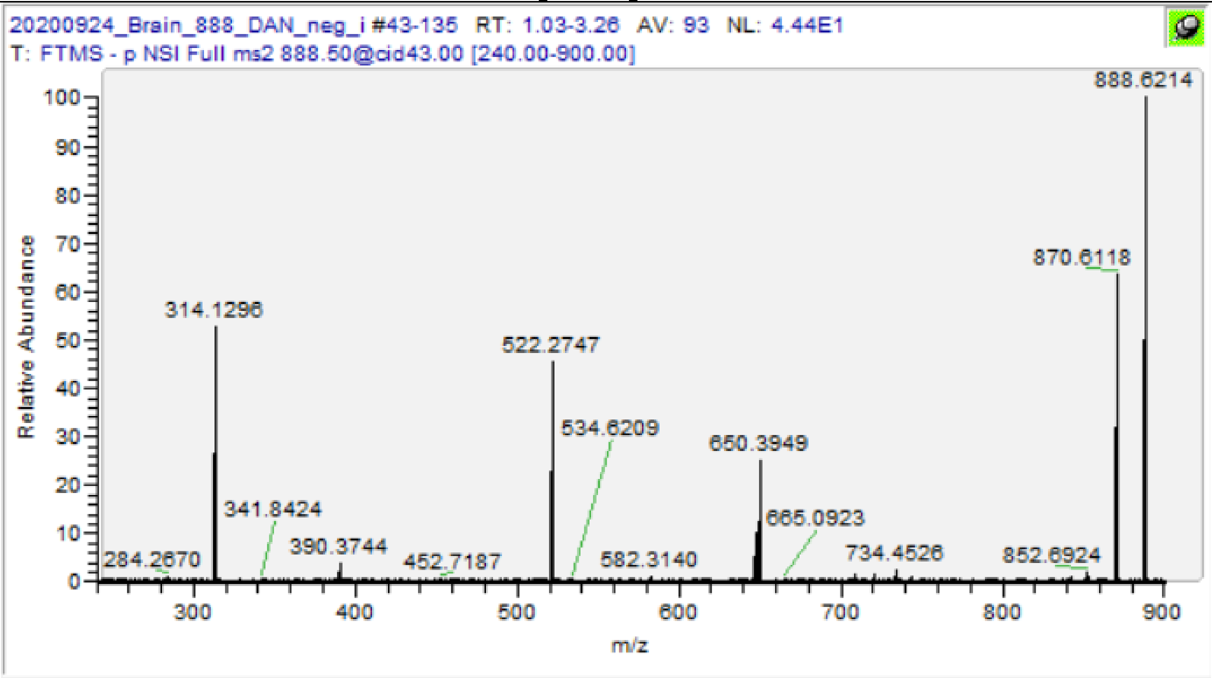

m/z	[I]	Species	Formula	Da	ppm
390.3745	8.84	C24:1 Sulf. -C16 -Gala	C26H48ON	390.3741	-0.92
522.2748	72.85	C24:1 Sulf. -C24 -H2O	C24H44O9NS	522.2742	-1.09
616.6045	1.17	C24:1 Sulf. -Hg	C41H78O2N	616.6038	-1.14
648.3793	18.76	C24:1 Sulf. -C16 -H2	C32H58O10NS	648.3787	-0.94
650.3948	46.85	C24:1 Sulf. -C16	C32H60O10NS	650.3943	-0.71
870.6110	100	C24:1 Sulf. -H2O	C48H88O10NS	870.6134	2.80
**888.6228**	**52.22**	**C24:1 Sulf.**	**C48H90O11NS**	**888.624**	**1.36**

C24-Sulf. [M-H]- m/z 890.6397

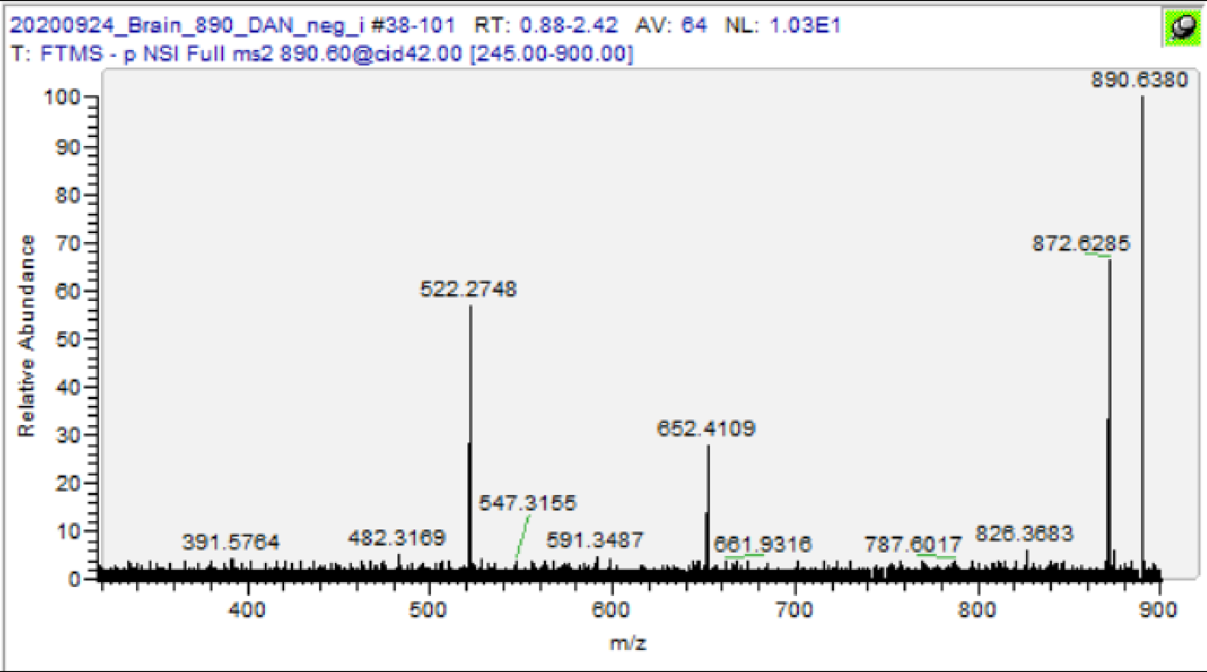

m/z	[I]	Species	Formula	Da	ppm
392.3891	0.3	C24:0 Sulf. -C16 -Gala	C26H50ON	392.3898	1.76
522.2749	3.16	C24:0 Sulf. -C24 -H2O	C24H44O9NS	522.2742	-1.28
650.3949	0.47	C24:0 Sulf. -C16 -H2	C32H60O10NS	650.3943	-0.86
652.4108	0.85	C24:0 Sulf. -C16	C32H62O10NS	652.41	-1.24
872.6276	3.55	C24 Sulf. -OH	C48H90O10NS	872.6291	1.71
**890.6376**	**100**	**C24 Sulf.**	**C48H92O11NS**	**890.6397**	**2.31**

PI(18:0/20:4(OH)) [M-H]- m/z 901.5448

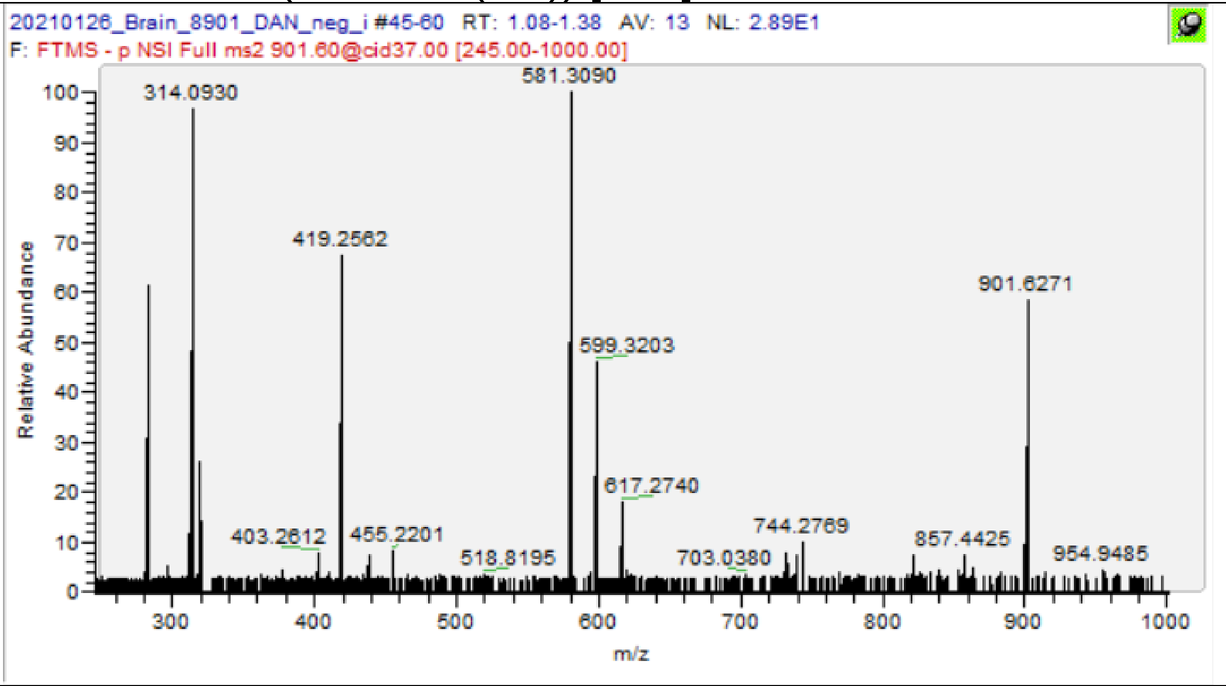

m/z	[I]	Species	Formula	Da	ppm
283.2638	5.06	FA 18:0	C18H35O2	283.2643	1.59
297.0378	5.11	GPI -2H2O	C9H14O9P	297.0381	0.98
319.2274	3.05	FA 20:4(OH) -CO2	C20H31O3	319.2279	1.47
419.2561	9.2	PA(18:0) -H2O	C21H40O6P	419.2568	1.67
437.2666	0.84	PA(18:0)	C21H42O7P	437.2674	1.74
455.2202	1.33	PA (20:4(OH)) -H2O	C23H36O7P	455.2204	0.46
581.3087	11.58	PI 18:0 -H2O	C27H50O11P	581.3096	1.58
599.3193	6.07	PI 18:0	C27H52O12P	599.3202	1.49
617.2729	4.8	PI 20:4(OH) -H2O	C29H46O12P	617.2732	0.55
**901.5425**	**100**	**PI(18:0/20:4(OH))**	**C47H82O14P**	**901.5448**	**2.52**

C24(OH)-Sulf. [M-H]- m/z 906.6346

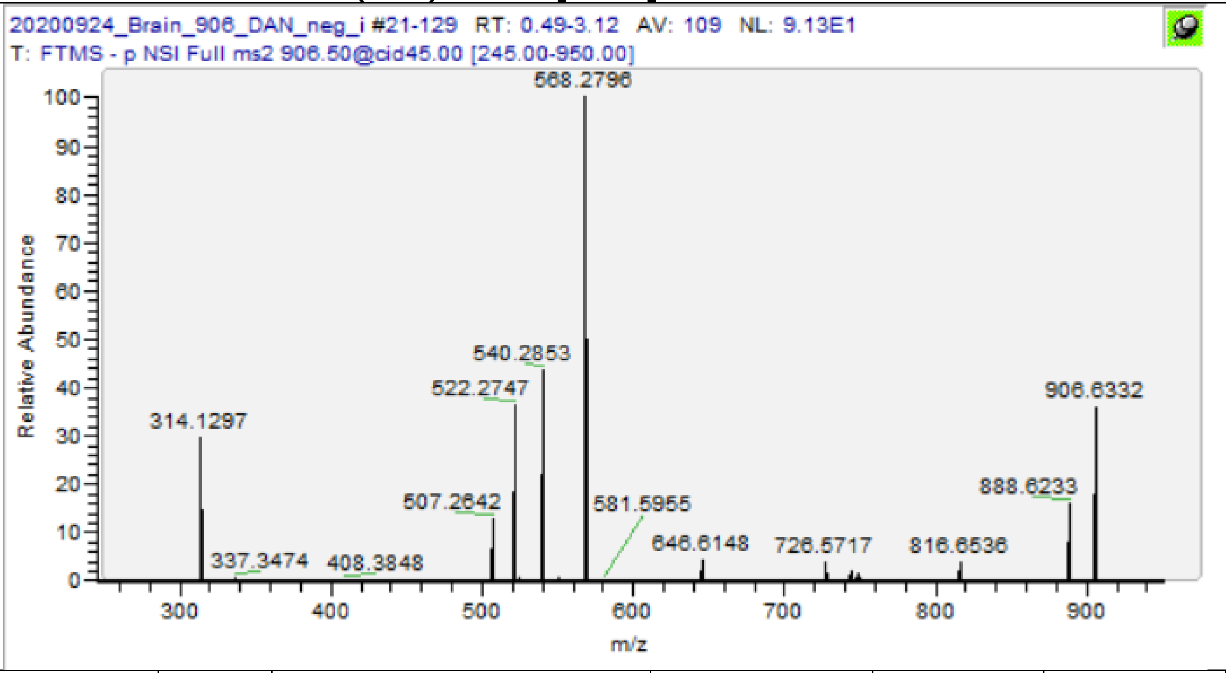

m/z	[I]	Species	Formula	Da	ppm
408.3848	0.42	C24(OH) Sulf. -Gala	C26H50O2N	408.3847	-0.24
507.2642	13.31	C24(OH) Sulf. -C24 -N -H2O	C24H43O9S	507.2633	-1.72
522.2747	39.17	C24(OH) Sulf. -C24 -H2O	C24H44O9NS	522.2742	-0.90
540.2853	46.26	C24(OH) Sulf. -C24	C24H46O10NS	540.2848	-0.94
550.2698	0.8	C24(OH) Sulf. -C23 -H2O	C25H44O10NS	550.2691	-1.20
568.2797	100	C24(OH) Sulf. -C23	C25H46O11NS	568.2797	0.02
668.4034	0.4	C24(OH) Sulf. -C16	C32H62O11NS	668.4049	2.26
888.6233	15.73	C24(OH) Sulf. -H2O	C48H90O11NS	888.624	0.80
**906.6333**	**37.56**	**C24(OH) Sulf.**	**C48H92O12NS**	**906.6346**	**1.40**

Gal-GalNAc-Gal-Glc-(d36:1) [M-H]- m/z 1254.777

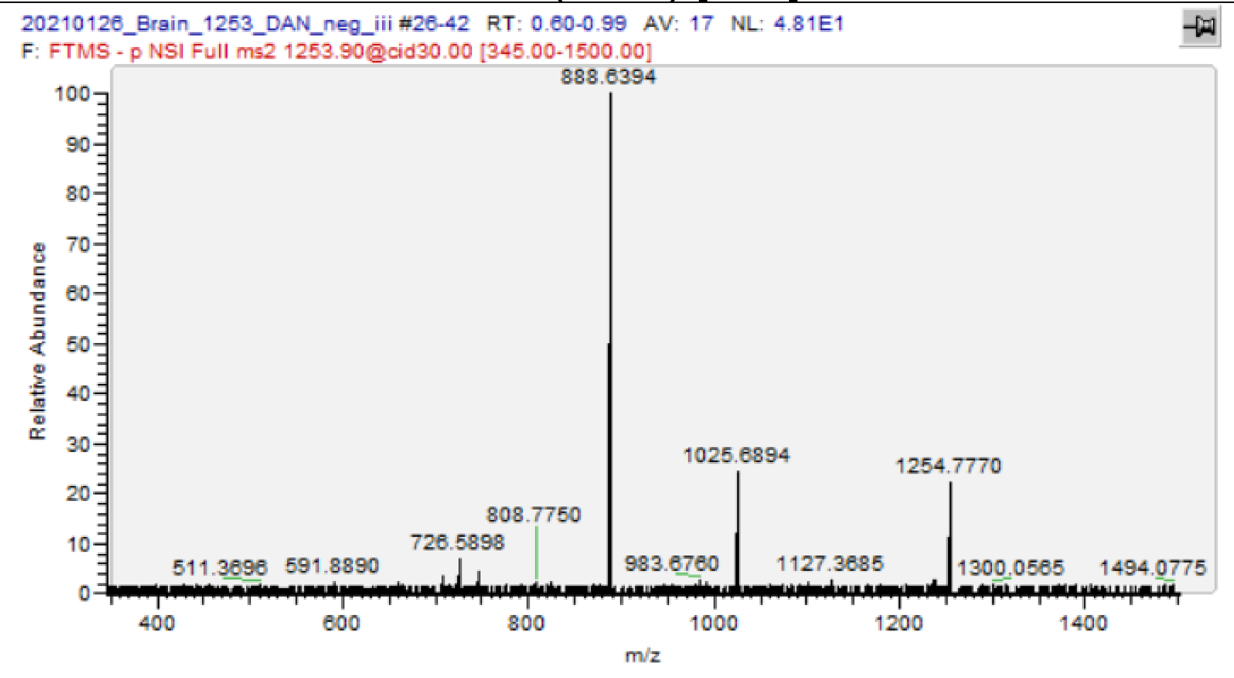

m/z	[I]	Species	Formula	Da	ppm
708.5791	3.34	GlcCer(d36:1) -H2O	C42H78O7N	708.5784	-1.02
726.589	3.74	GlcCer(d36:1)	C42H80O8N	726.5889	-0.08
888.6398	100	Gal-Glc-Cer(d36:1)	C48H90O13N	888.6418	2.22
983.6775	2.43	GalNAc-Gal-Glc-Cer(d36:1) 0, 3 -X3	C53H95O14N2	983.6789	1.40
1025.689	23.27	GalNAc-Gal-Glc-Cer(d36:1) Z3a, Z3b - COH2	C55H97O15N2	1025.689	0.04
**1254.777**	**20.12**	**Gal-GalNAc-Gal-Glc-(d36:1)**	**C62H113O23N2 iso**	**1254.777**	**0.26**

GM1(d36:1) [M-H]- m/z 1544.869

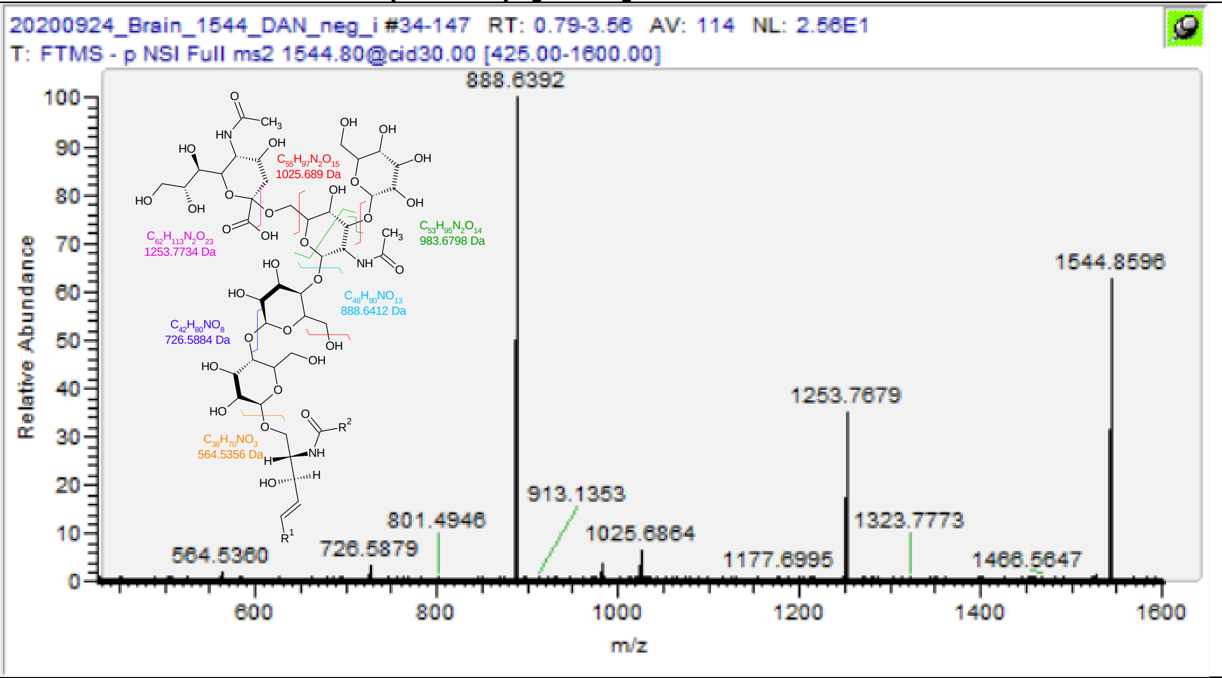

m/z	[I]	Species	Formula	Da	ppm
564.5360	1.63	Cer(d36:1)	C36H70O3N	564.5361	0.21
726.5876	3.12	GlcCer(d36:1)	C42H80O8N	726.5889	1.84
888.6396	100	Gal-Glc-Cer(d36:1)	C48H90O13N	888.6418	2.44
983.6758	4.56	GalNAc-Gal-Glc-Cer(d36:1) 0, 3 -X3	C53H95O14N2	983.6789	3.13
1025.6865	6.27	GalNAc-Gal-Glc-Cer(d36:1) Z3a, Z3b - COH2	C55H97O15N2	1025.689	2.87
1253.7679	36.67	Gal-GalNAc-Gal-Glc-(d36:1)	C62H113O23N2	1253.774	4.83
**1544.8632**	**66.08**	**GM1(d36:1)**	**C73H130O31N3**	**1544.869**	**4.00**

GM1(d38:1) [M-H]- m/z 1573.904

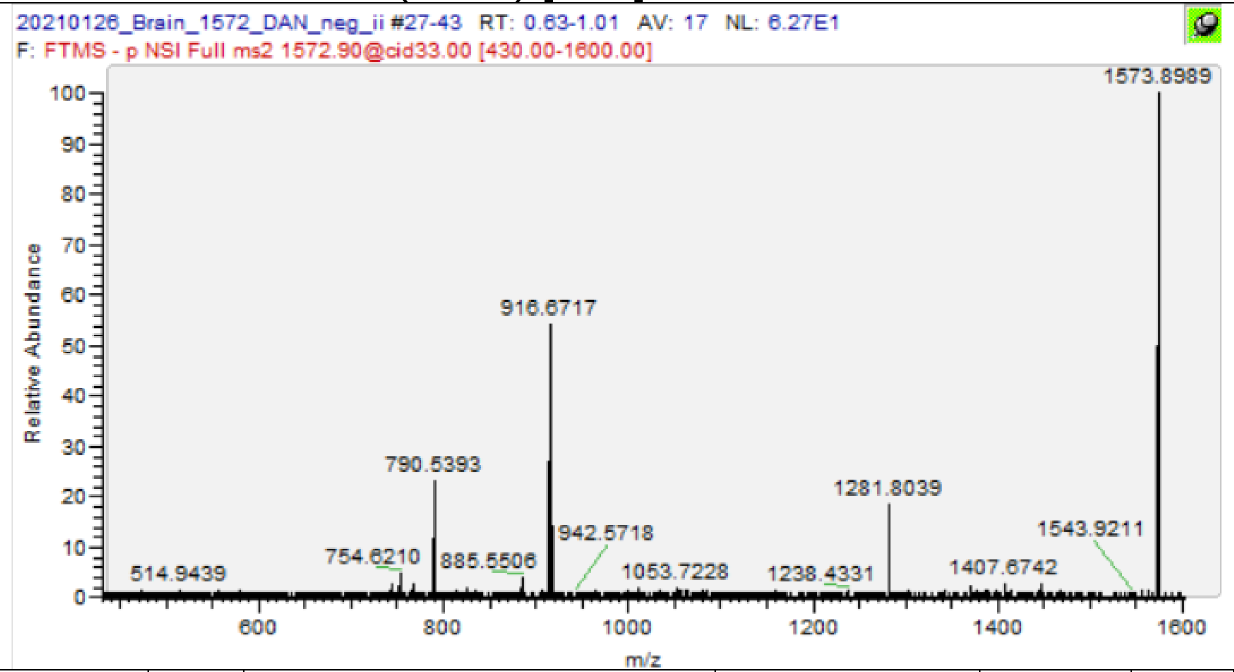

m/z	[I]	Species	Formula	Da	ppm
290.0882	34.19	NeuAc	C11H16O8N	290.0881	-0.21
754.6212	3.02	GlcCer(d38:1)	C44H84O8N	754.6202	-1.27
916.6718	53.53	Gal-Glc-Cer(d38:1)	C50H94O13N	916.6731	1.38
1011.71	1.84	GalNAc-Gal-Glc-Cer(d38:1) 0, 3 -X3	C55H99O14N2	1011.71	0.67
1053.723	2.14	GalNAc-Gal-Glc-Cer(d38:1) Z3a, Z3b -COH2	C57H101O15N2	1053.721	-1.95
1281.804	19.3	Gal-GalNAc-Gal-Glc-(d38:1)	C64H117O23N2	1281.805	1.06
**1573.899**	**100**	**GM1(d38:1)**	**C75H134O31N3 iso**	**1573.904**	**3.19**

GD1(d36:1) [M-H]- m/z 1835.9648

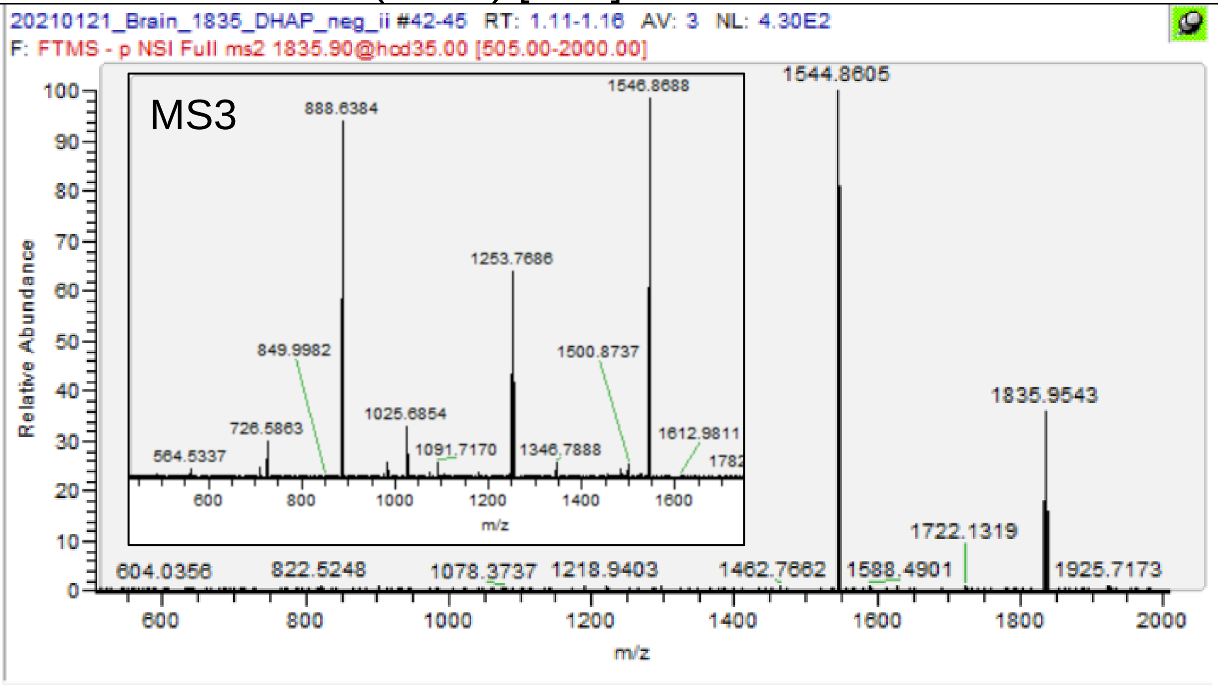

m/ z	[I]	Species	Formula	Da	ppm
564.5337**	2.16	Cer(d36:1)	C36H70O3N	564.5361	4.29
708.5759**	2.97	GlcCer(d36:1), Z	C42H78O7N	708.5784	3.50
726.5863**	9.44	GlcCer(d36:1), Y	C42H80O8N	726.5889	3.63
888.6384**	92.72	Gal-Glc-Cer(d36:1)	C48H90O13N	888.6418	3.79
983.6756**	3.9	GalNAc-Gal-Glc-Cer(d36:1) 0, 3 -X3	C53H95O14N2	983.6789	3.33
1025.6854**	13.74	GalNAc-Gal-Glc-Cer(d36:1) Z3a, Z3b -COH2	C55H97O15N2	1025.6894	3.94
1091.717**	3.98	GlcNAc-Gal-Glc-Cer(d36:1)	C56H103O18N2	1091.7211	3.79
1253.7686**	54.36	Gal-GalNAc-Gal-Glc-(d36:1)	C62H113O23N2	1253.7740	4.28
1544.8631*	100	GM1(d36:1)	C73H130O31N3	1544.8694	4.07
**1835.957***	**32.92**	**GD1(d36:1)**	**C84H147O39N4**	**1835.9648**	**4.24**
* MS2@1835.9					
** MS3@1544.8					

GD1(d36:1) [M-2H+K]- m/z 1873.921

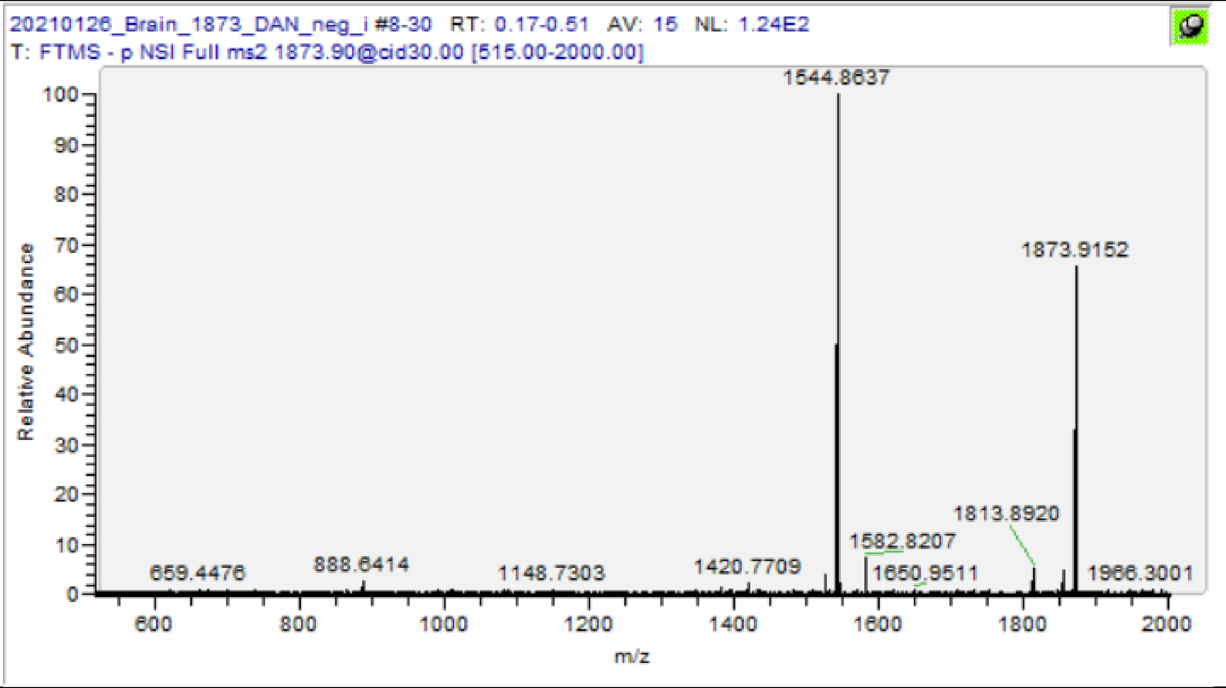

m/z	[I]	Species	Formula	Da	ppm
888.6414	2.64	Gal-Glc-Cer(d36:1)	C48H90O13N	888.6418	0.42
1253.773	3.99	Gal-GalNAc-Gal-Glc-(d36:1)	C62H113O23N2	1253.774	0.85
1544.864	100	GM1(d36:1)	C73H130O31N3	1544.869	3.61
1582.821	7.25	GM1(d36:1)+K	C73H129O31N3K	1582.825	2.88
1813.892	5.24	GD1(d36:1)+K-C2H5O	C82H142O37N4K	1813.9	4.16
1855.903	5.53	GD1(d36:1)+K-H2O	C84H144O38N4K	1855.91	3.62
**1873.916**	**88.88**	**GD1(d36:1)+K**	**C84H146O39N4K**	**1873.921**	**2.71**
